# Axon guidance cue SLIT2 regulates the murine skeletal stem cell niche through sympathetic innervation

**DOI:** 10.1172/JCI193014

**Published:** 2025-10-15

**Authors:** Zuoxing Wu, Na Li, Zhengqiong Luo, Zihan Chen, Xuemei He, Jie Han, Xixi Lin, Fan Shi, Haitao Huang, Baohong Shi, Yu Li, Xin Wang, Lin Meng, Dachuan Zhang, Lanfen Chen, Dawang Zhou, Weinan Cheng, Matthew B. Greenblatt, Ren Xu

**Affiliations:** 1 The First Affiliated Hospital of Xiamen University–ICMRS Collaborating Center for Skeletal Stem Cells, Xiamen Cell Therapy Research Center, First Affiliated Hospital of Xiamen University, School of Medicine, Faculty of Medicine and Life Sciences, and; 2 Xiamen Key Laboratory of Regeneration Medicine, Fujian Provincial Key Laboratory of Organ and Tissue Regeneration, School of Medicine, Xiamen University, Xiamen, China.; 3 Shenzhen Key Laboratory of Bone Tissue Repair and Translational Research, Department of Orthopaedic Surgery, Seventh Affiliated Hospital of Sun Yat-sen University, Shenzhen, China.; 4 Research Centre for Regenerative Medicine, Guangxi Key Laboratory of Regenerative Medicine, Guangxi Medical University, Nanning, China.; 5 State Key Laboratory of Cellular Stress Biology, Innovation Center for Cell Signaling Network, School of Life Sciences, Xiamen University, Xiamen, China.; 6 Graduate School of Science and Engineering and; 7 College of Science and Engineering, Ritsumeikan University, Shiga, Japan.; 8 Department of Pathophysiology, Key Laboratory of Cell Differentiation and Apoptosis, Chinese Ministry of Education, Shanghai Jiao Tong University School of Medicine, Shanghai, China.; 9 Department of Sports Medicine, National Center for Orthopaedics, Shanghai Jiao Tong University Affiliated Sixth People’s Hospital, Shanghai, China.; 10 Department of Pathology and Laboratory Medicine, Weill Cornell Medical College, New York, New York, USA.; 11 Skeletal Health and Orthopedic Research Program, Hospital for Special Surgery, New York, New York, USA.

**Keywords:** Bone biology, Neuroscience, Bone development

## Abstract

Sympathetic tone is a central signaling axis inhibiting osteogenesis; however, the combination of durable local and systemic sympathetic effects on bone argues that multiple mechanisms, including yet-undiscovered pathways, are involved. Here, we found that sympathetic nerves constituted a component of the skeletal stem cell (SSC) niche: mice with conditional deletion of the classical axonal repellent *Slit2* in sympathetic nerves (*Slit2^th^* mice), but not in bone stem/progenitor cells or sensory nerves, showed osteopenia due to an increase in sympathetic innervation and an associated decrease in SSCs. Mice with increased skeletal sympathetic innervation displayed impaired SSC niche function in an SSC orthotopic transplantation and engraftment system. Follistatin-like 1 (FSTL1) is a SLIT2-regulated soluble factor suppressing SSC self-renewal and osteogenic capacity. Accordingly, ablation of *Fstl1* in sympathetic neurons enhanced SSC-driven osteogenesis and attenuated the bone loss seen in *Slit2^th^* mice. Together, the findings indicate that SLIT2 is a regulator of a sympathetic nerve–mediated SSC niche.

## Abstract 2

SLIT2-mediated sympathetic innervation regulates the fate of skeletal stem cells.

## Graphical abstract

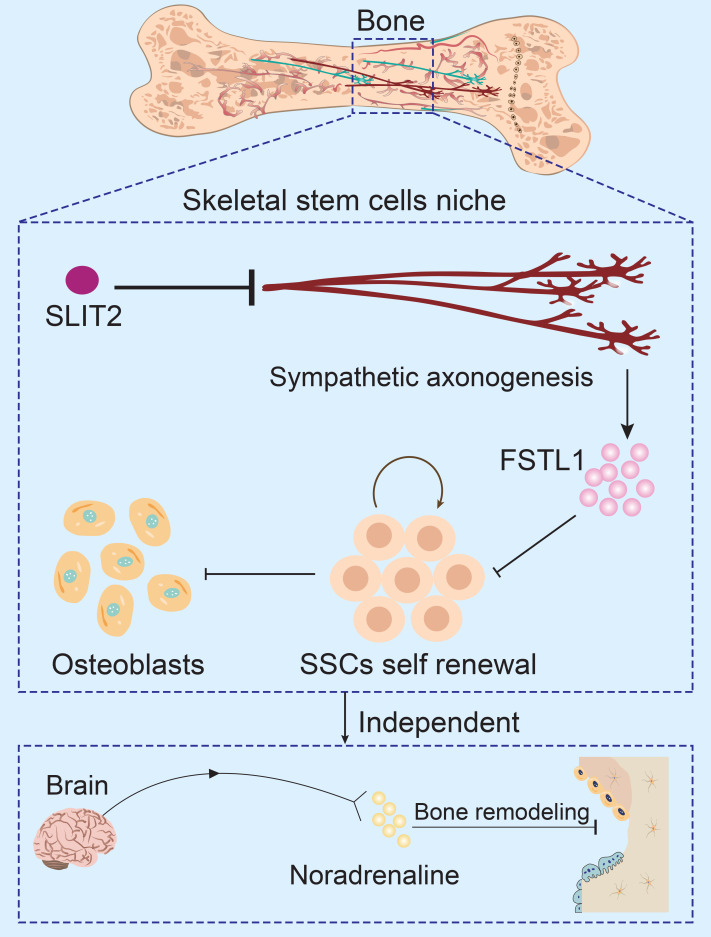

## Introduction

The fates of tissue-resident stem cells are often determined by a specialized local microenvironment, termed the niche, that is tailored to provide the physical interactions and secreted cues regulating the stem cell compartment (1, 2). Deciphering the constituents of the stem cell niche has proven to be a key part of understanding both normal physiology and pathology in multiple organ systems, which has facilitated the development of cell therapy applications (3). Skeletal stem cells (SSCs) have been identified only within the last decade as the origin of bone-forming osteoblasts and are identified through a defined set of surface markers (4, 5). However, compared with other adult stem cells such as melanocyte stem cells, the anatomic and functional components of the SSC niche are still unclear, largely due to the recency of SSC identification and the absence of orthotopic engraftment assays to study SSCs that parallel assays classically used to establish niche effects in hematopoietic stem cells (6–9).

In fact, bone-specific ancillary tissues such as nerve subtypes and vascular endothelium have been suggested to play a critical role in bone growth, homeostasis, and regeneration (10–13). Our prior study demonstrated that osteoblast-derived SLIT3, an axon guidance cue first studied in the nervous system, can induce formation of bone-specific CD31^hi^EMCN^hi^ endothelium, which in turn promotes osteogenesis. Consistent with this, deletion of *Slit3* in osteoblasts led to severe osteopenia and a paucity of skeletal vascular endothelium (14, 15). In studying SLIT3, we observed that SLIT2, another SLIT ligand, is also expressed at high levels by both neurons and osteoblasts (16).

As a classical chemorepellent factor, soluble SLIT2 actively controls axonal outgrowth and neuron migration through receptors of the Roundabout (Robo) family (17, 18). This repulsive signal is crucial for neural development. For instance, global SLIT2-deficient mice display a neonatal lethality phenotype due to several neuronal defects, including abnormalities in spiral ganglion neurons and retinal ganglion cells and motor axon fasciculation (19–21). In addition, SLIT2 plays multiple physiological roles outside of the nervous system, including involvement in angiogenesis, metabolism, and cancer metastasis (22–24). Nevertheless, the function of SLIT2 in skeletal innervation and the corresponding impact on osteogenesis remain largely unexplored.

Given the observation that SLIT2 and SLIT3 have similar proangiogenic activity, we first evaluated whether osteoblast-derived SLIT2 would maintain bone formation through targeting vascular endothelium similarly to SLIT3 (14). Unexpectedly, neither SSC-specific deletion of *Slit2* by use of *Prrx1*-Cre (25) nor osteolineage deletion by use of Osx-Cre (16) led to detectable bone loss. In contrast, specific deletion of *Slit2* in neurons caused severe osteopenia due to impaired bone formation. In parallel with this, *Slit2* ablation in the nervous system specifically disrupted skeletal sympathetic but not sensory innervation. Recently, sympathetic innervation has been characterized to drive stem cell fate as the key regulatory niche unit in non-bone tissues (26, 27). While a series of previous studies have established that sympathetic activity regulates osteoblast activity (28–34), longstanding clinical observations indicate that local autonomic dysregulation, such as in complex regional pain syndrome, produces a tightly localized, long-term, and progressive bone loss (35–37). The observation that sympathetic dysregulation can produce a wide range of low-bone-mass phenotypes, including both systemic and local, progressive forms, led us to hypothesize that sympathetic regulation of bone mass may include additional fundamental mechanisms beyond the well-characterized effects of catecholamines on skeletal cells. In particular, the association of sympathetic activation with local, progressive bone loss raised in particular the possibility that sympathetic nerves may be able to directly regulate the SSC niche. Hence, we herein sought to use SLIT2-deficient mice as a tool to establish the contribution of peripheral nerves to the SSC niche.

## Results

### Nerve-derived but not bone-derived SLIT2 regulates bone formation.

To understand the role of SLIT2 in bone homeostasis, we first examined *Slit2* expression in osteoblasts and osteoclasts. Similar to SLIT3 (15), robust expression of SLIT2 was detected in osteoblasts but not osteoclasts at both the mRNA and protein level (Supplemental Figure 1, A and B; supplemental material available online with this article; https://doi.org/10.1172/JCI193014DS1). Based on this, we bred *Slit2^fl/fl^* mice to Cre driver lines targeting preosteoblasts/osteoblasts (Osx-Cre; *Slit2^osx^* mice) (Supplemental Figure 1, C and D). Unexpectedly, *Slit2^osx^* mice displayed similar bone mass as littermate *Slit2^fl/fl^* mice or Osx-Cre control mice (Supplemental Figure 1, E and F). Consistent with this, there were no observable changes in the amount of CD31^hi^EMCN^hi^ vascular endothelium in bones of *Slit2^osx^* mice (Supplemental Figure 1, G and H). In addition, primary osteoblast culture ex vivo showed that ablation of *Slit2* did not impair osteoblast differentiation or mineralization (Supplemental Figure 1, I and J). Similarly, deletion of *Slit2* in skeletal stem/progenitor cells using *Prrx1*-Cre (*Slit2^prx1^* mice) did not result in a skeletal phenotype (Supplemental Figure 2, A–D). These results together indicated that SLIT2 expression in skeletal lineage cells was dispensable for osteogenesis.

**Figure 1:**
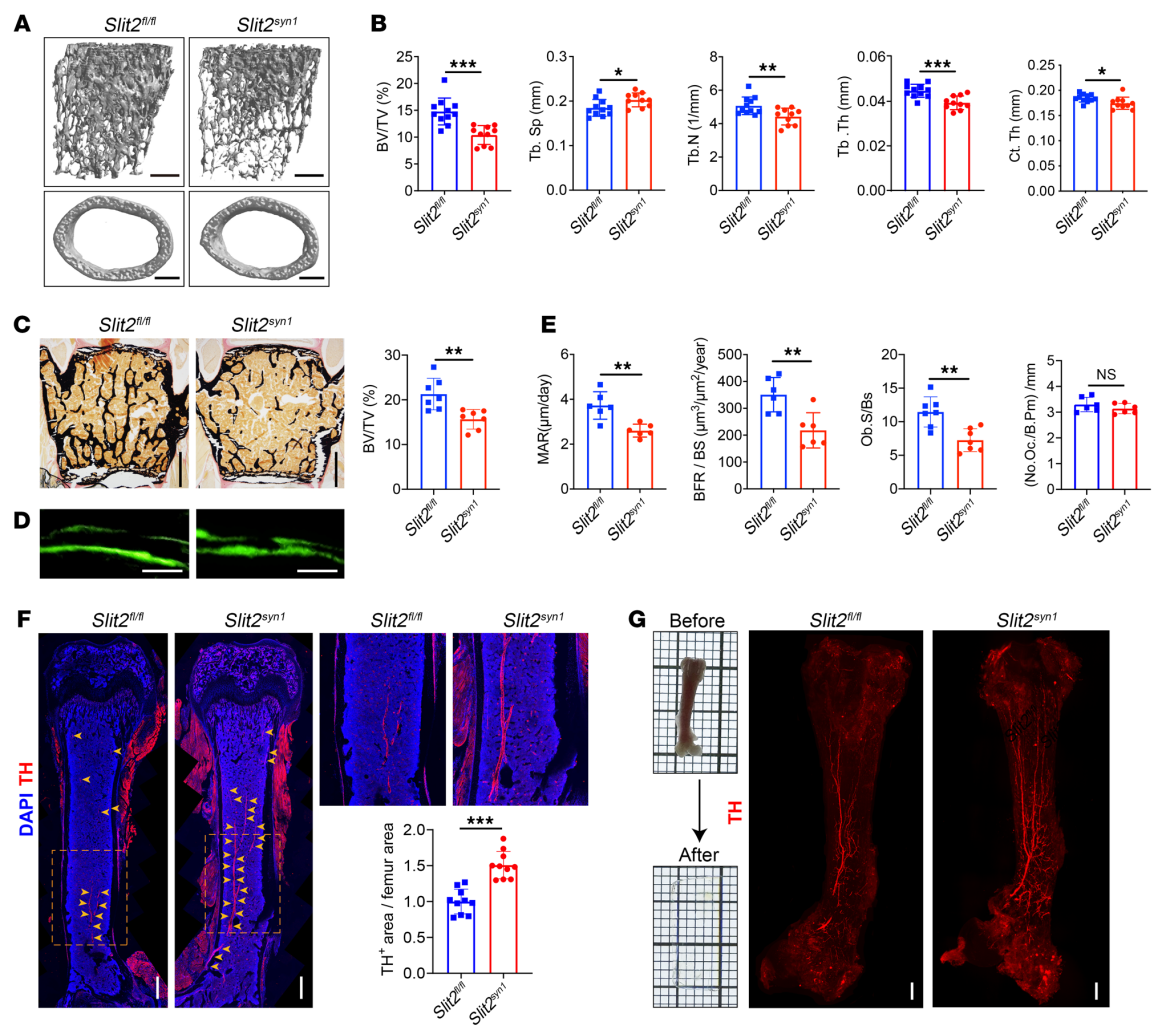
Bone loss, impaired bone formation, and sympathetic hyperinnervation in *Slit2^syn1^* mice. (**A** and **B**) Representative μCT images of trabecular bone in the distal femur (**A**) and BV/TV with relative quantitative analysis of bone parameters (**B**) in *Slit2^fl/fl^* and *Slit2^syn1^* male mice at 8 weeks of age. *Slit2^fl/fl^*, *n* = 11; *Slit2^syn1^*, *n* = 10. Scale bars: 500 μm. (**C**) Representative images of Von Kossa/Van Gieson staining and BV/TV of the L3 vertebral bone in 8-week-old *Slit2^fl/fl^* and *Slit2^syn1^* male mice. *n* = 7 per group. Scale bars: 500 μm. (**D** and **E**) Representative images of calcein double labeling (**D**) and quantification of histomorphometric parameters of the L3 vertebrae (**E**) in *Slit2^fl/fl^* and *Slit2^syn1^* male mice at 8 weeks of age. MAR, trabecular mineral apposition rate (μm/day); BFR/BS, bone formation rate/bone surface (μm^3^/μm/yr); Ob.S/BS, osteoblast surface/bone surface (%); (No.Oc./B.Pm, osteoclast number/bone perimeter. MAR and BFR/BS: *n* = 6 per group; Ob.S/BS: *n* = 7 per group; No.Oc./B.Pm: *n* = 6 per group. Scale bars: 100 μm. (**F**) Representative confocal images of immunofluorescence staining with TH (red) and DAPI (blue) and quantitative analysis of relative quantity of TH^+^ sympathetic nerves in femur sections from 3-week-old *Slit2^fl/fl^* and *Slit2^syn1^* male mice. Arrowheads indicate TH^+^ sympathetic nerve fibers. *n* = 10 per group. Scale bars: 400 μm. (**G**) Images of tibiae prior to and after tissue clearing. Representative images of whole-tissue TH immunofluorescence labeling of femurs from 3-week-old *Slit2^fl/fl^* and *Slit2^syn1^* male mice. Scale bars: 400 μm. Error bars indicate mean ± SEM. *P < 0.05, **P < 0.01, ***P < 0.001, by unpaired, 2-tailed Student’s *t* test for 2-group comparisons.

Bone homeostasis has been shown to be regulated by the central and peripheral nervous systems (10). As SLIT2 mediates the spatial and temporal patterning of innervation during organogenesis as an axonal chemorepellent (18), we next assessed the necessity of nervous system–derived SLIT2 in the maintenance of bone mass. To examine this, we intercrossed *Slit2^fl/fl^* mice with synapsin-I–Cre mice to specifically delete Slit2 in neurons (*Slit2^syn1^* mice) but not skeletal cells, as validated by lineage tracing and SLIT2 expression analysis (Supplemental Figure 3, A–C). Contrasting with the results of conditional *Slit2* deletion in skeletal cells, a substantial reduction in bone mass was seen in both vertebrae and long bones of *Slit2^syn1^* mice relative to littermate *Slit2^fl/fl^* controls (Figure 1, A–C). Histomorphometric analysis further showed that the osteopenic phenotype observed in *Slit2^syn1^* mice was mainly due to reductions in bone formation and the numbers of mature osteoblasts in vivo (Figure 1, D and E). At the same time, both the number of osteoclasts and serum CTX levels remained unchanged in *Slit2^syn1^* mice (Figure 1E and Supplemental Figure 3D). In addition, neither global deletion of *Slit1* (Supplemental Figure 4, A and B) nor neuron-specific deletion of *Slit3* resulted in an observable alteration of bone mass in vivo (14). Taken together, the results showed that among the SLIT ligands, only SLIT2 functioned in neurons to regulate bone mass accrual by supporting in vivo bone formation.

### SLIT2-regulated sympathetic innervation controls bone formation.

The skeleton is innervated by sympathetic and sensory nerves (38, 39), with sympathetic nervous activity stimulating bone resorption and suppressing bone formation (40–43). To determine whether lack of *Slit2* in the nervous system disrupts peripheral bone innervation, we further analyzed the nerve subtypes present in the bones of *Slit2^syn1^* mice. Interestingly, both immunostaining of femur sections and 3D imaging of optically cleared bones showed that the level of tyrosine hydroxylase positive (TH-positive) sympathetic nerve fibers was elevated in *Slit2^syn1^* mice relative to littermate controls, whereas calcitonin gene–related peptide–positive (CGRP-positive) sensory fibers were not altered (Figure 1, F and G, and Supplemental Figure 5A). Thus, sympathetic but not sensory innervation of the skeleton was regulated by *Slit2* expression in neurons.

**Figure 2:**
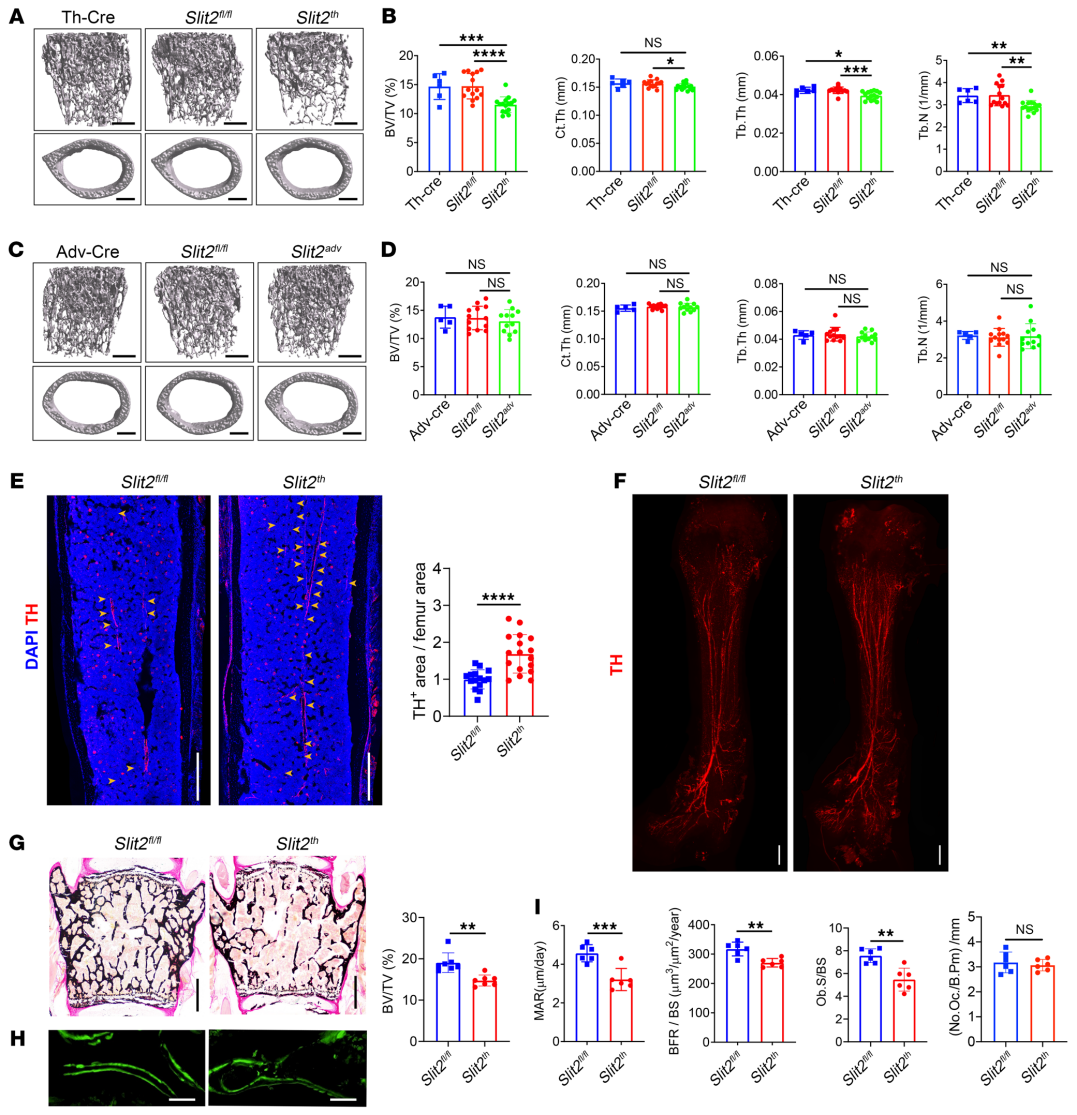
*Slit2^th^* but not *Slit2^adv^* mice recapitulate the skeletal and nerve phenotypes of *Slit2^syn1^* mice. (**A** and **B**) Representative μCT images of trabecular bone in the distal femur (**A**) and relative quantitative analysis of BV/TV and relative quantitative analysis of bone parameters (**B**) in male control (Th-cre and *Slit2^fl/fl^*) and *Slit2^th^* mice at 8 weeks of age. Th-cre, *n* = 6; *Slit2^fl/fl^*; *n* = 14; *Slit2^th^*; *n* = 15. Scale bars: 500 μm. (**C** and **D**) Representative μCT images of trabecular bone in the distal femur (**C**) and relative quantitative analysis of BV/TV and of bone parameters (**D**) in male control (Adv-cre and *Slit2^fl/fl^*) and *Slit2^adv^* mice at 8 weeks of age. Adv-cre, *n* = 5; *Slit2^fl/fl^*, *n* = 13; *Slit2^adv^*. *n* = 12. Scale bars: 500 μm. (**E**) Representative confocal images of immunofluorescence staining with TH (red) and DAPI (blue) and quantitative analysis of relative TH^+^ sympathetic nerves in femur sections from 3-week-old *Slit2^fl/fl^* and *Slit2^th^* male mice. *Slit2^fl/fl^*, *n* = 15; *Slit2^th^*. *n* = 17. Scale bars: 500 μm. (**F**) Representative images of whole-femur immunofluorescence labeling of TH^+^ sympathetic nerves in 3-week-old *Slit2^fl/fl^* and *Slit2^th^* male mice. Scale bars: 500 μm. (**G**) Representative images of Von Kossa/Van Gieson staining and BV/TV of the L3 vertebral bone in 8-week-old *Slit2^fl/fl^* and *Slit2^th^* male mice. *n* = 7 per group. Scale bars: 500 μm. (**H** and **I**) Representative images of calcein double labeling (**H**) and quantification of histomorphometric parameters of L3 vertebrae in *Slit2^fl/fl^* and *Slit2^th^* male mice at 8 weeks of age. MAR trabecular mineral apposition rate (μm day^−1^); BFR/BS, bone formation rate/bone surface (μm^3^μm^−2^yr^−1^); Ob.S/BS, osteoblast surface/bone surface (%); No.Oc./B.Pm, osteoclast number/bone perimeter (**I**). MAR and BFR/BS: *n* = 6 per group; Ob.S/BS and No.Oc./B. Pm: *n* = 6 per group; Scale bars: 100 μm. Error bars indicate mean ± SEM. *P < 0.05, **P < 0.01, ***P < 0.001, ****P < 0.0001, by ordinary 1-way ANOVA for multiple-group comparisons (**B** and **D**). *P < 0.05, **P < 0.01, ***P < 0.001, ****P < 0.0001, by unpaired, 2-tailed Student’s *t* test for 2-group comparisons (**E**,**G**, and **I**).

We next sought to further dissect whether sympathetic or sensory nerves are the key source of SLIT2 regulation of the skeleton. Immunofluorescence analysis revealed colocalization of Slit2 expression with both sympathetic and sensory nerve fibers in bone (Supplemental Figure 6, A and B). To this end, we conditionally deleted *Slit2* in either sympathetic or sensory nerves using TH-Cre (44) or advillin-Cre (45), respectively, in *Slit2^th^* or *Slit2^adv^* mice. The specificity and efficiency of *Slit2* deletion was validated by reporter mice and Slit2 expression analysis in both crosses (Supplemental Figure 6, C–H). Interestingly, μCT analysis and bone histology demonstrated that *Slit2^th^* but not *Slit2^adv^* mice displayed an osteopenic phenotype similar to that observed in *Slit2^syn1^* mice (Figure 2, A–D, and Supplemental Figure 7, A and B). Notably, this bone phenotype was consistently observed in both male and female mice, indicating that the skeletal effects of sympathetic nerve–specific Slit2 deletion were not sex dependent (Supplemental Figure 7C). In parallel with this, both immunostaining of femur sections and 3D imaging of optically cleared bones showed that the number of TH-positive nerve fibers was considerably elevated in *Slit2^th^* but not *Slit2^adv^* mice (Figure 2, E and F, and Supplemental Figure 7, D–F). Deletion of *Slit2* did not induce a compensatory increase in expression levels of other SLITs in sympathetic neurons (Supplemental Figure 8, A–C) or lead to alterations in brain morphology (Supplemental Figure 8D). Consistent with observations in *Slit2^syn1^* mice, the number of CGRP-positive nerve fibers was not significantly altered in *Slit2^th^* mice (Supplemental Figure 9A). The increase in sympathetic innervation in *Slit2^th^* mice resulted in impaired bone formation but not bone resorption, as shown by dynamic histomorphometric analysis and the unchanged serum CTX levels (Figure 2, G–I, and Supplemental Figure 9B). Together, the results indicate that sympathetic but not sensory neuron–derived SLIT2 suppresses both skeletal sympathetic innervation and bone mass accrual in vivo.

### Sympathetic nerves regulate the SSC niche.

**Figure 3:**
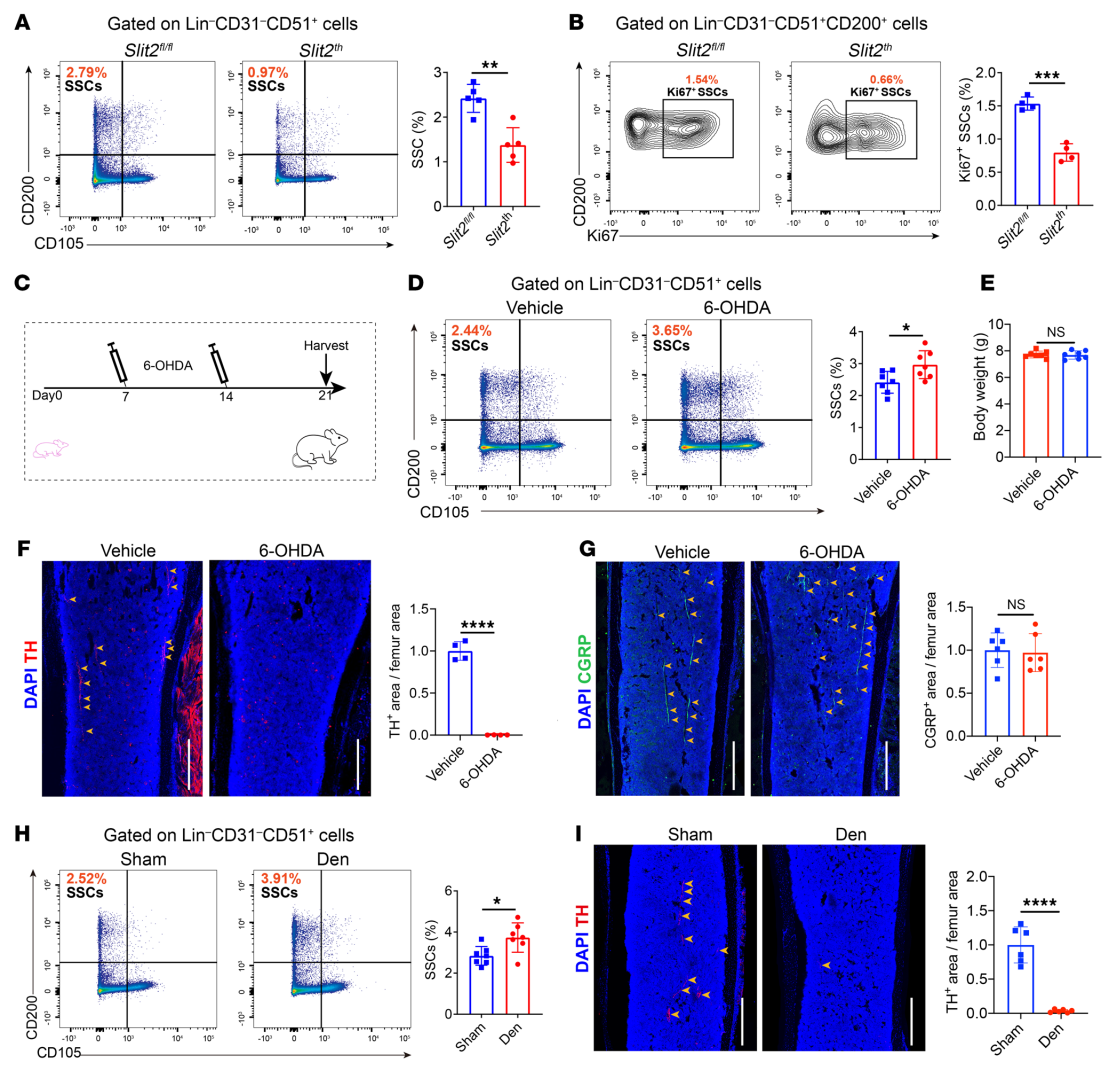
Sympathetic innervation negatively controls SSC abundance. (**A**) Representative flow cytometry plots and relative frequency of SSCs from femurs of 3-week-old *Slit2^fl/fl^* and *Slit2^th^* male mice. *n* = 5 per group. (**B**) Representative flow cytometry and quantitative analysis of the relative frequency of Ki-67^+^ fractions of SSCs (expressed as a proportion of the total Lin-CD31-CD51+ progenitor population) isolated from the femurs of 3-week-old *Slit2^fl/fl^* and *Slit2^th^* male mice. *n* = 4 per group. (**C**) Timeline of the 6-OHDA chemical sympathectomy mouse models. (**D**) Representative flow cytometry plots and relative frequency of SSCs in the femurs of 3-week-old WT male mice treated with 6-OHDA or vehicle as neonates. *n* = 7 per group. (**E**) Statistical analysis of body weight of 3-week-old WT male mice treated with 6-OHDA or vehicle as neonates. *n* = 7 per group. (**F**) Representative confocal images of immunofluorescence staining with TH (red) and DAPI (blue) and quantitative analysis of TH^+^ sympathetic nerves in femur sections from 3-week-old WT male mice treated with 6-OHDA or vehicle at the neonatal stage. *n* = 4 per group. Scale bars: 500 μm. (**G**) Representative confocal images of immunofluorescence staining with CGRP (green) and DAPI (blue) and quantitative analysis of CGRP^+^ sensory nerves in femur sections from 3-week-old WT male mice treated with 6-OHDA or vehicle as neonates. *n* = 6 per group. Scale bars: 500 μm. (**H**) Representative flow cytometry showing the relative frequency of femur SSCs in 3-week-old WT male mice after sympathectomy surgery. *n* = 7 per group. Sham, sham surgery group; Den, denervation group. (**I**) Representative confocal images of immunofluorescence staining with TH and DAPI and quantitative analysis of TH^+^ sympathetic nerves in femur sections from 3-week-old WT male mice after sympathectomy surgery. *n* = 6 per group. Scale bars: 500 μm. Error bars indicate mean ± SEM. *P < 0.05, **P < 0.01, ***P < 0.001, ****P < 0.0001, by unpaired, 2-tailed Student’s *t* test for 2-group comparisons.

While sympathetic innervation decreased bone formation, it is unclear whether the autonomic nervous system acts at the level of regulating the SSC niche or through other mechanisms. Indeed, 3D imaging of optically cleared skeletons identified skeletal sympathetic nerve fibers in close proximity to physical sites housing SSCs, such as the metaphyseal region, periosteum, and inner endosteal compartment (Supplemental Figure 10, A–D) (5, 46, 47). To address the impact of sympathetic hyperinnervation of SSCs, we evaluated SSCs in the long bones of *Slit2^syn1^* mice and *Slit2^th^* mice, defining SSCs as CD45^−^Ter119^−^CD31^−^AlphaV^+^Thy^−^6C3^−^CD105^−^CD200^+^ cells, a population previously shown to display formal evidence of stemness in several contexts (4, 5, 48, 49). The abundance of immunophenotypic SSCs was reduced in both of *Slit2^syn1^* and *Slit2^th^* mice displaying augmented sympathetic innervation, whereas the abundance of non-stem progenitors derived from SSCs, pre-bone cartilage stromal progenitors (pre-BCSPs) and BCSPs, was not significantly decreased (Figure 3A and Supplemental Figure 11, A–E). In addition, the number of periosteal stem cells (PSC) (5) was also decreased in the periosteum of *Slit2^th^* mice (Supplemental Figure 12A). SSC proliferation was reduced in line with the reduction in total SSC pool size (Figure 3B and Supplemental Figure 12B). Thus, sympathetic hyperinnervation driven by *Slit2* loss led to a contraction in the SSC pool, consistent with a defect in SSC self-renewal. Furthermore, immunofluorescence demonstrated that the sympathetic hyperinnervation phenotype seen in *Slit2^syn1^* and *Slit2^th^* mice was progressively enhanced with increasing postnatal age, paralleling the kinetics of the decline in SSC numbers (Supplemental Figure 13, A and B) (50).

To directly confirm the causal relationship between sympathetic nerves and total SSC pool size, we conducted pharmacological sympathectomy with neonatal 6-hydroxydopamine (6-OHDA) treatment. 6-OHDA not only impaired skeletal sympathetic — but not sensory — innervation but also significantly elevated numbers of SSCs, without overt systemic or growth effects such as an impact on body weight (Figure 3, C–G). Similarly, surgical ablation of the sympathetic nerve chain (51) in both *Slit2^th^* and WT mice also impaired bone sympathetic innervation and significantly increased the number of SSCs (Figure 3, H and I, and Supplemental Figure 14, A and B).

**Figure 4:**
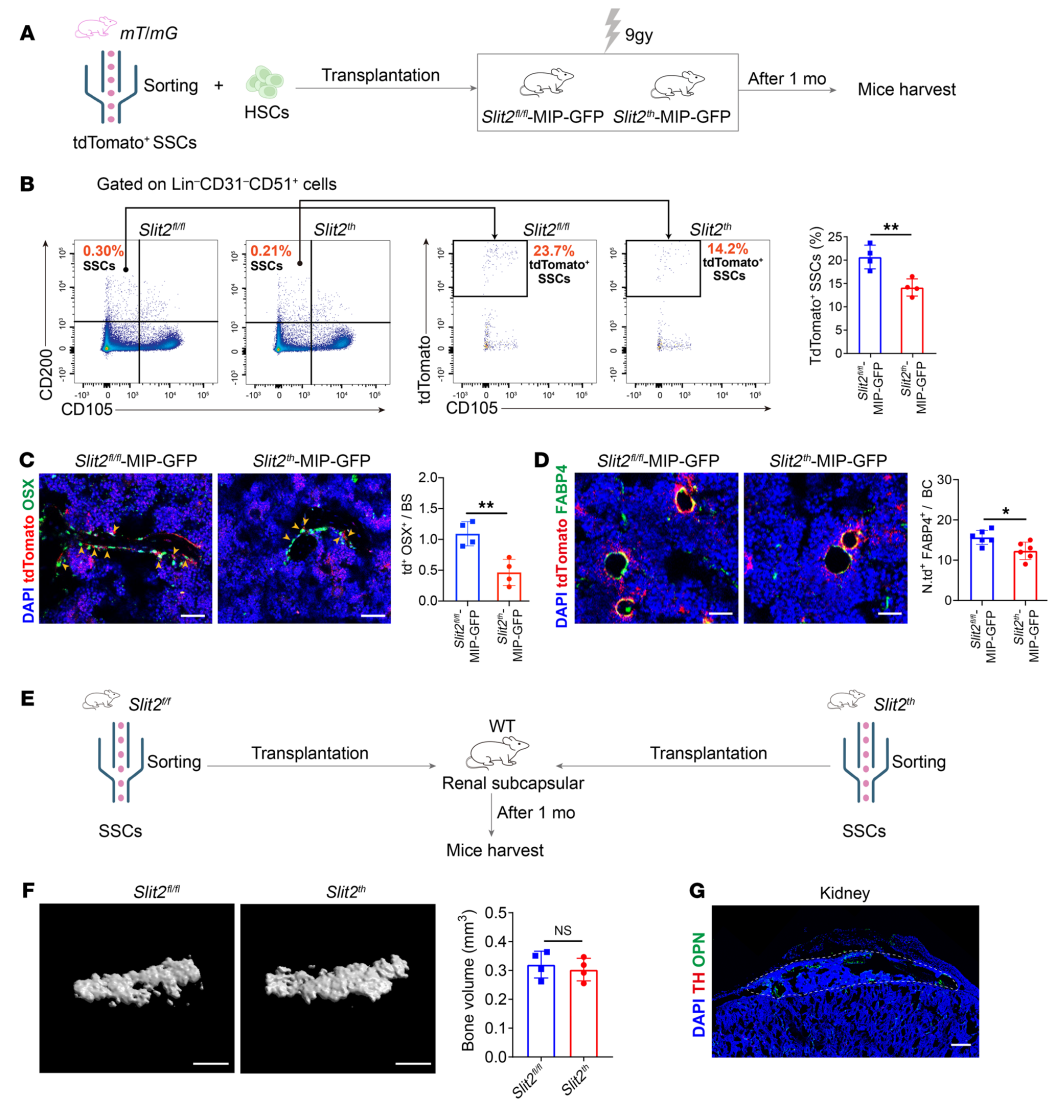
Sympathetic nerves are a component of the SSC niche. (**A**) Experimental model of mice bone marrow cavity orthotopic transplantation for SSCs. Approximately 2 × 10^4^ SSCs and 1 × 10^7^ bone marrow cells (after RBC lysis) were transplanted into each recipient mouse. **(AUTHOR: You may wish to clarify the label “Mice harvest” in A and E. Do you mean “Organoid harvest”?)** (**B**) Representative flow cytometry plots and relative frequency of tdTomato^+^ SSCs from the femurs of *Slit2^fl/fl^*-MIP-GFP and *Slit2^th^*-MIP-GFP male mice 1 month after surgery. *n* = 4 per group. (**C**) Representative images and quantitative analysis of OSX^+^ osteoblast immunofluorescence staining in the femurs of *Slit2^fl/fl^*-MIP-GFP and *Slit2^th^*-MIP-GFP male mice 4 weeks after tdTomato^+^ SSC bone marrow cavity transplantation. td^+^ OSX^+^/BS, tdTomato^+^ OSX^+^/bone area. *n* = 4 per group. Scale bars: 100 μm. (**D**) Representative images and quantitative analysis of FABP4 immunofluorescence to visualize adipocytes in femurs of *Slit2^fl/fl^*-MIP-GFP and *Slit2^th^*-MIP-GFP male mice 4 weeks after orthotopic bone marrow cavity transplantation of tdTomato^+^ SSCs. N.td^+^ FABP4^+^/BC, number of tdTomato^+^FABP4^+^/bone cavity. *n* = 6 per group. Scale bars: 100 μm. (**E**) Experimental model of mouse renal subcapsular transplantation of SSCs. Approximately 1 × 10^4^ SSCs were transplanted beneath the renal capsule on one side of each recipient mouse. (**F**) Representative images of μCT and quantitative analysis of bone parameters 4 weeks after subcapsular SSCs transplantation in mouse kidney. *n* = 4 per group. Scale bars: 500 μm. (**G**) Immunofluorescence staining for osteopontin and TH in bone organoids derived from SSCs transplanted into the renal capsule of secondary recipient mice. Organoids were harvested 4 weeks after transplantation. Scale bars: 200 μm. Error bars indicate mean ± SEM. *P < 0.05, **P < 0.01, by unpaired, 2-tailed Student’s *t* test for 2-group comparisons.

To directly determine the importance of sympathetic nerves as a component of the SSC niche, we developed an orthotopic SSC niche engraftment assay to test for SSC niche function in the native femoral environment. Specifically, we isolated tdTomato^+^ SSCs by FACS and transplanted equal numbers into femurs of sublethally irradiated *Slit2^th^* and *Slit2^fl/fl^* mice via intrafemoral injection (Figure 4A and Supplemental Figure 14C). By histology, a significant proportion of SSC-graft derived cells were found to be located in proximity (<50 μm) to TH-positive sympathetic nerves in the bone compartment of *Slit2^th^* and *Slit2^fl/fl^* mice (Supplemental Figure 14, D and E). Four weeks later, flow cytometry demonstrated that the engraftment and self-renewal of graft SSCs was reduced by 30% in the femurs of *Slit2^th^* hosts compared with *Slit2^fl/fl^* controls, accompanied by a decline in the abundance of downstream tdTomato^+^ BCSPs derived from graft SSCs (Figure 4B and Supplemental Figure 14F). Consistent with this finding, graft SSCs in the femurs of *Slit2^th^* hosts showed a significant decrease in generation of mature osteoblast derivatives, accompanied by a marginal decrease in mature adipocytes (Figure 4, C and D). On the other hand, SSCs were sorted from *Slit2^th^* and *Slit2^fl/fl^* mice, and equal numbers were transplanted into the kidney capsule of secondary WT hosts, where they displayed similar bone-forming capability (Figure 4, E and F). Consistent with this finding, SSCs from both groups also exhibited comparable CFU formation and osteogenic differentiation potential in vitro (Supplemental Figure 14, G and H), further confirming that SSC dysfunction in *Slit2^th^* mice was extrinsic to SSCs and therefore represented a niche effect. Second, this demonstrated that the impact of sympathetic niche dysfunction on SSCs was reversible when SSCs were introduced to a new niche environment. Notably, bone organoids in the kidney capsule were not innervated (Figure 4G), and SSC-driven osteogenesis was accordingly not altered in the kidney capsule of *Slit2^th^* hosts compared with *Slit2^fl/fl^* hosts (Supplemental Figure 15, A and B). This confirmed that the altered niche effect was local and excluded confounding systemic effects in *Slit2^th^* hosts not dependent on local sympathetic innervation.

### FSTL1 derived from sympathetic nerves suppress SSC self-renewal and osteogenesis.

**Figure 5:**
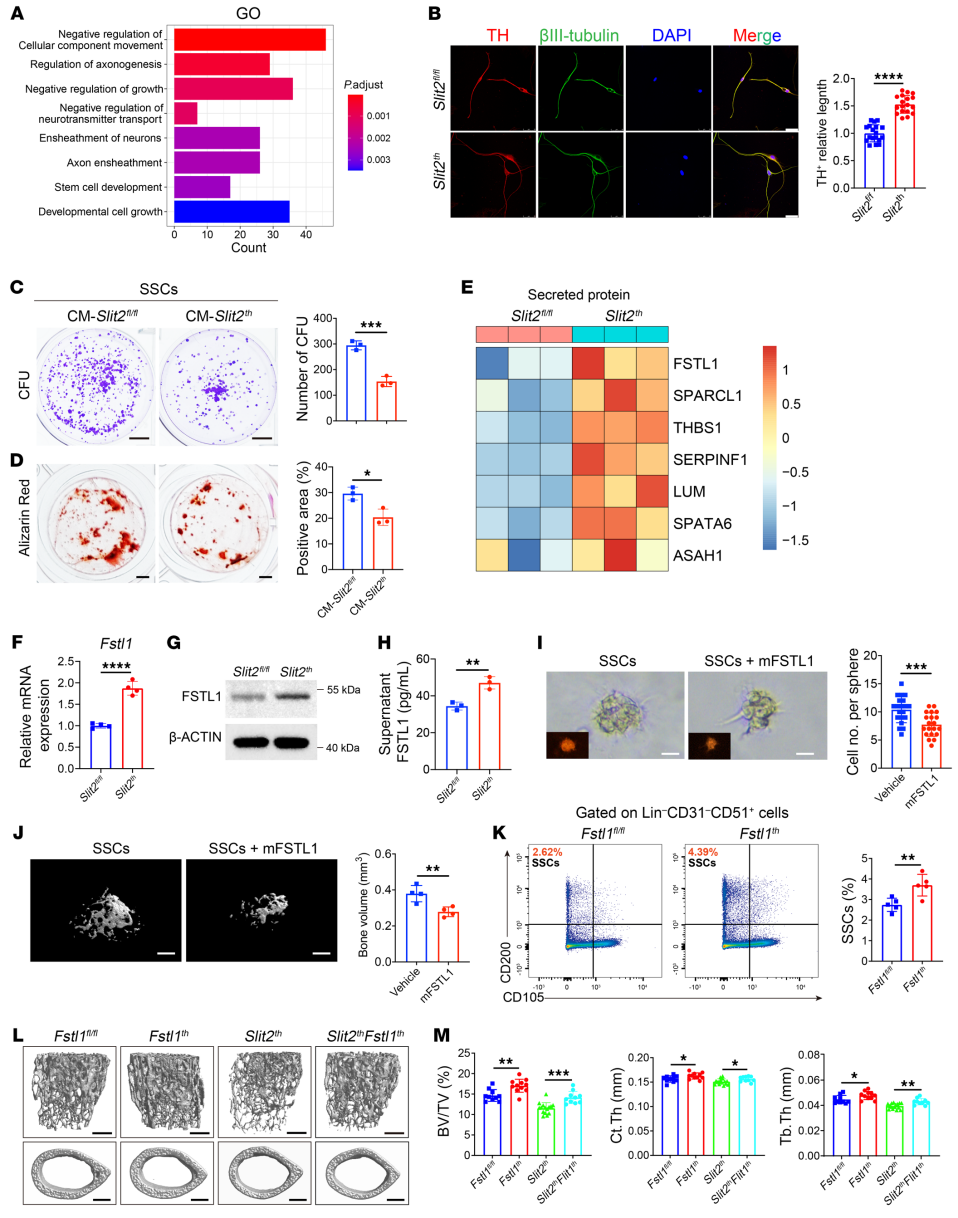
FSTL1 secreted from sympathetic nerves inhibits SSC self-renewal and osteogenesis. (**A**) GO enrichment analysis of genes differentially expressed in *Slit2^th^* sympathetic neurons relative to *Slit2^fl/fl^* sympathetic neurons. The significance values are based on a hypergeometric test. (**B**) Sympathetic neuron culture, TH^+^ (red) and βIII-tubulin^+^ (green) axon staining, and quantitative analysis in 3-week-old mice. *Slit2^fl/fl^*, *n* = 18; *Slit2^th^*. *n* = 18. Scale bars: 25 μm. (**C**) Crystal violet staining and quantitative analysis of CFU formation in SSCs stimulated with sympathetic neuronal conditioned medium. *n* = 3 per group. CM-*Slit2^fl/fl^*, conditioned culture medium of sympathetic neurons in *Slit2^fl/fl^* mice; CM-*Slit2^th^*, conditioned culture medium of sympathetic neurons in *Slit2^th^* mice. Scale bars: 5 mm. (**D**) Alizarin red staining and quantitative analysis of SSC mineralization activity after stimulation with conditioned medium from sympathetic neurons. *n* = 3 per group. Scale bars: 1 mm. (**E**) Expression of the soluble factors elevated in primary *Slit2^th^* sympathetic neurons relative to *Slit2^fl/fl^* sympathetic neurons. (**F**) mRNA levels of *Fstl1* in primary *Slit2^fl/fl^* and *Slit2^th^* sympathetic neurons were analyzed by real-time PCR. *n* = 4 per group. (**G**) Protein levels of FSLT1 in primary *Slit2^fl/fl^* and *Slit2^th^* sympathetic neurons were analyzed by immunoblotting. *n* = 3 per group. (**H**) Results from ELISA for FSTL1 secretion in primary *Slit2^fl/fl^* and *Slit2^th^* sympathetic neurons. *n* = 3 per group. (**I**) Pellet formation assay and quantitative analysis of SSCs cultured for 8 days after treatment with recombinant FSTL1 or vehicle. *n* = 4 per group. Scale bars: 20 μm. (**J**) Representative μCT images and quantitative analysis of the bone volume of bone organoids 4 weeks after renal capsule transplantation of SSCs encapsulated in Matrigel and stimulated by treatment with recombinant protein FSTL1 or vehicle. *n* = 4 per group. Scale bars: 500 μm. (**K**) Representative flow cytometry plots and relative frequency of SSCs from femurs of 3-week-old *Fstl1^fl/fl^* and *Fstl1^th^* male mice. *n* = 5 per group. (**L** and **M**) Representative μCT images of trabecular bone in the distal femur (**L**) and relative quantitative analysis of bone volume/total volume (BV/TV) and relative quantitative analysis of bone parameters (**M**) in *Fstl1^fl/fl^*, *Fstl1^th^*, *Slit2^th^*, and *Slit2^th^Fstl1^th^* male mice at 8 weeks of age. *Fstl1^fl/fl^*, *n* = 10; *Fstl1^th^*, *n* = 11; *Slit2^th^*, *n* = 15; *Slit2^th^Fstl1^th^*, *n* = 10. Scale bars: 500 μm. Error bars indicate mean ± SEM. *P < 0.05, **P < 0.01, ***P < 0.001, ****P < 0.0001, by unpaired, 2-tailed Student’s *t* test for 2-group comparisons (**B**–**D**, **F**, and **H**–**K**). *P < 0.05, **P < 0.01, ***P < 0.001, by 1-way ANOVA followed by Tukey’s post hoc test for multiple comparisons (**M**).

To investigate how sympathetic hyperinnervation resulting from *Slit2* deficiency affects SSCs and osteogenesis, we cultured primary sympathetic neurons isolated from *Slit2^th^* and *Slit2^fl/fl^* mice for RNA-Seq analysis. This identified differential expression of a transcriptional program regulating axonogenesis (Figure 5A). Consistent with this and the overall nerve phenotype in vivo, a neurite outgrowth assay showed that *Slit2* deletion remarkably enhanced axon growth of sympathetic neurons labeled by TH staining in vitro (Figure 5B). In parallel with this, conditioned medium harvested from *Slit2^th^* sympathetic neurons displayed a reduced ability to induce SSCs colony formation and mineralization, suggesting that the relevant mediator impacting SSCs was a soluble nerve-derived factor (Figure 5, C and D).

Catecholamines, including norepinephrine and epinephrine, are released in part by the postganglionic fibers of the sympathetic nervous system and influence bone homeostasis through adrenergic signaling (43, 44). To ascertain whether norepinephrine and epinephrine are implicated in the effect of SLIT2 to regulate SSC-driven osteogenesis, we mapped the expression pattern of adrenergic receptors in SSCs and identified that SSCs only express *Adrb2* (Supplemental Figure 16A) (49). Subsequently, flow cytometry analysis found no alteration in SSC numbers in *Adrb2*-knockout (*Adrb2^–/–^*) mice (Supplemental Figure 16B). We then administrated a β-adrenergic receptor agonist (salbutamol) (52) or antagonist (propranolol) (43) to *Slit2^th^* and *Slit2^fl/fl^* mice and found that adrenergic signaling was dispensable for SSC maintenance in either *Slit2^th^* or *Slit2^fl/fl^* mice (Supplemental Figure 16, C–E). Furthermore, the circulating levels of epinephrine and norepinephrine were not altered in *Slit2^th^* and *Slit2^syn1^* mice, further indicating that systemic sympathetic tone associated with catecholamines release and Slit2-regulated peripheral sympathetic innervation were independent (Supplemental Figure 16, F and G). Thus, the effect of SLIT2-regulated sympathetic nerves to control SSCs was independent of norepinephrine or epinephrine signaling and therefore distinct from known mechanisms of sympathetic regulation of bone mass.

Next, we sought to identify the molecular mediator of the effect of sympathetic nerves on the SSC niche. To this end, we performed RNA-Seq on sympathetic nerve ganglions to identify secreted factors regulated by SLIT2 (Figure 5E). Among these, FSTL1, a secreted extracellular glycoprotein recently suggested to modulate bone morphogenetic protein (BMP) signaling (53), showed substantially greater levels in *Slit2^th^* sympathetic neurons relative to *Slit2^fl/fl^* controls (Figure 5E). To verify this, experiments with 3 complementary approaches, including real-time PCR, Western-blotting, and ELISA, were performed, and all confirmed increased *Fstl1* expression in sympathetic neurons lacking *Slit2* (Figure 5, F–H).

To determine whether FSTL1 regulates SSCs, we performed a series of in vitro and in vivo assays. In vitro, recombinant FSTL1 impaired SSC colony-forming capability, serial mesensphere formation and osteogenic differentiation, where serial mesensphere formation is an established proxy for in vivo SSC self-renewal (5, 29, 54) (Figure 5I and Supplemental Figure 17, A and B). In vivo, we transplanted the same number of sorted murine SSCs mixed with Matrigel containing recombinant FSTL1 or vehicle into the kidney capsule for a bone organoid formation assay. As in the in vitro assay, recombinant FSTL1 suppressed the osteogenic activity of SSCs, as indicated by a reduction in mineralized bone volume in FSTL1-treated organoids (Figure 5J).

To further validate the importance of sympathetic FSTL1 in regulation of SSCs in vivo, we intercrossed *Fstl1^fl/fl^* mice with Th-Cre mice to specifically delete *Fstl1* expression in sympathetic neurons (*Fstl1^th^* mice). In line with FSTL1 serving as a key effector of the sympathetic SSC niche, *Fstl1^th^* mice displayed an expansion of SSCs (Figure 5K) and increased bone mass (Figure 5, L and M). Furthermore, the osteopenic phenotype observed in *Slit2^th^* mice was genetically contingent on sympathetic neuron-secreted FSTL1, as shown in *Fstl1^th^Slit2^th^* mice (Figure 5, L and M). In summary, SLIT2 expression in sympathetic neurons negatively regulated production of FSTL1, which in turn suppressed SSC self-renewal and osteogenic capacity. Thus, sympathetic neurons acted via FSTL1 as a component of the SSC niche.

### Sympathetic innervation acts on SSCs to inhibit skeletal regeneration.

**Figure 6:**
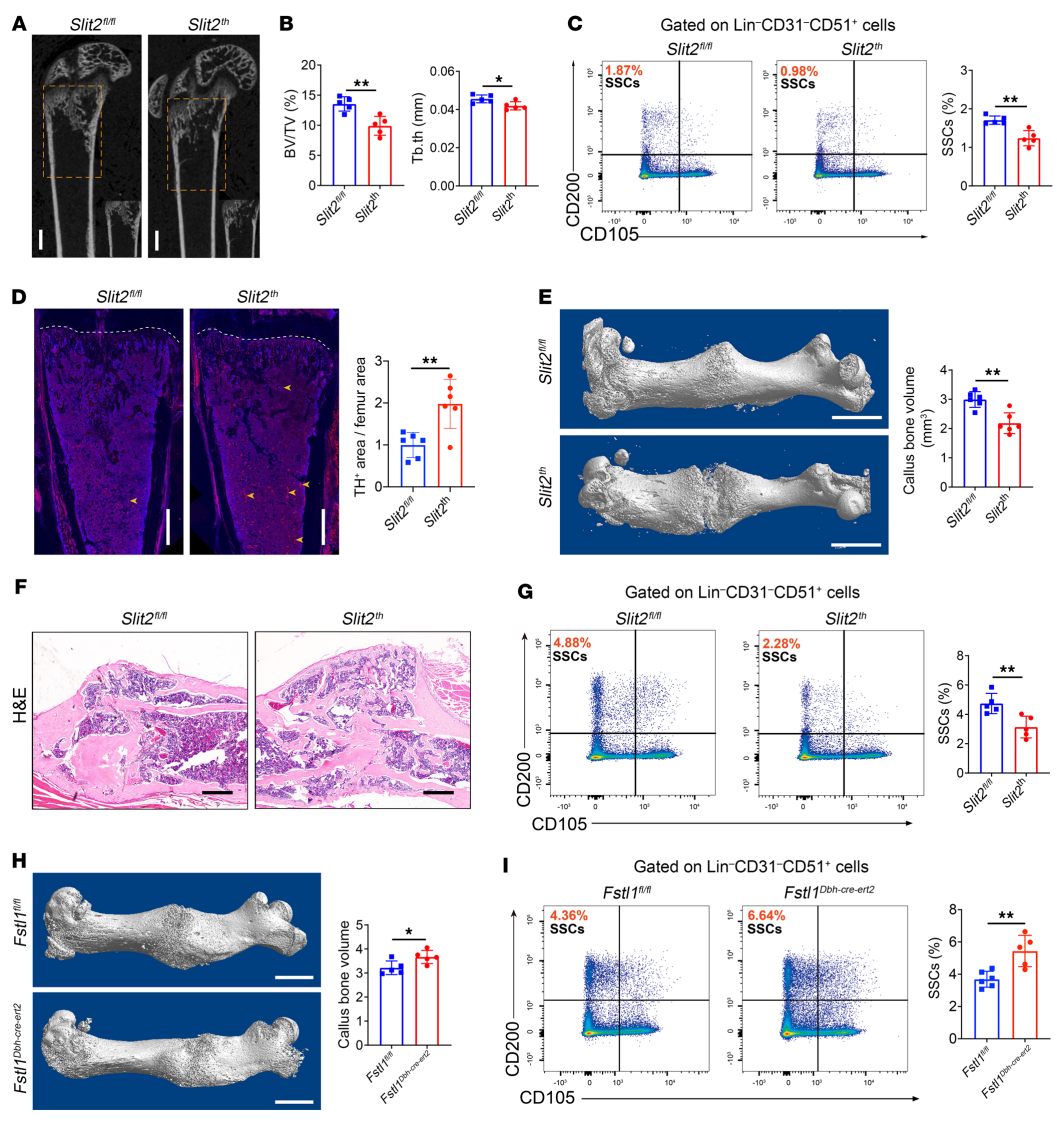
Sympathetic hyperinnervation disrupts bone regeneration by impairing SSC expansion. (**A** and **B**) Representative μCT images of bone regeneration 7 days after femoral bone marrow ablation and quantitative analysis of bone parameter in regeneration area in 6-week-old *Slit2^fl/fl^* and *Slit2^th^* male mice. *n* = 5. Scale bars: 1 mm. (**C**) Representative flow cytometry plots 7 days after femoral bone marrow ablation and relative frequency of SSCs from femurs of 6-week-old *Slit2^fl/fl^* and *Slit2^th^* male mice. *n* = 5. (**D**) Representative confocal images 7 days after femoral bone marrow ablation. Shown are TH (red) and DAPI (blue) signal and quantitative analysis of TH^+^ sympathetic nerves in femur sections. Marrow ablation was conducted in 6-week-old *Slit2^fl/fl^* and *Slit2^th^* male mice. *n* = 6; Scale bars, 500 μm. (**E**) Representative μCT images 21 days after femur fracture and quantitative analysis of callus bone volume. Fractures were performed in 6-week-old *Slit2^fl/fl^* and *Slit2^th^* male mice. *n* = 6. Scale bars: 2 mm. (**F**) H&E staining visualizing the callus formed 21 days after femur fracture in 6-week-old *Slit2^fl/fl^* and *Slit2^th^* male mice. Scale bars: 200 μm. (**G**) Representative flow cytometry plots 14 days after femur fracture and the relative frequency of SSCs from the femurs of 6-week-old *Slit2^fl/fl^* and *Slit2^th^* male mice. *n* = 5 per group. (**H**) Representative μCT images 21 days after femur fracture and quantitative analysis of bone callus volume. Fractures were performed in 6-week-old *Fstl1^fl/fl^* and *Fstl1^Dbh-cre-ert2^* male mice. *n* = 5. Scale bars: 2 mm. (**I**) Representative flow cytometry plots 14 days after femur fracture and relative frequency of SSCs from femurs of 6-week-old *Fstl1^fl/fl^* and *Fstl1^Dbh-cre-ert2^* male mice. *Fstl1^fl/fl^*, *n* = 6; *Fstl1^Dbh-cre-ert2^*, *n* = 5. Error bars indicate mean ± SEM. *P < 0.05, **P < 0.01, by unpaired, 2-tailed Student’s *t* test for 2-group comparisons.

Bone repair is mediated by SSC-based osteogenesis. It is likely that the marked expansion of SSCs occurring to mediate this skeletal repair is dependent on niche remodeling; however, this has yet to be investigated (5). To examine whether SLIT2-mediated sympathetic innervation affects bone regeneration, we first conducted a femoral bone marrow ablation study using *Slit2^th^* and *Slit2^fl/fl^* mice. μCT analysis showed that the volume of newly synthesized bone after bone marrow ablation was significantly reduced in *Slit2^th^* mice relative to *Slit2^fl/fl^* controls (Figure 6, A and B, and Supplemental Figure 18A). Similar to what was observed in uninjured mice, SSC numbers were reduced in *Slit2^th^* mice undergoing marrow osteogenic repair in parallel with increases in TH-positive sympathetic nerve fibers present around the ablation area (Figure 6, C and D, and Supplemental Figure 18B).

To confirm the role of sympathetic nerves on SSC activation and reparative capacity, we created an open femoral mid-shaft fracture model in *Slit2^th^* and *Slit2^fl/fl^* mice. Three weeks later, μCT analysis combined with histology showed that bone fracture healing was impaired in *Slit2^th^* mice (Figure 6, E and F). Consistent with the SSC phenotype seen in the bone marrow ablation model, the number of SSCs in the fracture callus was markedly reduced in *Slit2^th^* mice relative to *Slit2^fl/fl^* controls (Figure 6G and Supplemental Figure 18C). To further verify whether sympathetic neuron–derived FSTL1 plays a pivotal role in the remodeling of the SSC niche occurring in response to injury, we intercrossed *Fstl1^fl/fl^* mice with Dbh-Cre-ert2 mice to conduct inducible deletion of *Fstl1* in sympathetic neurons shortly before femoral fracture. μCT analysis revealed that fracture healing in *Fstl1^Dbh-cre-ert2^* mice, compared with *Fstl1^fl/fl^* mice, was significantly accelerated (Figure 6H). Consistent with this, an increase in SSC expansion was observed at the fracture site in *Fstl1^Dbh-cre-ert2^* mice (Figure 6I). Thus, in addition to functioning as an SSC niche component during baseline physiology, the sympathetic nerve SLIT2/FSTL1 axis also regulated dynamic remodeling of the SSC niche in response to injury. Thus, we identified what we believe to be a new sympathetic nerve SSC niche as a component governing osteogenesis during the course of both normal skeletal mineralization and regeneration.

## Discussion

Bone growth and regeneration are determined by both the intrinsic osteogenic capacity of skeletal cells and their supporting microenvironment. It is now well appreciated that a series of SSCs self-renew and sit at the apex of the differentiation hierarchy, thereby playing a central role in osteogenesis (5, 49, 55, 56). The molecular and structural components of niche-supporting tissue-specific stem cells are well studied for hematopoietic stem cells but largely unknown in bone, mostly because SSCs were only identified within the past decade, with additional SSC subsets being very recently identified. SSC identification is a prerequisite for niche component identification. Herein, we took the advantage of SLIT2-deficient mice displaying sympathetic hyperinnervation of the skeleton and identified that sympathetic nerves were a negative regulatory component of SSC niche. Consistent with this, the density of sympathetic innervation gradually increased in parallel with decreases in SSC abundance during bone growth. In contrast, both pharmacological and surgical sympathectomy promoted in vivo SSC expansion. Mechanistically, SLIT2 regulated FSTL1 secretion from sympathetic neurons, and FSTL1 in turn dampened SSC self-renewal and differentiation both in vivo and in vitro. Thus, SLIT2 and FSTL1 formed a signaling axis in sympathetic neurons that constituted what we believe to be a new component of the SSC niche. To our knowledge, this study provides the first example demonstrating the existence and physiologic importance of an SSC niche component. Given that sympathetic innervation of bone increases and remodels with age (57) and that SSC function and bone regenerative capacity decline over time (48, 50), our findings suggest that distinct sympathetic signaling mechanisms exert temporally distinct effects on skeletal biology. We propose that during early life, the SLIT2/FSTL1 axis locally modulates sympathetic innervation and restrains SSC expansion. In contrast, with aging, classical adrenergic pathways involving norepinephrine and β-adrenergic receptors become more prominent and act to suppress bone formation, contributing to age-associated bone loss and impaired skeletal homeostasis (43, 58, 59). Further direct study will be needed to confirm this proposed temporal specificity.

Axon guidance cues enable nerve fibers to reach their target organs, including bone, via autocrine or paracrine activity (10, 60, 61). Both osteoblasts and osteoclasts produce a distinct secretome of axon guidance cues (62). Interestingly, osteoblastic lineage cells locally secrete many axon-repulsive factors such as Sema3a, which reduces axonal networks in the DRG (63); whereas the osteoclast-derived axon-attractive molecule Netrin-1 stimulates sensory innervation, contributing to articular and discogenic pain (64, 65). Given this, osteoblasts and osteoclasts likely collaborate to maintain the local balance between skeletal sympathetic and sensory innervation. Among these, SLIT proteins are a soluble chemorepellent regulating innervation. This and our prior studies demonstrated that both SLIT2 and SLIT3 are predominantly expressed in osteoblasts but not osteoclasts (15, 16). Indeed, dual deletion of *Slit2* and *Slit3* in osteoblasts also leads to sympathetic hyperinnervation in skeleton as well as bone loss. Furthermore, an increasing body of studies have demonstrated that SLITs play additional roles beyond the nervous system, such as angiogenesis (14, 22). Osteoblast-derived SLIT3 promotes bone formation through its angiogenic activity; recombinant SLIT3 administration not only enhances bone fracture healing but also ameliorates bone loss (14). Consequently, as terminally differentiated daughter cells, osteoblasts may also influence the microenvironmental regulation of their associated SSCs through secretion of factors such as SLIT2-3.

Similar to the way sensory neurons produce factors such as CGRP that regulate bone formation, sympathetic neurons secrete SLIT2, which not only controls sympathetic innervation in skeleton but also intrinsically regulates the secretome of sympathetic neurons. We identified a secreted glycoprotein termed FSTL1 that is negatively regulated by SLIT2 in sympathetic neurons. FSTL1 plays multiple roles in many diseases and is essential for embryogenesis, including bone development (53, 66). Mechanistically, FSTL1 directly interacts with BMP4, a known osteogenic factor, as a signaling antagonist (53). This is consistent with a body of literature implicating BMPs as critical for governing the phenotype and maintenance of early skeletal progenitors (4, 67). More recently, a spatial transcriptomic study revealed that sensory nerve–derived FSTL1 suppresses osteogenesis in cranial bones through modulation of BMP/TGF-β signaling (68). Similarly, we here further discovered that sympathetic nerve–derived FSTL1 suppressed bone formation in long bones by targeting the SSC niche. Given that FSTL1 also promotes osteoclastogenesis through RANKL-mediated NF-κB activation and M-CSF–induced precursor proliferation (69), blocking FSTL1 stimulation may offer a therapeutic strategy to enhance SSC-driven bone formation, as well as inhibiting osteoclast-driven bone resorption.

Though FSTL1 is a key factor secreted by sympathetic nerves to regulate SSC-driven osteogenesis, we still cannot exclude that those sympathetic neurons produce other soluble molecules regulating SSCs or that there is central nervous regulation of the SSC niche. Whereas sensory nerves significantly regulate cortical bone mass (70, 71), alteration in sympathetic innervation results only in a modest cortical bone phenotype in *Slit2^th^* mice, indicating that sensory nerves, rather than sympathetic nerves, may primarily support osteogenesis driven by PSCs, though more direct evidence is required. Future lines of investigation will be needed to decipher the differences between the niches supporting various classes of SSCs, such as periosteal versus endosteal SSCs.

As an arm of the autonomic nervous system, sympathetic innervation of the skeleton has long been studied as a mechanism to suppress bone formation. As our study focused on mice that had yet to achieve full skeletal maturity, it remains to be determined whether the SLIT2/FSTL1 axis plays a comparable role in the aged skeleton. Given prior evidence that β-adrenergic signaling is a dominant regulatory mechanism in aged bones, it is plausible that SLIT2-mediated regulation of the sympathetic secretome exerts its strongest effects during the early postnatal period, while adrenergic signaling may become more prominent later in life. This putative distinction, which would require direct confirmation, highlights a temporal specificity to the neuroregulation of SSCs and underscores the need for future studies in aged models to assess whether the SSC niche undergoes functional shifts in response to changes in sympathetic tone with aging. Most studies have demonstrated that increases in sympathetic tone inhibit osteoblastogenesis as well as stimulating osteoclastogenesis via a central autonomic network (42, 43). Despite ongoing controversy about how sympathetic bone innervation remodels over the course of aging (26, 57), this study shows that skeletal sympathetic innervation increased over the course of early modeling bone growth, implicating sympathetic nerve SSC niche remodeling as a potential contributing factor to progressive declines in SSC numbers both at early postnatal life and later during aging (48). Moreover, sympathetic innervation has been recently suggested to promote osteocyte-mediated bone loss during lactation or aging (72, 73). Here, in addition to targeting mature bone cells, skeletal sympathetic innervation suppressed expansion of SSCs and SSC osteogenic capacity under physiological and pathological conditions. Sensory innervation of the skeleton has also been characterized as supporting bone formation by targeting a series of bone precursor cells mediating early osteogenesis and bone regeneration (11, 68, 74). Hence, further work similar to the approach here is needed to clarify whether sensory nerves also constitute part of the SSC niche. If sensory nerves impact the SSC niche, then how the sensory and sympathetic elements of the SSC niche interact will be an intriguing question to be addressed in the future. Not only will this line of investigation likely elucidate a stem cell basis for multiple skeleton-related diseases, but it will also present potential strategies to facilitate the application of stem cell niche–targeted therapy for bone disorders.

## Methods

### Sex as a biological variable.

Our study examined male and female animals, and similar findings are reported for both sexes.

### Animal models.

C57BL/6J WT mice were purchased from Xiamen University Laboratory Animal Center. Synapsin-Cre mice, Advillin-Cre mice and Th-Cre mice were obtained from The Jackson Laboratory. *Slit2^fl/fl^* mice were obtained from the Research group of Professor Alain Chédotal (Marie Curie University, France). *Fstl1^fl/fl^* mice were obtained from GemPharmatech Co., Ltd., Jiangsu (Nanjing, China), Dbh-cre-ert2 were obtained from Jing Yang Research Group, School of Life Sciences, Peking University (Beijing, China), *Adrb2^–/–^* mice were obtained from Kairui Mao Research Group, School of Life Sciences, Xiamen University (Xiamen, China). All mice were bred and maintained in a standard pathogen-free environment of the Laboratory Animal Center in Xiamen University, and all animal experiments were approved by the Animal Care and Use Committee of Xiamen University. Unless otherwise specified, all mice used in this study, including genetically modified strains, were maintained on a C57BL/6J background.

### ELISA assay.

We analyzed Fstl1, Ne and Epi ELISA using commercial kits. The detailed catalog numbers for all ELISA kits are provided in Supplemental Table 1, and the experimental procedures were carried out according to the manufacturer’s instructions. All ELISAs were run according to the manufacturer’s instructions.

### RNA extraction and real-time PCR analysis.

Following the manufacturer’s instructions, Trizol (Invitrogen, 15596026) was used to separate total RNA from tissues or cells in culture. On a CFX Connect instrument from Bio-Rad, we carried out qPCR using SYBR Green Power PCR Master Mix (Invitrogen, A25777); Hprt amplification served as an internal control. Dissociation curve analysis was performed for every experiment. Primers used for qPCR are listed in Supplemental Table 2. Transcript levels were normalized to Hprt and fold change was calculated based on ΔCt.

### Kidney capsule transplantation of SSCs.

This study was performed as previously described (5, 49), In brief, 8-week-old mice were anesthetized with 2.5% isoflurane and placed in a lateral position on a heating pad. The fur on the right dorsal side of each mouse was shaved, and the skin was disinfected with povidone-iodine. The tip of a 20 μL pipette was gently heated over an alcohol lamp to smooth its surface. The refined tip was then used to carefully create a space along the longitudinal side of the kidney parenchyma, preparing a site for SSC implantation. Matrigel containing SSCs was thoroughly mixed, and an equal amount of the SSC-Matrigel mixture was slowly injected beneath the renal capsule on one side of each mouse using a 20 μL pipette, with approximately 1 × 10^4^ SSCs transplanted per mouse. Samples were collected and analyzed 4 weeks after the transplantation procedure.

### Bone marrow cavity transplantation of SSCs.

At approximately 8 weeks of age, *Slit2^fl/fl^*-MIP-GFP and *Slit2^th^*-MIP-GFP host mice underwent in situ bone marrow cavity transplantation of SSCs. MIP-GFP hosts were used to tolerize recipients to GFP-expressing graft cells. On the day of surgery, mice were irradiated with approximately 9 Gy using an RS-2000 irradiator (Rad Source). FACS-sorted SSCs were suspended in Matrigel (Cat#: 354230, Corning) and kept on ice. Simultaneously, donor bone marrow was extracted from femurs and tibias via PBS perfusion with a 21G needle. Red blood cells were lysed using ACK buffer (Gibco, Cat#: A1049201), and the suspension was filtered through a 40 μm strainer for intravenous injection. Host mice were anesthetized with 2.5% isoflurane, the surgical site was shaved and disinfected with povidone-iodine, and a approximately 1 mm incision was made to expose the right femur. Using an insulin needle, approximately 2 × 10^4^ SSCs in Matrigel were injected into the bone marrow cavity through the knee joint. Each mouse also received approximately 1 × 10^7^ donor marrow cells intravenously for hematopoietic reconstitution. Samples were collected and analyzed 4 weeks after transplantation.

### Bone clear: whole tissue immunofluorescence staining of femurs.

The experiment was conducted following optimization based on the previously described protocol (44), Briefly, 3-week-old mice were perfused with PBS containing 50 μg/mL heparin, followed by 1% paraformaldehyde (PFA) and 10% sucrose. Dissected femurs were fixed in 0.5% PFA with 10% sucrose at room temperature for 2 hours and overnight at 4°C, then washed and decalcified in 350 mM EDTA (pH 6.5) at 37°C for 72 hours. Samples underwent methanol dehydration, followed by permeabilization in PBS containing 0.2% Triton X-100, 0.1% sodium deoxycholate, 10% DMSO, and 25 mM EDTA. Blocking was performed in PBS with 0.2% Triton X-100, 10% DMSO, 5% donkey serum, and 25 mM EDTA. Primary antibodies were applied at 37°C for 72 hours in PBS with 0.2% Tween-20, 10 μg/mL heparin, and 5% donkey serum. After washing, tissues were incubated with Alexa Fluor–conjugated secondary antibodies under similar conditions and washed again. Optical clearing was achieved via graded methanol, dichloromethane/methanol, and 100% dibenzyl ether. Imaging was performed using Stitching Light Sheet Selective Plane Illumination Microscopy (SLS-SPIM). Imaris software (Bitplane) was used to reconstruct anatomical regions, and distances from TH^+^ sympathetic nerve fibers to selected reference points were quantified.

### Sympathetic denervation surgery.

Two-week-old WT mice were anesthetized with a minimal dose of isoflurane via inhalation and placed on a heated pad to maintain body temperature. Sterilized micro forceps and scissors were wiped with povidone-iodine and kept ready for use. Mice were positioned under a microscope for surgical procedures. The dorsal skin was gently incised using micro scissors to expose the lumbar spine. Under microscopic guidance, the sympathetic chain (L2-L5) attached to the vertebrae was carefully transected using micro scissors. The skin incision was then sutured, disinfected with povidone-iodine, and the mice were placed back on the heated pad until full recovery. Tissue samples were collected from the mice for analysis 3 weeks after surgery.

### Western blot analysis.

We carried out Western blot analysis in accordance with a previously mentioned common protocol (14). Brain tissue was ground under liquid nitrogen and then lysed in RIPA buffer containing phosphatase inhibitors and phenylmethylsulfonyl fluoride (PMSF) for 30 minutes to obtain total protein. The electronic imprinting device uses SDS-PAGE gel to isolate proteins and transfer them to PVDF membrane (Millipore). The membrane was then blocked in 5% skim milk for 1 hour, then incubated at 4°C overnight with the following primary antibodies: Slit1(1:500, Cat#sc-376756, Santa Cruz), Slit2 (1:500, Cat#sc-514499, Santa Cruz; 1:500, Proteintech, Cat#20217-1-AP), Slit3 (1:1000, Cat#AF3629; RRID: AB_2189998, R&D Systems), Fstl1(1:1000, Cat#PA5-31113; RRID: AB_2548587, Invitrogen) and β-Actin (1:20,000, Cat#66009-1-Ig; RRID: AB_2687938, Proteintech). The next day, the membrane was washed 3 times with TBS containing 0.1% Tween and incubated with fluorescent secondary antibody for 1 hour. The membrane was washed 3 times with TBST, then visualized using an image quantla-4000 imaging system (GE Healthcare, Chicago, Illinois, USA), and the outcomes were examined using ImageJ software.

### Micro-CT analysis.

Mice femur specimens were fixed with 4% FPA and scanned with high-resolution μCT. The specific scanning parameters were set as follows: voltage 60 Kv and resolution 10 mm, the pixel size is 2,016 × 1,344, and the AI value is 0.25 mm. After scanning using the Skyscan 1272 (Bruker; Aartselaar, Belgium) and the Scanco Medical μCT 35 system (Scanco Medical AG) at the Citigroup Biomedical Imaging Core with previously described parameters (75), 3-dimensional reconstruction was performed with the NRecon software, and CTAn software was used to analyze and compare the relevant parameters of reconstructed bone tissue, including percent bone volume (BV/TV), trabecular thickness (Tb.Th), trabecular number (Tb.N), trabecular separation (Tb.Sp), and cross-sectional thickness (Ct.Th).

### Osteoblasts culture and differentiation assays.

Primary bone marrow cells from 3-week-old mice or SSCs sorted from the femur and tibia of mice and cultured in α-MEM medium (Gibco) containing 10% fetal bovine serum, 1% penicillin/streptomycin, 1% HEPES, and 1% nonessential amino acids. Osteoblast differentiation was then stimulated by ascorbic acid and β-glycerophosphate. The medium was changed every 2 days. To determine ALP activity, osteoblasts were fixed with 4% PFA and stained with a solution containing fast blue and naphthol (Sigma-Aldrich). ALP activity was measured using a spectrophotometer (Thermo).

### Histomorphometry.

On days 1 and 5 prior to sacrifice of mice, we intraperitoneally injected 20 mg/kg of calcein into mice to gauge the rate of bone formation. As stated in our prior study (14), von Kossa, toluidine blue, and TRAP staining were carried out using resin embedding and sectioning without decalcification. Then, as previously mentioned (14), we performed histomorphometric analysis using the Osteomeasure System (OsteoMetrics, Atlanta, USA).

### FACS and cell sorting.

FACS to isolate SSCs and endothelial cells was modified from previously reported methods (14, 49). Briefly, mice femurs and tibia were isolated and crushed in Hank’s buffer containing 10 mmol·L^−1^ HEPES. Digestion buffer was freshly prepared with 2.5 mg·mL^−1^ collagenase A and 1 U·mL^−1^ dispase II, and the mixture was gently stirred at 37°C for 15 minutes. Next, digestion was terminated with a FACS buffer containing 2% fetal bovine serum and 2 mmol·L^−1^ EDTA (pH 8.0), and the digested cells were centrifuged at 4°C and 1,500 rpm for 5 minutes. The centrifuged cells were washed twice with FACS buffer, then treated with anti-mouse CD16/CD32 antibodies (Thermo Fisher Scientific) for 15 minutes to block nonspecific staining, and incubated for 30 minutes with the following antibodies: CD45-BUV395, Ter119-BUV395, 6c3-Percp-cy5.5, CD90.2-Percp-cy5.5, CD31-FITC, EMCN-APC, Embigin-APC, CD51-PE, CD105-Pe-cy7, CD200-BV421. Dead cells staining positive with DAPI were excluded from the analyses. FACS analysis for protein expression of each cell marker was performed on a BD Fortessa X20 machine and analyzed with FlowJo V10 software. FACS sorting for SSCs from 3-week-old mouse femurs and tibias was performed using the MoFlo Astrios EQS, BD FACSAria Fusion, and BD FACSAria III flow cytometers.

### CFU-F forming assay.

For CFU-F determination, single FACS sorted SSCs were inoculated into 6-well plates at a ratio of 1,000 cells per well and allowed to form individual colonies. Cells incubated at 37°C with 5% CO_2_, with the medium changed every 3 days. The number of CFU-F after staining with 1% crystal violet was scored on day 8 by light microscopy.

### In vitro spheroid formation assays.

For spheroid formation assay, FACS isolated single cells were plated at a density of 100 cells/cm^2^ and allowed to grow in ultra-low adherence culture dishes. Plates were incubated at 37°C supplied with 5% CO2 and left undisturbed for a week. Half of the media was replaced every 7 days. Spheroid formation were dissociated into single cells using Accutase solution (Gibco) and were subsequently re-plated to generate secondary and tertiary mesenspheres.

### Immunofluorescence and confocal imaging.

Mouse femurs were isolated and immediately fixed overnight with ice-cold 4% paraformaldehyde solution. For femur samples from 3-week-old mice, these were decalcified with 0.5 M EDTA (pH 8.0) at 4°C for 7 days. All femur samples were cut into 35 μm thick sagittal sections using a cryostat (Leica) and embedded in OCT compound (Sakura). Sections were blocked with 5% donkey serum at room temperature for 30 minutes after treatment with 0.2% Triton X-100 for 10 minutes and incubated overnight at 4°C with antibodies. The primary antibodies used in this study can be seen in the Supplemental Table 1. Nuclei were counterstained with 4′,6-diamidino-2-phenylindole (DAPI). Images of the samples were taken using a Leica TCS SP8 DLS confocal microscope. lmages were analyzed with lmaris software (Bitplane) or ImageJ.

### Sympathetic neuron extraction, culture, and immunofluorescence.

Three-week-old *Slit2^fl/fl^* and *Slit2^th^* male mice were sacrificed by cervical dislocation and then immersed in 75% alcohol for 3 minutes. The backs of the mice were dissected and isolated, placed in 10-cm dishes containing Hank’s solution, and placed on ice. Mouse dorsal root neurons were removed by dissection under a microscope and placed in Eppendorf tubes that also had 0.125% collagenase type I for 90 minutes at 37°C and 200 rpm with shaking. Then the supernatant was discarded after centrifugation (4°C, 1,400 rpm, 5 minutes) and replaced with an equal amount of Hank’s solution for neutralization. Finally, specimens were placed in an adequate volume of 0.25% trypsin to cover the tissue and shaken at 37°C for about 20 minutes. The digestion was terminated and the seed plates were counted after resuspension with DMEM medium containing 10% fetal bovine serum filtered through a 40 μm sieve. The culture was incubated overnight and then replaced with neuronal medium containing B27 for 4–5 days until the sympathetic neuron axons was extended, immunofluorescent TH and βIII-Tubulin were stained on neuron axons. Imaging was performed using a confocal microscope Leica TCS SP8 DLS. Use ImageJ to calculate the relative extension length of TH+ or βIII-Tubulin of the neuronal axon,the length is defined as the longest distance from the neuronal soma to the distal end of the axon.

### Bone fracture model.

The bone fracture procedure was as previously reported (14). In brief, the mice were anesthetized with 2.5% isoflurane inhalation, shaved the hair at the surgical site, sterilized with iodophor, and the entire femur was surgically exposed. The femur then underwent a midshaft osteotomy using a Dremel saw with a diamond thin cutting wheel. Through the knee, one 25G needle was used to pierce the bone marrow. To stabilize the fracture, a stainless-steel wire with a diameter of less than 0.2 mm was then inserted into the femur’s intramedullary canal. The skin was then closed with absorbable sutures, and the closed wound was disinfected with iodophor. Experimental mice were euthanized 3 weeks after fracture surgery and analyzed.

### Tamoxifen-induced lineage tracing and fracture model.

To examine the role of sympathetic neuron-derived FSTL1 in SSC regulation during bone repair, *Dbh-CreERT2; Fstl1^fl/fl^* mice were used. Tamoxifen (Sigma, T5648) was administered intraperitoneally at a dose of 75 mg/kg daily for 5 consecutive days beginning at 4 weeks of age to induce Cre-mediated recombination. At 6 weeks of age, a standardized transverse femoral fracture was performed under anesthesia and stabilized using an intramedullary pin. Femurs were collected at 8 weeks of age (2 weeks after fracture) for flow cytometry analysis, and at 9 weeks of age (3 weeks after fracture) for micro-CT assessment.

### RNA-Seq.

Sympathetic chains from 3-week-old *Slit2^fl/fl^* and *Slit2^th^* male mice were isolated as described above and cultured in neural medium containing B27 for 4–5 days (37°C, 5% CO_2_), after which cells were digested and sent for RNA sequencing as previously described (14). Briefly, cDNA libraries were prepared using the Illumina TruSeq RNA Sample Preparation Kit and sequenced on an Illumina HiSeq 4000. Reads were aligned to the mm10 reference genome using HISAT2 (v2.2.1). Differential expression analysis was performed with edgeR (v3.36.0, Bioconductor), excluding genes with expression <1 CPM. Heatmaps and volcano plots were generated using pheatmap (v1.0.12) and ggplot2 (v3.3.6), respectively. Functional enrichment of DEGs was conducted using clusterProfiler (v4.4.4), with Benjamini-Hochberg adjustment and a *P* value cutoff of 0.05. Gene set enrichment analysis (GSEA) was performed with GSEA software (v4.3.2, Linux), using ontology and curated gene sets from MSigDB (v2022.1).

### Statistics.

The software GraphPad Prism (v6.0a; GraphPad, La Jolla, CA, USA) was used for all statistical analysis. For a comparison of just 2 groups, a 2-tailed Student’s *t* test was used to determine significance. Tukey’s post hoc tests were used to evaluate the significance of comparisons made between multiple groups using 1-way ANOVA with appropriate post hoc tests for multiple-group comparisons. Two-way analysis of variance was used for different factors and their interactions, which was implemented using R version 4.5.0. An indication of statistical significance was a *P* value 0.05 (*P < 0.05, **P < 0.01, ***P < 0.001, ****P < 0.0001). Error bars are shown as mean ± SEM.

### Study approval.

All animal experiments were approved by the Institutional Animal Care and Use Committee of Xiamen University and were conducted in accordance with institutional guidelines.

### Data availability.

RNA-seq data have been deposited the NCBI’s Gene Expression Omnibus database (GEO GSE284991). All data values reported in this work are reported in the Supporting Data Values file. The article and the supplemental material present all data needed to evaluate the conclusions.

## Author contributions

RX supervised this project. RX MBG and WNC designed the research and wrote the manuscript. ZXW, NL, ZQL, ZHC, XMH, JH, XXL, FS, YL, DCZ, and BHS performed research. ZXW, NL, XMH, and SF assisted flow cytometry and FACS. DWZ, LFC, and HTH completed whole tissue staining research. ZXW, ZQL, and ZHC supported animal studies and management. ZXW, XW, LM, and JH analyzed data.

## Supplementary Material

Supplemental data

Unedited blot and gel images

Supporting data values

## Version 1 10/15/2025

Electronic publication

## References

[1] Fuchs E, Blau HM (2020). Tissue stem cells: architects of their niches. Cell Stem Cell.

[2] Comazzetto S (2021). Niches that regulate stem cells and hematopoiesis in adult bone marrow. Dev Cell.

[3] Lane SW (2014). Modulating the stem cell niche for tissue regeneration. Nat Biotechnol.

[4] Chan CK (2015). Identification and specification of the mouse skeletal stem cell. Cell.

[5] Debnath S (2018). Discovery of a periosteal stem cell mediating intramembranous bone formation. Nature.

[6] Sun Q (2023). Dedifferentiation maintains melanocyte stem cells in a dynamic niche. Nature.

[7] Tevlin R (2017). Pharmacological rescue of diabetic skeletal stem cell niches. Sci Transl Med.

[8] Kurenkova AD (2020). Niches for skeletal stem cells of mesenchymal origin. Front Cell Dev Biol.

[9] Cheng W (2024). Skeletal stem-cell therapy: challenge meets opportunity. Lancet.

[10] Fukuda T (2013). Sema3A regulates bone-mass accrual through sensory innervations. Nature.

[11] Hu B (2020). Sensory nerves regulate mesenchymal stromal cell lineage commitment by tuning sympathetic tones. J Clin Invest.

[12] Zhang Y (2016). Implant-derived magnesium induces local neuronal production of CGRP to improve bone-fracture healing in rats. Nat Med.

[13] Watson EC, Adams RH (2018). Biology of bone: the vasculature of the skeletal system. Cold Spring Harb Perspect Med.

[14] Xu R (2018). Targeting skeletal endothelium to ameliorate bone loss. Nat Med.

[15] Li N (2020). Osteoclasts are not a source of SLIT3. Bone Res.

[16] Li Z (2024). Bone controls browning of white adipose tissue and protects from diet-induced obesity through Schnurri-3-regulated SLIT2 secretion. Nat Commun.

[17] Brose K (1999). Slit proteins bind Robo receptors and have an evolutionarily conserved role in repulsive axon guidance. Cell.

[18] Jaworski A, Tessier-Lavigne M (2012). Autocrine/juxtaparacrine regulation of axon fasciculation by Slit-Robo signaling. Nat Neurosci.

[19] Wang SZ (2013). Slit/Robo signaling mediates spatial positioning of spiral ganglion neurons during development of cochlear innervation. J Neurosci.

[20] Niclou SP (2000). Slit2 is a repellent for retinal ganglion cell axons. J Neurosci.

[21] Kim M (2019). Slit/Robo signals prevent spinal motor neuron emigration by organizing the spinal cord basement membrane. Dev Biol.

[22] Rama N (2015). Slit2 signaling through Robo1 and Robo2 is required for retinal neovascularization. Nat Med.

[23] Svensson KJ (2016). A secreted Slit2 fragment regulates adipose tissue thermogenesis and metabolic function. Cell Metab.

[24] Tavora B (2020). Tumoural activation of TLR3-SLIT2 axis in endothelium drives metastasis. Nature.

[25] Logan M (2002). Expression of Cre Recombinase in the developing mouse limb bud driven by a Prxl enhancer. Genesis.

[26] Maryanovich M (2018). Adrenergic nerve degeneration in bone marrow drives aging of the hematopoietic stem cell niche. Nat Med.

[27] Zhang B (2020). Hyperactivation of sympathetic nerves drives depletion of melanocyte stem cells. Nature.

[28] Arthur A (2011). EphB/ephrin-B interactions mediate human MSC attachment, migration and osteochondral differentiation. Bone.

[29] Méndez-Ferrer S (2010). Mesenchymal and haematopoietic stem cells form a unique bone marrow niche. Nature.

[30] Abeynayake N (2021). Crosstalk between skeletal and neural tissues is critical for skeletal health. Bone.

[31] Xie M (2019). Schwann cell precursors contribute to skeletal formation during embryonic development in mice and zebrafish. Proc Natl Acad Sci U S A.

[32] Kaucka M (2018). Signals from the brain and olfactory epithelium control shaping of the mammalian nasal capsule cartilage. Elife.

[33] Arranz L (2014). Neuropathy of haematopoietic stem cell niche is essential for myeloproliferative neoplasms. Nature.

[34] Lucas D (2013). Chemotherapy-induced bone marrow nerve injury impairs hematopoietic regeneration. Nat Med.

[35] Roshanzamir S (2016). Autonomic dysfunction and osteoporosis after electrical burn. Burns.

[36] Sweetapple HA (1946). Sudeck’s atrophy. Med J Aust.

[37] Tangella AV (2023). Imaging modalities and their findings in patients with complex regional pain syndrome: a review. Cureus.

[38] Brazill JM (2019). Nerves in bone: evolving concepts in pain and anabolism. J Bone Miner Res.

[39] Lorenz MR (2021). A neuroskeletal atlas: spatial mapping and contextualization of axon subtypes innervating the long bones of C3H and B6 mice. J Bone Miner Res.

[40] Meyers CA (2020). A neurotrophic mechanism directs sensory nerve transit in cranial bone. Cell Rep.

[41] Tomlinson RE (2016). NGF-TrkA signaling by sensory nerves coordinates the vascularization and ossification of developing endochondral bone. Cell Rep.

[42] Elefteriou F (2005). Leptin regulation of bone resorption by the sympathetic nervous system and CART. Nature.

[43] Takeda S (2002). Leptin regulates bone formation via the sympathetic nervous system. Cell.

[44] Ding X (2019). Panicle-shaped sympathetic architecture in the spleen parenchyma modulates antibacterial innate immunity. Cell Rep.

[45] Orefice LL (2019). Targeting peripheral somatosensory neurons to improve tactile-related phenotypes in ASD models. Cell.

[46] Yang G (2024). Identification of the metaphyseal skeletal stem cell building trabecular bone. Sci Adv.

[47] Matsushita Y (2023). Bone marrow endosteal stem cells dictate active osteogenesis and aggressive tumorigenesis. Nat Commun.

[48] Ambrosi TH (2021). Aged skeletal stem cells generate an inflammatory degenerative niche. Nature.

[49] Sun J (2023). A vertebral skeletal stem cell lineage driving metastasis. Nature.

[50] Ambrosi TH (2021). Distinct skeletal stem cell types orchestrate long bone skeletogenesis. Elife.

[51] Yamazaki S (2011). Nonmyelinating Schwann cells maintain hematopoietic stem cell hibernation in the bone marrow niche. Cell.

[52] Straat ME (2023). Stimulation of the beta-2-adrenergic receptor with salbutamol activates human brown adipose tissue. Cell Rep Med.

[53] Geng Y (2011). Follistatin-like 1 (Fstl1) is a bone morphogenetic protein (BMP) 4 signaling antagonist in controlling mouse lung development. Proc Natl Acad Sci U S A.

[54] Bok S (2023). A multi-stem cell basis for craniosynostosis and calvarial mineralization. Nature.

[55] Chan CKF (2018). Identification of the human skeletal stem cell. Cell.

[56] Yuan G (2024). Skeletal stem cells in bone development, homeostasis, and disease. Protein Cell.

[57] Ho YH (2019). Remodeling of bone marrow hematopoietic stem cell niches promotes myeloid cell expansion during premature or physiological aging. Cell Stem Cell.

[58] Khosla S (2018). Sympathetic β1-adrenergic signaling contributes to regulation of human bone metabolism. J Clin Invest.

[59] Luo N (2022). A neuronal action of sirtuin 1 suppresses bone mass in young and aging mice. J Clin Invest.

[60] Makita T (2008). Endothelins are vascular-derived axonal guidance cues for developing sympathetic neurons. Nature.

[61] Chédotal A (2019). Roles of axon guidance molecules in neuronal wiring in the developing spinal cord. Nat Rev Neurosci.

[62] Negishi-Koga T (2011). Suppression of bone formation by osteoclastic expression of semaphorin 4D. Nat Med.

[63] Leitão L (2020). Osteoblasts are inherently programmed to repel sensory innervation. Bone Res.

[64] Zhu S (2019). Subchondral bone osteoclasts induce sensory innervation and osteoarthritis pain. J Clin Invest.

[65] Ni S (2019). Sensory innervation in porous endplates by Netrin-1 from osteoclasts mediates PGE2-induced spinal hypersensitivity in mice. Nat Commun.

[66] Mattiotti A (2018). Follistatin-like 1 in development and human diseases. Cell Mol Life Sci.

[67] Salazar VS (2019). Reactivation of a developmental *Bmp2* signaling center is required for therapeutic control of the murine periosteal niche. Elife.

[68] Tower RJ (2021). Spatial transcriptomics reveals a role for sensory nerves in preserving cranial suture patency through modulation of BMP/TGF-β signaling. Proc Natl Acad Sci U S A.

[69] Kim HJ (2016). Follistatin-like 1 promotes osteoclast formation via RANKL-mediated NF-κB activation and M-CSF-induced precursor proliferation. Cell Signal.

[70] Tomlinson RE (2017). NGF-TrkA signaling in sensory nerves is required for skeletal adaptation to mechanical loads in mice. Proc Natl Acad Sci U S A.

[71] Chen H (2019). Prostaglandin E2 mediates sensory nerve regulation of bone homeostasis. Nat Commun.

[72] Guo Q (2023). Sympathetic innervation regulates osteocyte-mediated cortical bone resorption during lactation. Adv Sci (Weinh).

[73] Zhang Y (2021). Neuronal induction of bone-fat imbalance through osteocyte neuropeptide Y. Adv Sci (Weinh).

[74] Pei F (2023). Sensory nerve niche regulates mesenchymal stem cell homeostasis via FGF/mTOR/autophagy axis. Nat Commun.

[75] Shim JH (2013). Schnurri-3 regulates ERK downstream of WNT signaling in osteoblasts. J Clin Invest.

