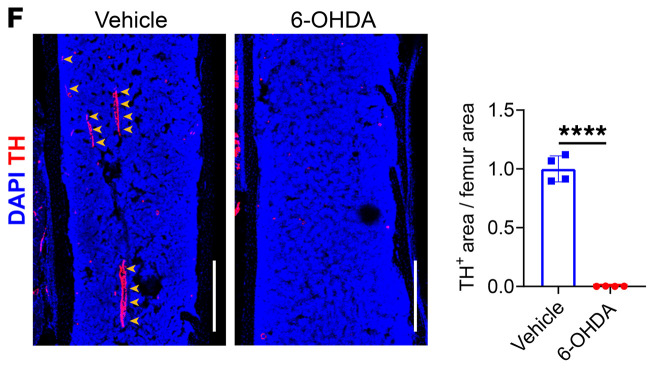# Corrigendum to Axon guidance cue SLIT2 regulates the murine skeletal stem cell niche through sympathetic innervation

**DOI:** 10.1172/JCI203674

**Published:** 2026-01-16

**Authors:** Zuoxing Wu, Na Li, Zhengqiong Luo, Zihan Chen, Xuemei He, Jie Han, Xixi Lin, Fan Shi, Haitao Huang, Baohong Shi, Yu Li, Xin Wang, Lin Meng, Dachuan Zhang, Lanfen Chen, Dawang Zhou, Weinan Cheng, Matthew B. Greenblatt, Ren Xu

Original citation: *J Clin Invest*. 2025;135(20):e193014. https://doi.org/10.1172/JCI193014

Citation for this corrigendum: *J Clin Invest*. 2026;136(2):e203674. https://doi.org/10.1172/JCI203674

Following the publication of this article, the authors identified errors in the representative images in Figures 3F and 6H as well as the Osx-Cre cortical bone image in Supplemental Figure 1E. The Editors previously issued an Expression of Concern (1) and requested institutional oversight into this matter. An institutional review by the School of Medicine at Xiamen University concluded that the errors were inadvertent and do not undermine the scientific conclusions of the study.

The correct Figures 3F and 6H, based on the original source data, are shown below, and an updated version of the supplemental material has been provided. The HTML and PDF versions of the article have been updated online.

The authors regret the errors.

## Supplementary Material

Supplemental data

## Figures and Tables

**Figure 6H:**
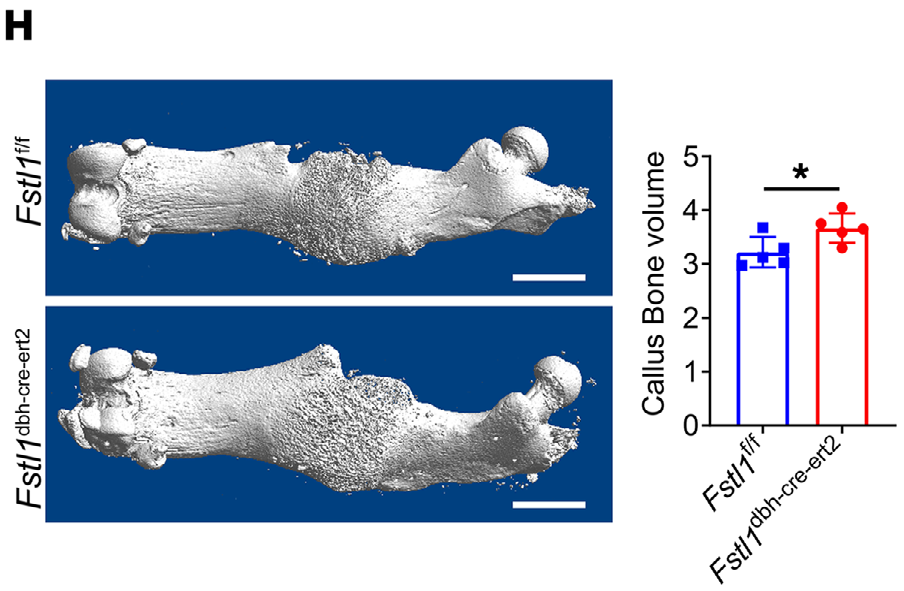


**Figure 3F:**