# Supplementary material for: Axon guidance cue SLIT2 regulates the murine skeletal stem cell niche through sympathetic innervation
Source: J Clin Invest. 2025 Oct 15;135(20):e193014. doi: 10.1172/JCI193014 (PMC12520678; doi:10.1172/JCI193014)
Supplement: Unedited blot and gel images [file jci-135-193014-s302.pdf]

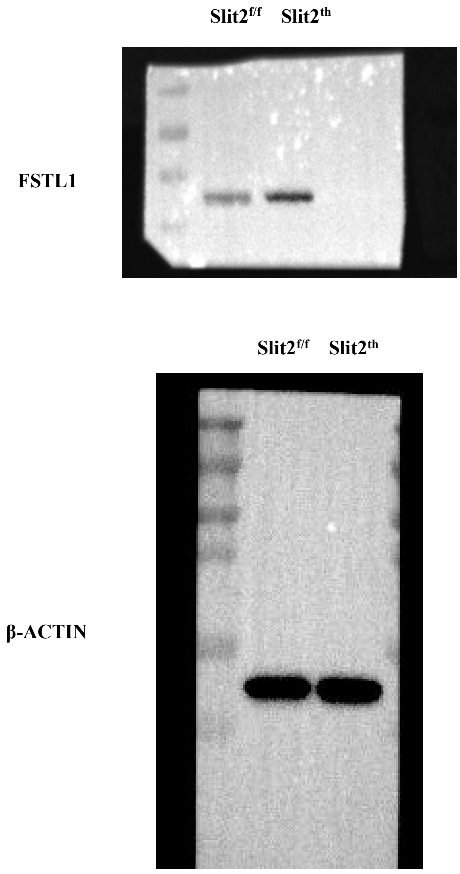

Figure 5G

Brain OBs OCs

SLIT2

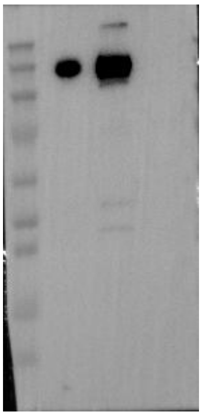

Brain OBs OCs

β-ACTIN

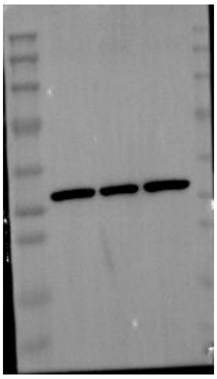

Supplementary Figure 1B

Osteoblast

Slit2$^{f/f}$ Slit2^{osx}

SLIT2

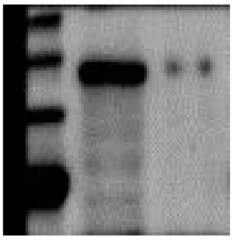

Osteoblast

Slit2$^{f/f}$ Slit2^{osx}

β-ACTIN

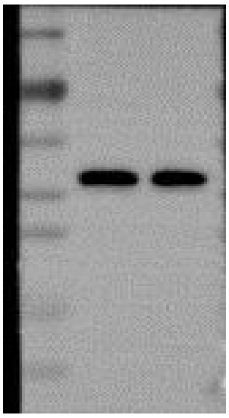

Sympathetic neuron

Slit2$^{f/f}$ Slit2^{osx}

SLIT2

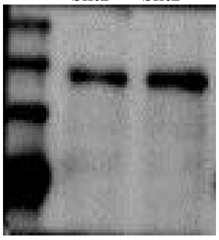

Sympathetic neuron

Slit2$^{f/f}$ Slit2^{osx}

β-ACTIN

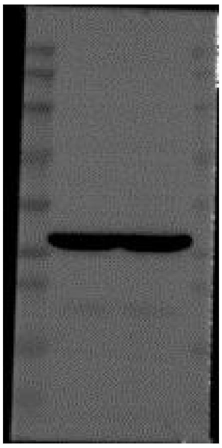

Supplementary Figure 1D

BMSCs

Slit2$^{f/f}$ Slit2^{prx1}

SLIT2

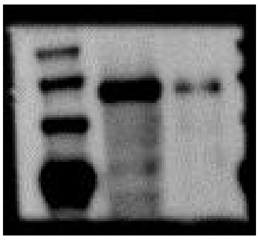

BMSCs

Slit2$^{f/f}$ Slit2^{prx1}

β-ACTIN

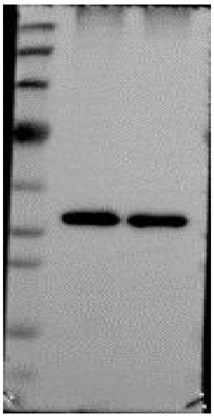

Sympathetic neuron

Slit2$^{f/f}$ Slit2^{prx1}

SLIT2

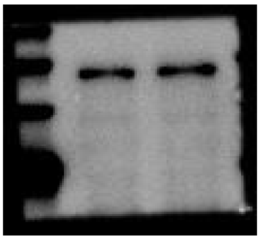

Sympathetic neuron

Slit2$^{f/f}$ Slit2^{prx1}

β-ACTIN

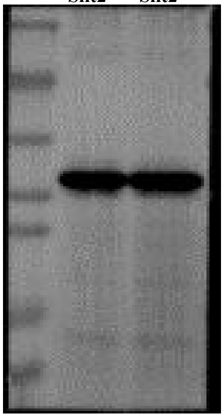

Supplementary Figure 2B

Sympathetic neuron

$Slit2^{f/f}$ $Slit2^{syn1}$

SLIT2

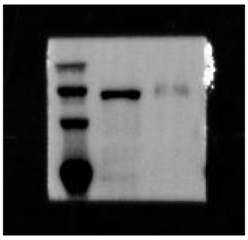

Sympathetic neuron

$Slit2^{f/f}$ $Slit2^{syn1}$

β-ACTIN

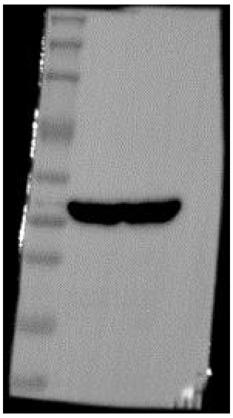

Osteoblast

$Slit2^{f/f}$ $Slit2^{syn1}$

SLIT2

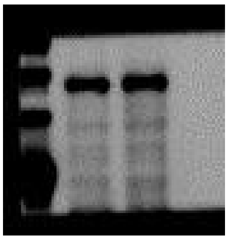

Osteoblast

$Slit2^{f/f}$ $Slit2^{syn1}$

β-ACTIN

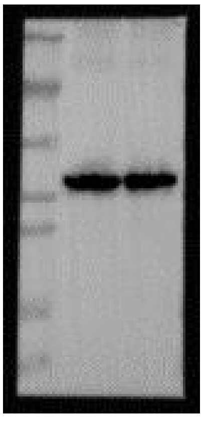

Supplementary Figure 3C

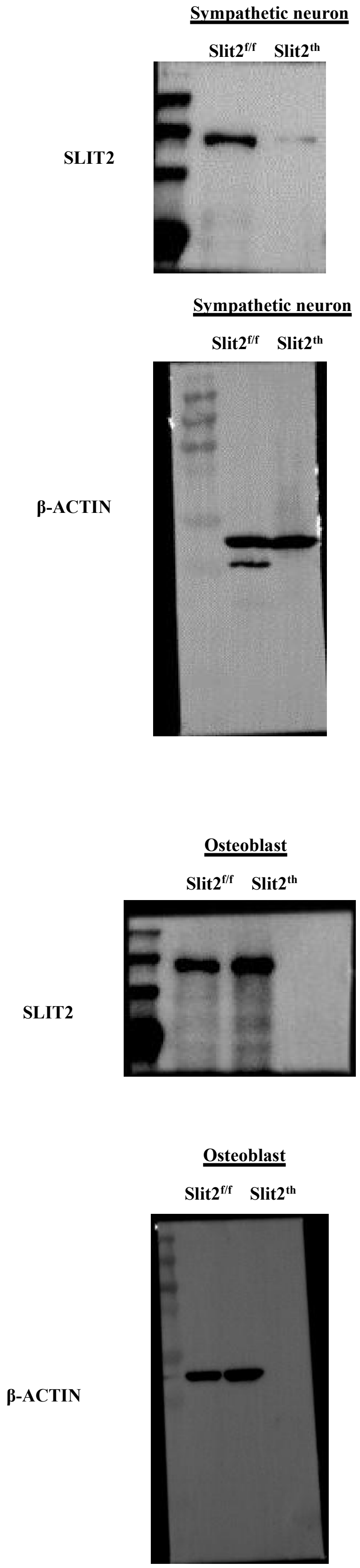

Supplementary Figure 6F

Sympathetic neuron

Slit2$^{f/f}$ Slit2^{adv}

SLIT2

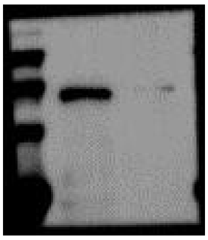

Sympathetic neuron

Slit2$^{f/f}$ Slit2^{adv}

β-ACTIN

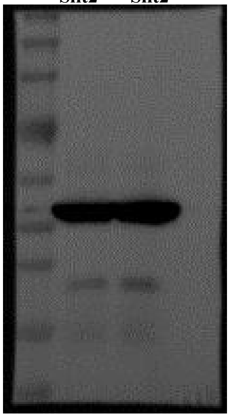

Osteoblast

Slit2$^{f/f}$ Slit2^{adv}

SLIT2

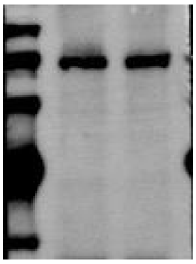

Osteoblast

Slit2$^{f/f}$ Slit2^{adv}

β-ACTIN

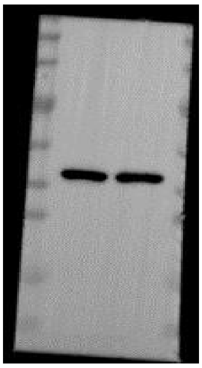

Supplementary Figure 6H

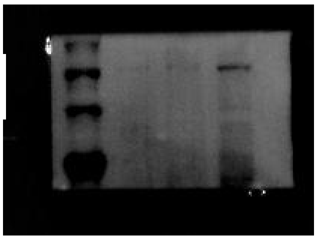

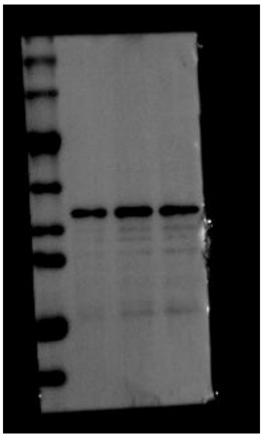

Supplementary Figure 8A

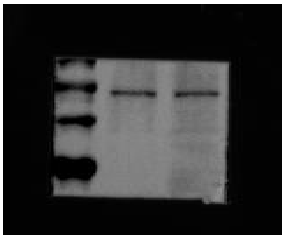

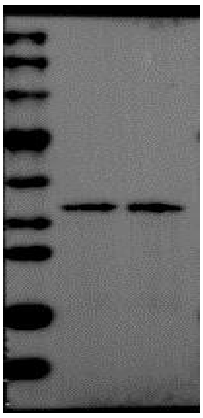

Supplementary Figure 8B
