# Supplementary material for: Corrigendum to Axon guidance cue SLIT2 regulates the murine skeletal stem cell niche through sympathetic innervation
Source: J Clin Invest. 2026 Jan 16;136(2):e203674. doi: 10.1172/JCI203674 (PMC12807457; doi:10.1172/JCI203674)
Supplement: Supplemental data [file jci-136-203674-s259.pdf]

Axon guidance cue SLIT2 regulates the murine skeletal stem cell niche through sympathetic innervation

Zuoxing Wu[1,2*], Na Li[1,11*], Zhengqiong Luo[1,2], Zihan Chen[2,3], Xuemei He[1,2], Jie Han[1,2], Xixi Lin[1,2], Fan Shi[2,3], Haitao Huang[4], Baohong Shi[1,2], Yu Li[1,2], Xin Wang[9], Lin Meng[10], Dachuan Zhang[5], Lanfen Chen[4], Dawang Zhou[4], Weinan Cheng[1,8†], Matthew B. Greenblatt[6,7†] and Ren Xu[1-3†]

Affiliations:

[1]The First Affiliated Hospital of Xiamen University-ICMRS Collaborating Center for Skeletal Stem Cells, Xiamen Cell Therapy Research Center, The First Affiliated Hospital of Xiamen University, School of Medicine, Faculty of Medicine and Life Sciences, Xiamen University, Xiamen, Fujian, China.

[2]Xiamen Key Laboratory of Regeneration Medicine, Fujian Provincial Key Laboratory of Organ and Tissue Regeneration, School of Medicine, Xiamen University, Xiamen, Fujian, China.

[3]Research Centre for Regenerative Medicine, Guangxi Key Laboratory of Regenerative Medicine, Guangxi Medical University, Nanning, Guangxi, China.

[4]State Key Laboratory of Cellular Stress Biology, Innovation Center for Cell Signaling Network, School of Life Sciences, Xiamen University, Xiamen, Fujian, China.

[5]Department of Pathophysiology, Key Laboratory of Cell Differentiation and Apoptosis, Chinese Ministry of Education, Shanghai Jiao Tong University

School of Medicine, Shanghai, China.
[6]Department of Pathology and Laboratory Medicine, Weill Cornell Medical College, New York, NY, USA.
[7]Skeletal Health and Orthopedic Research Program, Hospital for Special Surgery, New York, NY, USA.
[8]Department of Sports Medicine, National Center for Orthopaedics, Shanghai Jiao Tong University Affiliated Sixth People’s Hospital, Shanghai, China.
[9]Graduate School of Science and Engineering, Ritsumeikan University, Shiga, Japan
[10]College of Science and Engineering, Ritsumeikan University, Shiga, Japan
[11]Shenzhen Key Laboratory of Bone Tissue Repair and Translational Research,Department of Orthopaedic Surgery, The Seventh Affiliated Hospital of Sun Yat-sen University, Shenzhen, China

[*]These authors contributed equally to this work.

[†]**Corresponding authors:**

Ren Xu, Ph.D

State Key Laboratory of Cellular Stress Biology,

School of Medicine,

Xiamen University,

Yuejin Building. A503, Xiang’an South Road, Xiang’an District, Xiamen, 361102

E-mail: xuren526@xmu.edu.cn

Matthew B. Greenblatt, MD Ph.D

Department of Pathology and Laboratory Medicine

Weill Cornell Medical College

Skeletal Health and Orthopedic Research Program

Hospital for Special Surgery

LC925, 1300 York Ave, New York, NY 10065

E-mail: Mag3003@med.cornell.edu

Weinan Cheng, MD Ph.D

Department of Sports Medicine National Center for Orthopaedics,

Shanghai Jiao Tong University Affiliated Sixth People’s Hospital

Shanghai, 200233, China

E-mail: 7250013396@shsmu.edu.cn

Figure S1

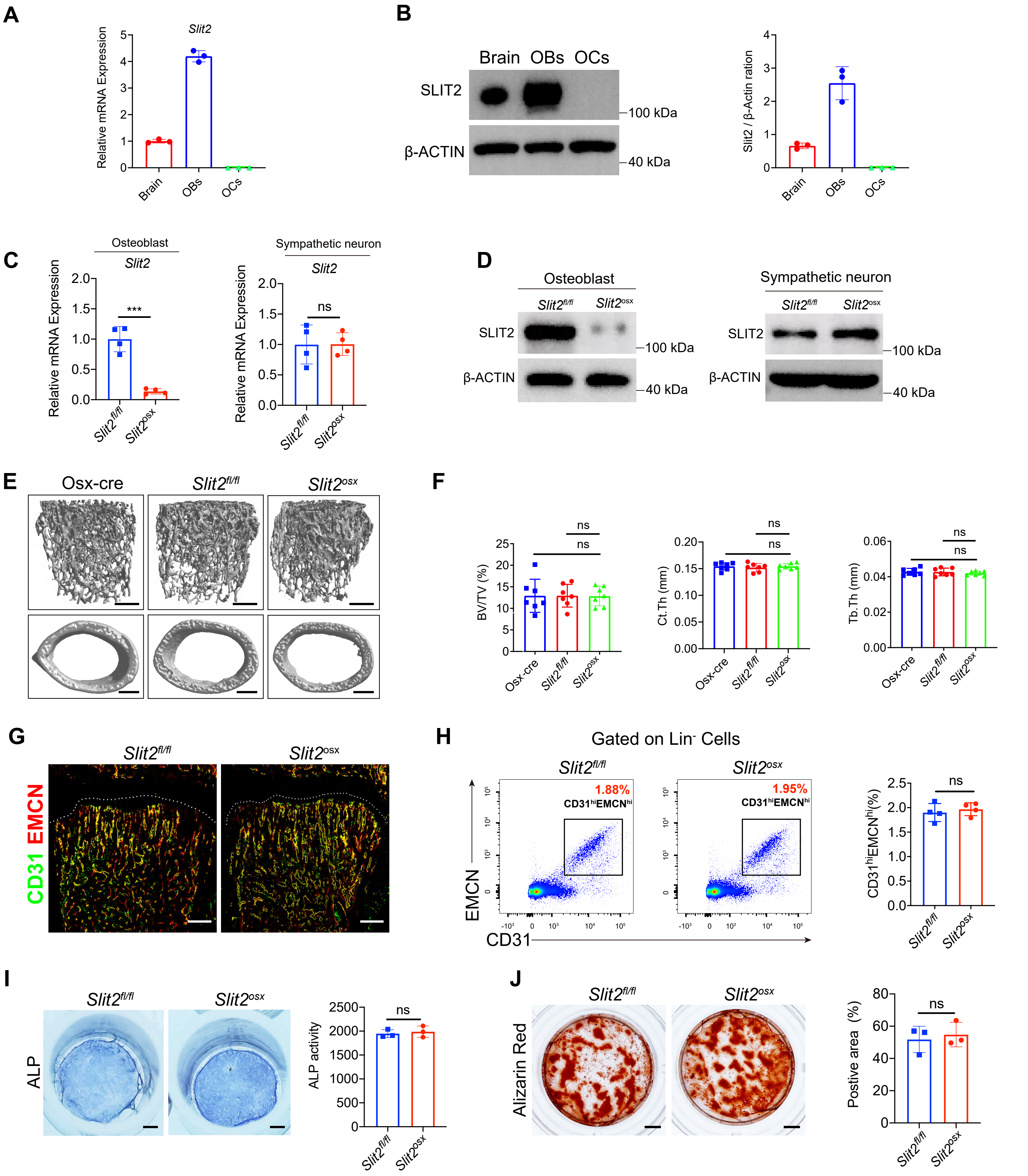
A
Slit2
Relative mRNA Expression
Brain
OBs
OCs
B
Brain OBs OCs
SLIT2
β-ACTIN
100 kDa
40 kDa
Slit2 / β-Actin ration
C
Osteoblast
Sympathetic neuron
***
ns
D
SLIT2
β-ACTIN
E
Osx-cre
F
BV/TV (%)
Ct.Th (mm)
Tb.Th (mm)
G
CD31 EMCN
H
Gated on Lin- Cells
1.88%
1.95%
CD31hiEMCNhi
EMCN
CD31
I
ALP
ALP activity
J
Alizarin Red
Postive area (%)

Figure S2

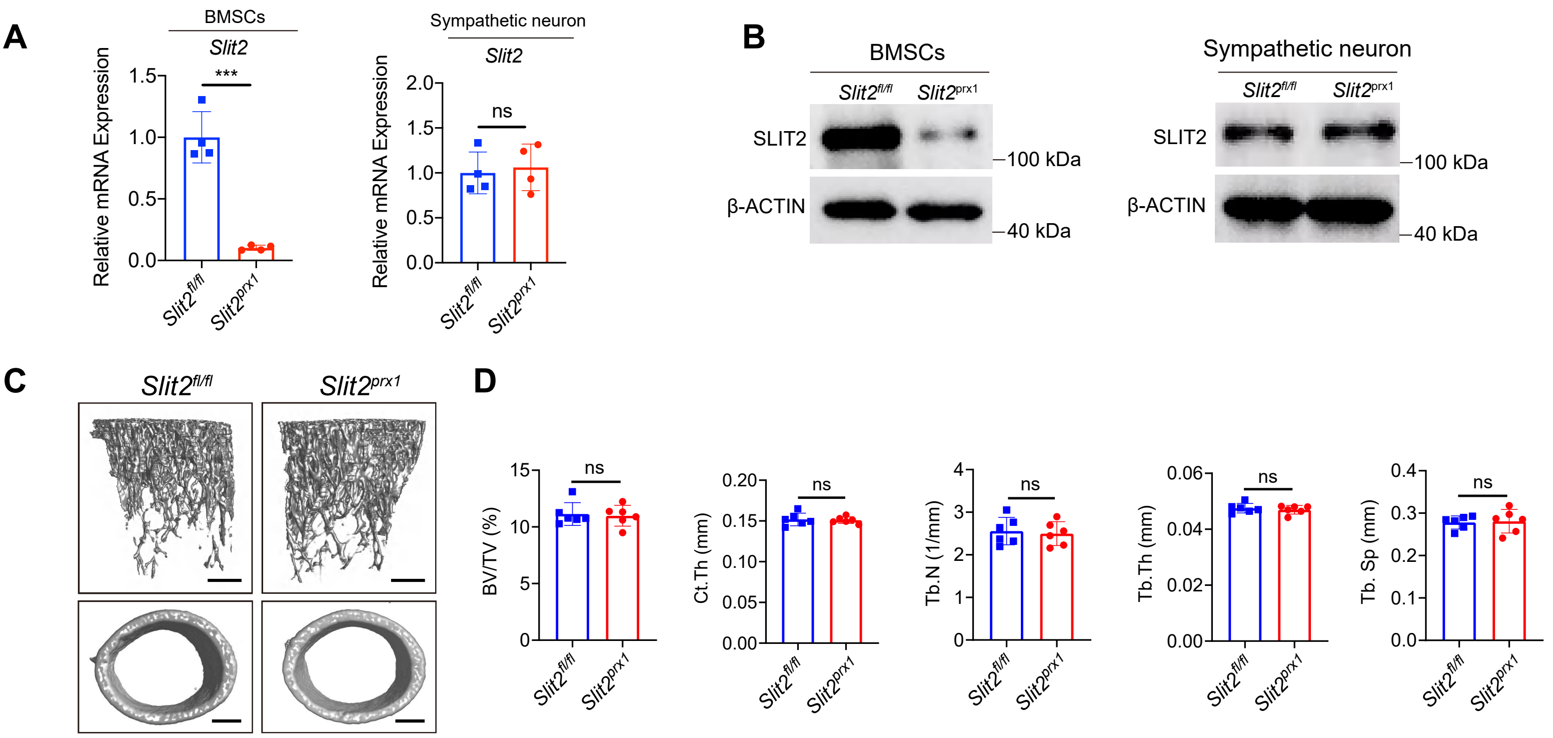
A
BMSCs
Slit2
Relative mRNA Expression
***
Sympathetic neuron
Slit2
ns
Slit2fl/fl
Slit2prx1
B
BMSCs
Sympathetic neuron
Slit2fl/fl
Slit2prx1
SLIT2
β-ACTIN
100 kDa
40 kDa
C
Slit2fl/fl
Slit2prx1
D
BV/TV (%)
Ct.Th (mm)
Tb.N (1/mm)
Tb.Th (mm)
Tb. Sp (mm)
ns

Figure S3

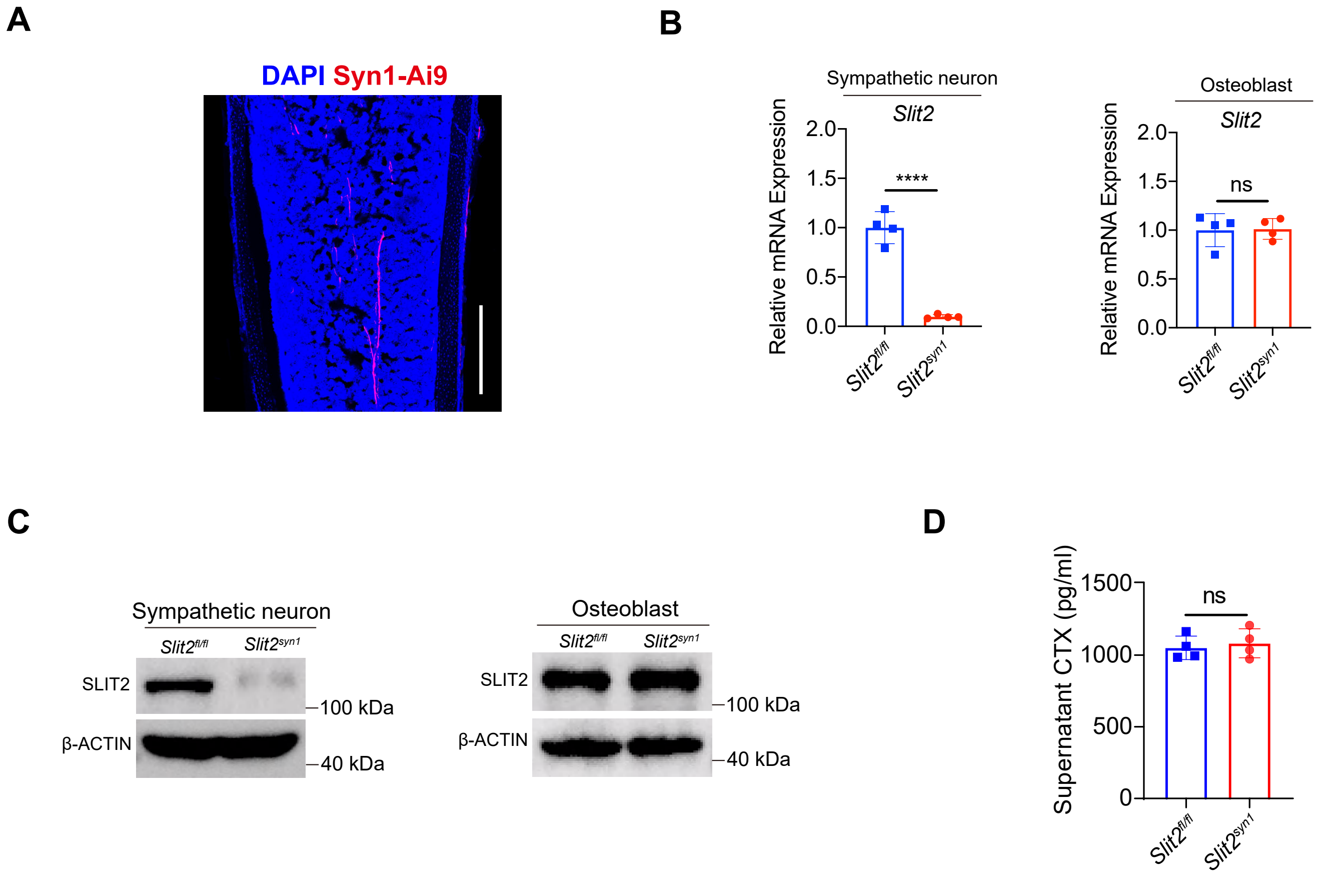
A
DAPI Syn1-Ai9
B
Sympathetic neuron
Slit2
Relative mRNA Expression
2.0
1.5
1.0
0.5
0.0
****
Slit2fl/fl
Slit2syn1
Osteoblast
Slit2
Relative mRNA Expression
ns
C
Sympathetic neuron
Slit2fl/fl
Slit2syn1
SLIT2
β-ACTIN
100 kDa
40 kDa
Osteoblast
D
Supernatant CTX (pg/ml)
1500
1000
500
0
ns

Figure S4

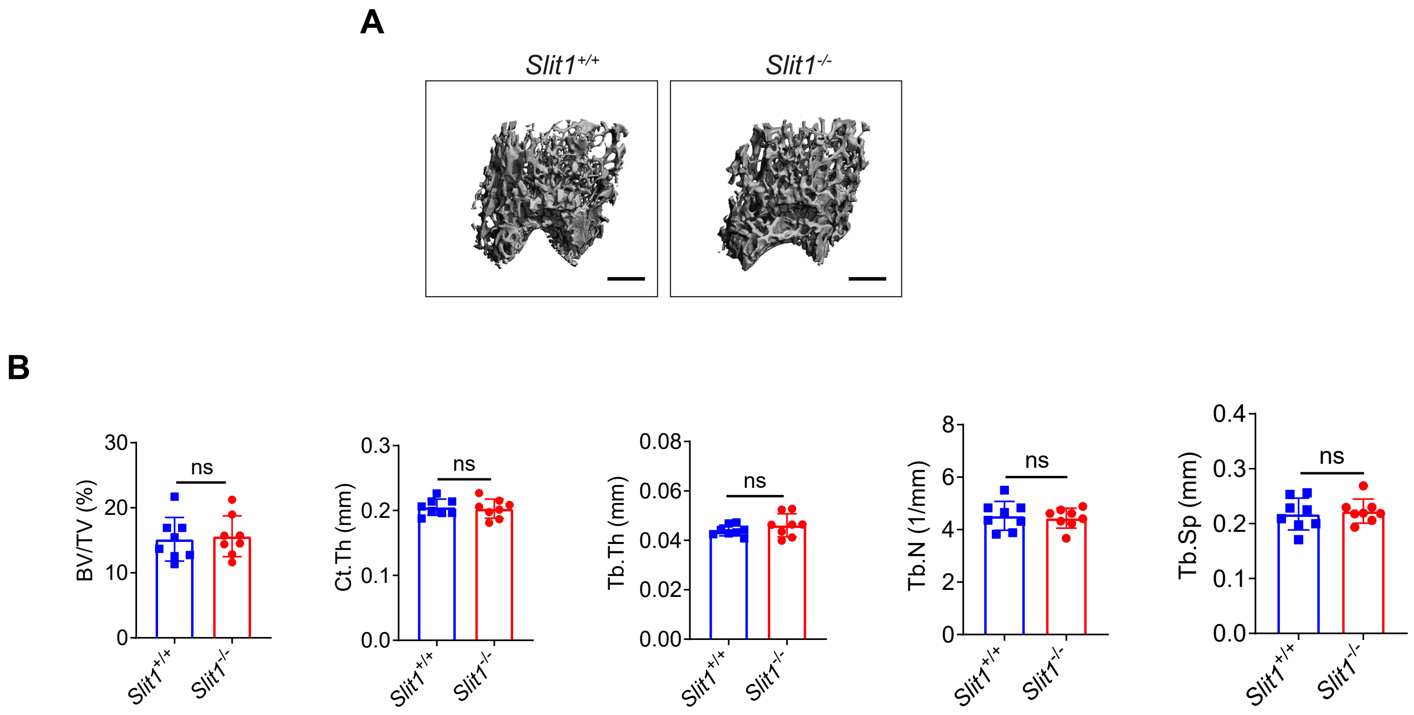
A
Slit1+/+
Slit1-/-
B
BV/TV (%)
Ct.Th (mm)
Tb.Th (mm)
Tb.N (1/mm)
Tb.Sp (mm)
ns
Slit1+/+
Slit1-/-

Figure S5

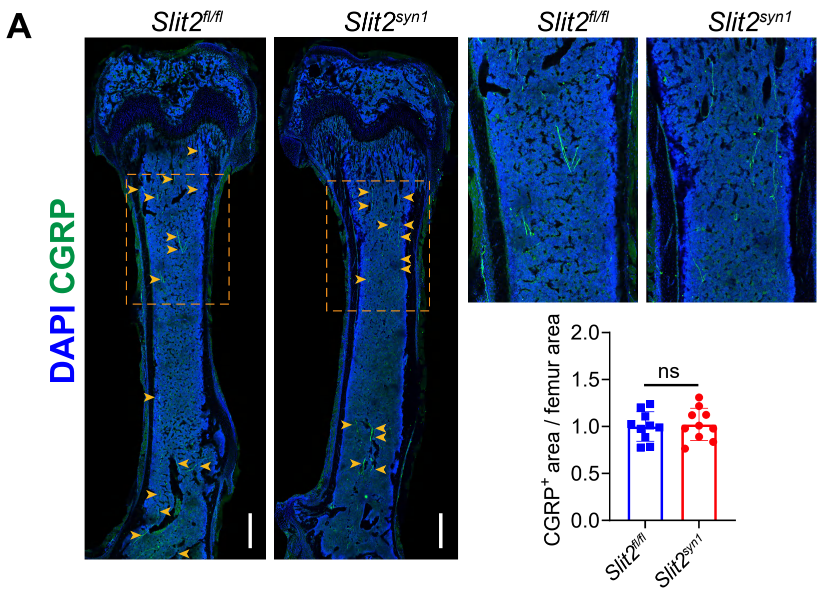
A
Slit2fl/fl
Slit2syn1
Slit2fl/fl
Slit2syn1
DAPI CGRP
CGRP+ area / femur area
2.0
1.5
1.0
0.5
0.0
ns
Slit2fl/fl
Slit2syn1

Figure S6

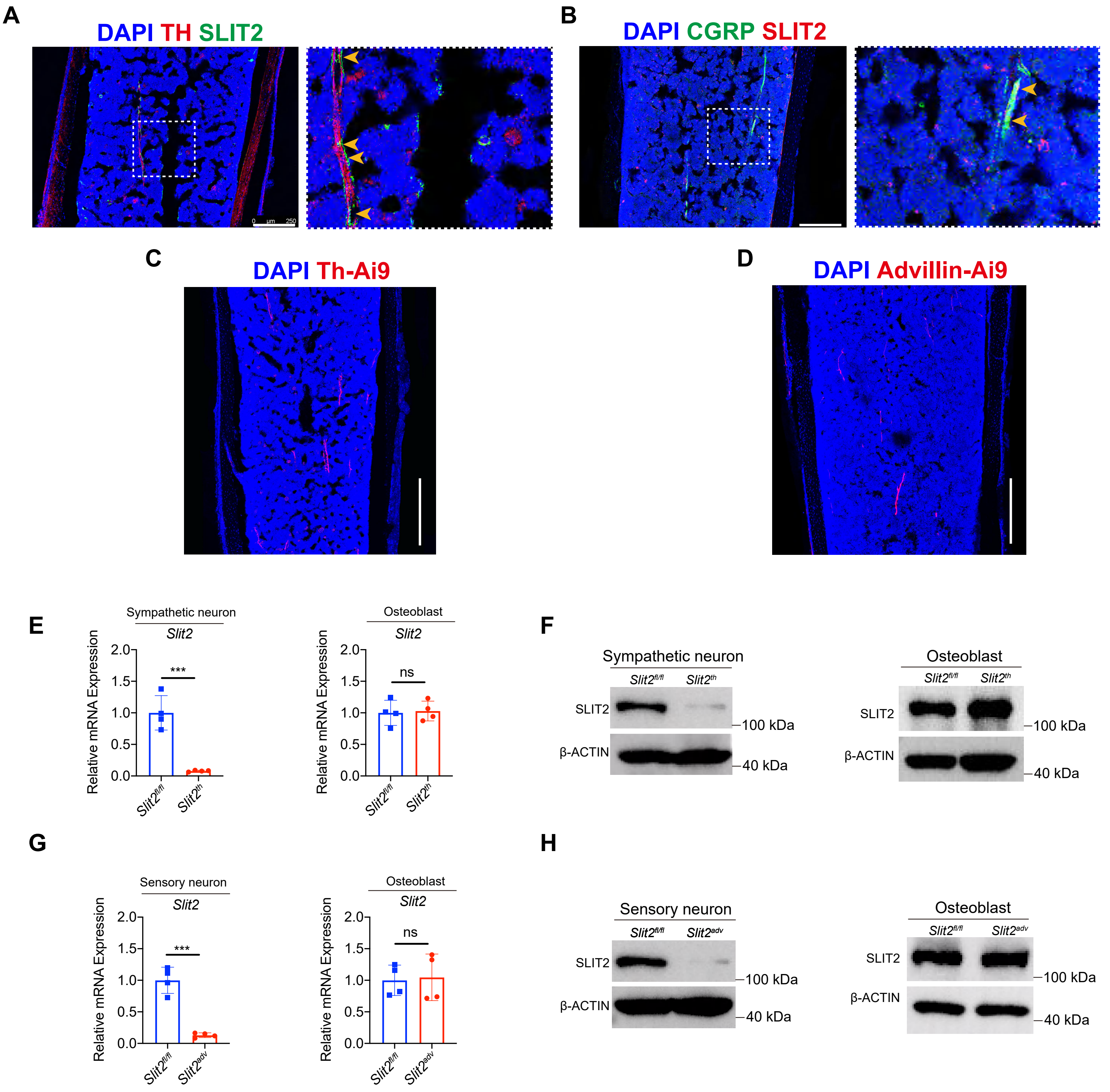
A
DAPI TH SLIT2
B
DAPI CGRP SLIT2
C
DAPI Th-Ai9
D
DAPI Advillin-Ai9
E
Sympathetic neuron
Slit2
Relative mRNA Expression
***
Slit2fl/fl
Slit2th
Osteoblast
Slit2
ns
F
Sympathetic neuron
Slit2fl/fl
Slit2th
SLIT2
β-ACTIN
100 kDa
40 kDa
Osteoblast
G
Sensory neuron
Slit2
Slit2adv
Osteoblast
H
Sensory neuron
Slit2adv
Osteoblast

Figure S7

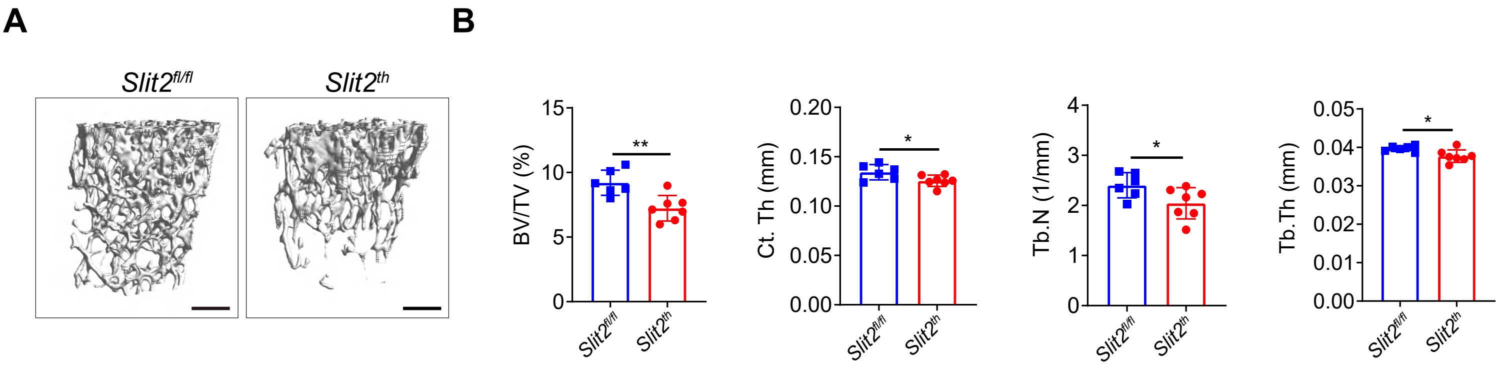

C

Two-way ANOVA of bone volume fraction (BV/TV) in male and female *Slit2^fl/fl^* and *Slit2^th^* mice

Factor	DF	Sum Sq	Mean Sq	F value	P value
Sex (Male/Female)	1	219.39	219.39	84.5	＜0.001***
Genotype (*Slit2^fl/fl^*/*Slit2^th^*)	1	83.19	83.19	32.04	＜0.001***
Sex: Genotype	1	3.29	3.29	1.27	0.268
Residuals	38	98.66	2.6		

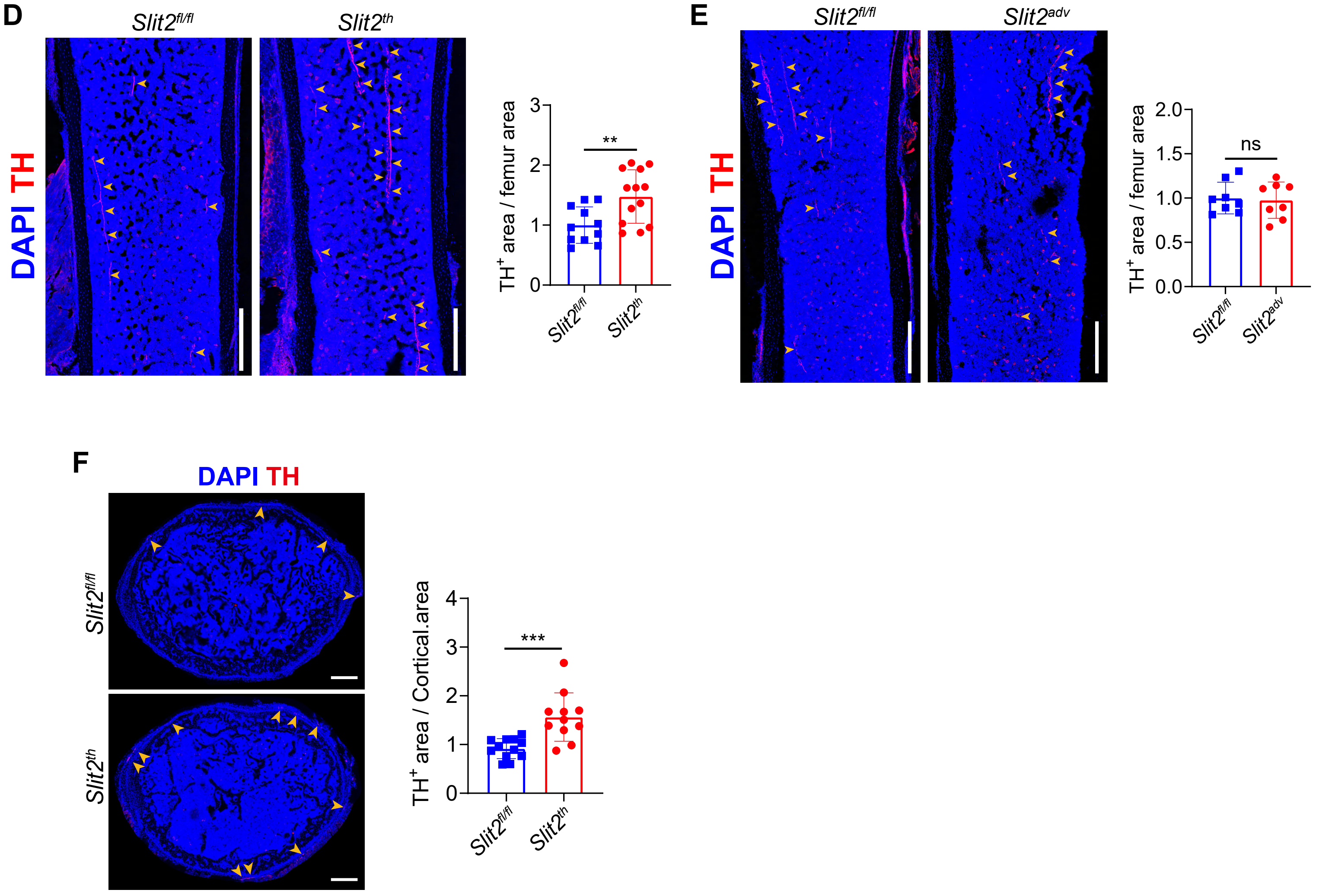

Figure S8

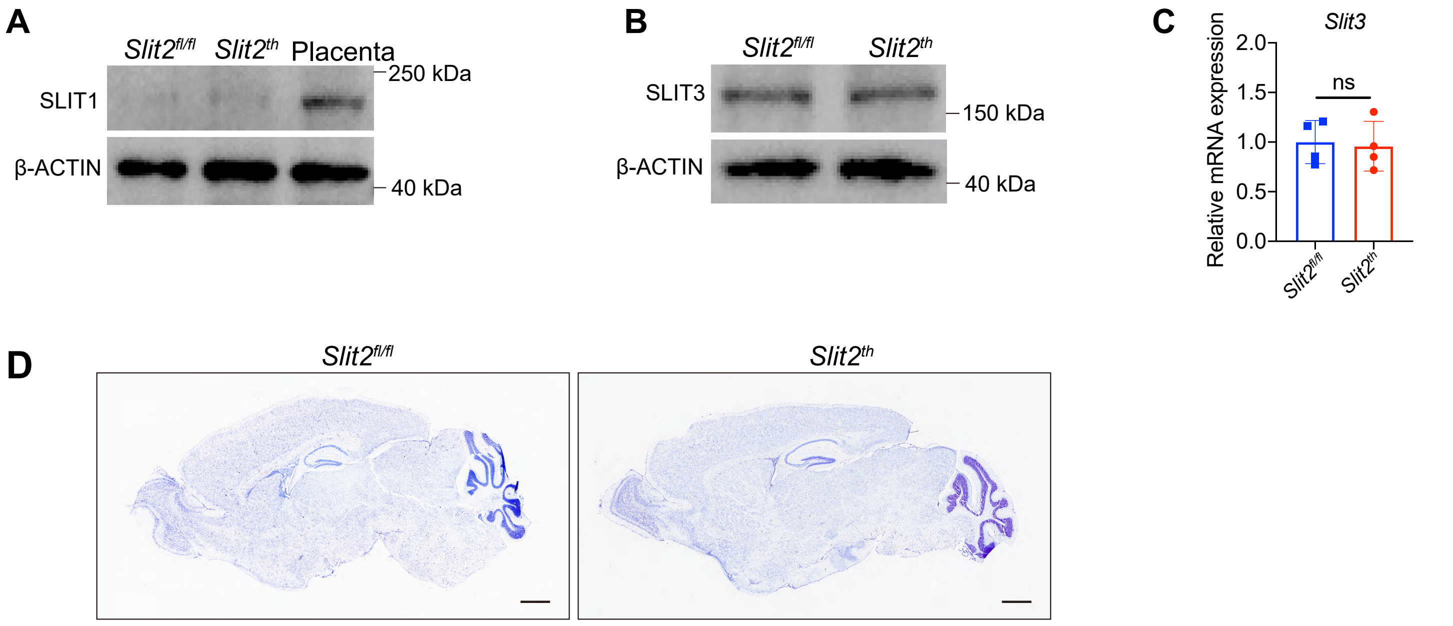
A
Slit2fl/fl
Slit2th
Placenta
SLIT1
β-ACTIN
250 kDa
40 kDa
B
Slit2fl/fl
Slit2th
SLIT3
β-ACTIN
150 kDa
40 kDa
C
Slit3
Relative mRNA expression
2.0
1.5
1.0
0.5
0.0
ns
Slit2fl/fl
Slit2th
D
Slit2fl/fl
Slit2th

Figure S9

A

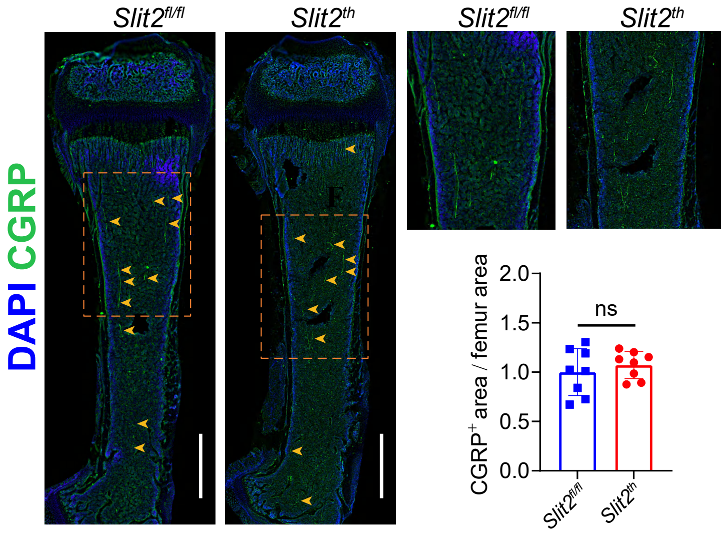
Slit2fl/fl
Slit2th
Slit2fl/fl
Slit2th
DAPI CGRP
CGRP+ area / femur area
2.0
1.5
1.0
0.5
0.0
ns
Slit2fl/fl
Slit2th

B

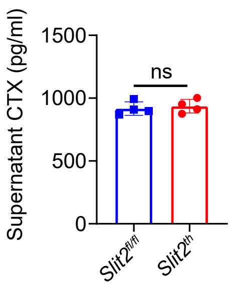
Supernatant CTX (pg/ml)
1500
1000
500
0
ns
Slit2fl/fl
Slit2th

Figure S10

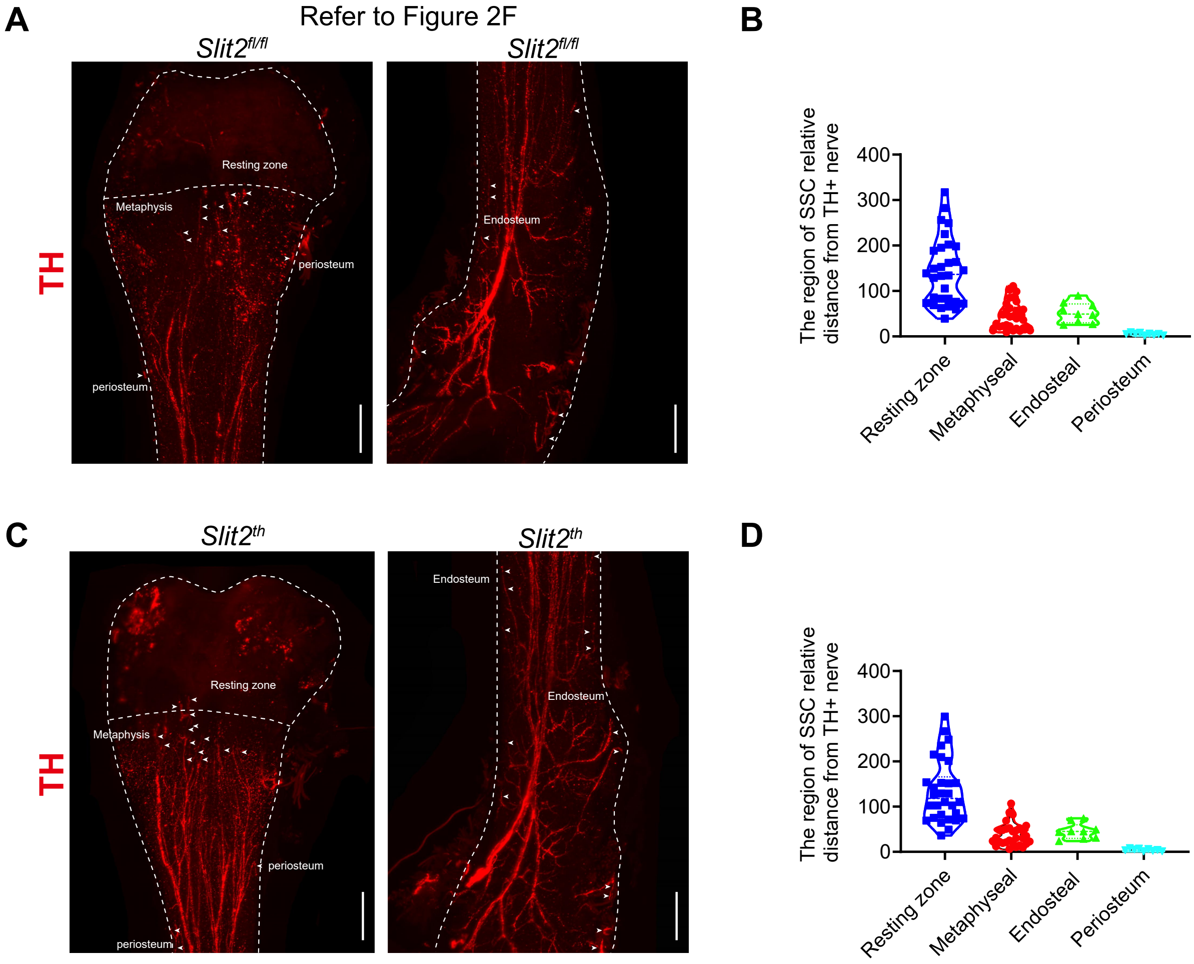
A
Refer to Figure 2F
Slit2fl/fl
Slit2fl/fl
TH
Resting zone
Metaphysis
periosteum
periosteum
Endosteum
B
The region of SSC relative distance from TH+ nerve
400
300
200
100
0
Resting zone
Metaphyseal
Endosteal
Periosteum
C
Slit2th
Slit2th
TH
Resting zone
Metaphysis
periosteum
periosteum
Endosteum
Endosteum
D
The region of SSC relative distance from TH+ nerve
400
300
200
100
0
Resting zone
Metaphyseal
Endosteal
Periosteum

Figure S11

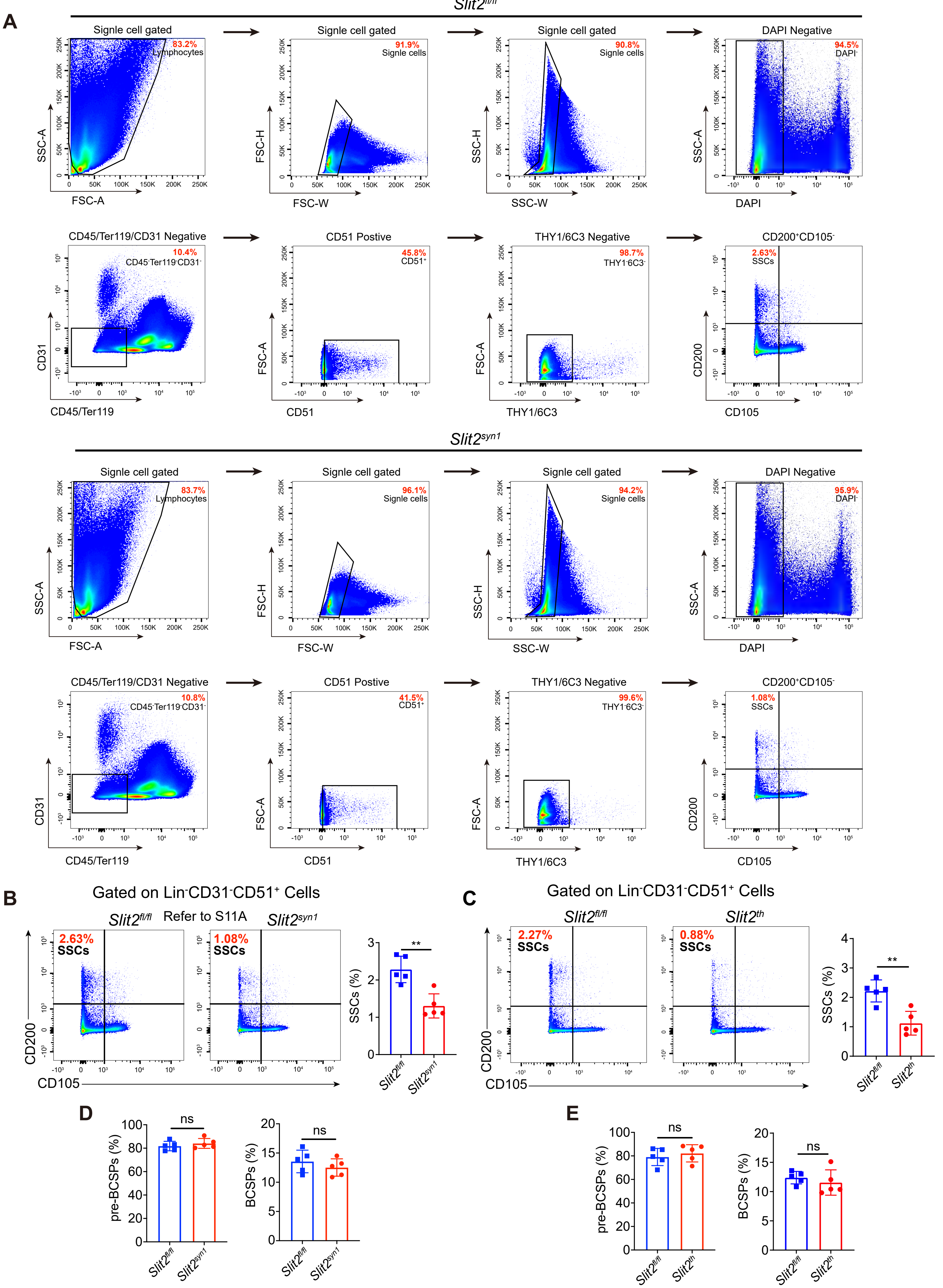
A
Slit2fl/fl
Signle cell gated
83.2% Lymphocytes
SSC-A
FSC-A
91.9% Signle cells
FSC-H
FSC-W
90.8% Signle cells
SSC-H
SSC-W
DAPI Negative
94.5% DAPI-
DAPI
CD45/Ter119/CD31 Negative
10.4% CD45-Ter119-CD31-
CD31
CD45/Ter119
CD51 Postive
45.8% CD51+
FSC-A
CD51
THY1/6C3 Negative
98.7% THY1-6C3-
THY1/6C3
CD200+CD105-
2.63% SSCs
CD200
CD105
Slit2syn1
83.7% Lymphocytes
96.1% Signle cells
94.2% Signle cells
95.9% DAPI-
10.8% CD45-Ter119-CD31-
41.5% CD51+
99.6% THY1-6C3-
1.08% SSCs
B
Gated on Lin-CD31-CD51+ Cells
Slit2fl/fl
Refer to S11A
Slit2syn1
2.63% SSCs
1.08% SSCs
SSCs (%)
**
C
Slit2fl/fl
Slit2th
2.27% SSCs
0.88% SSCs
D
pre-BCSPs (%)
BCSPs (%)
ns
E

Figure S12

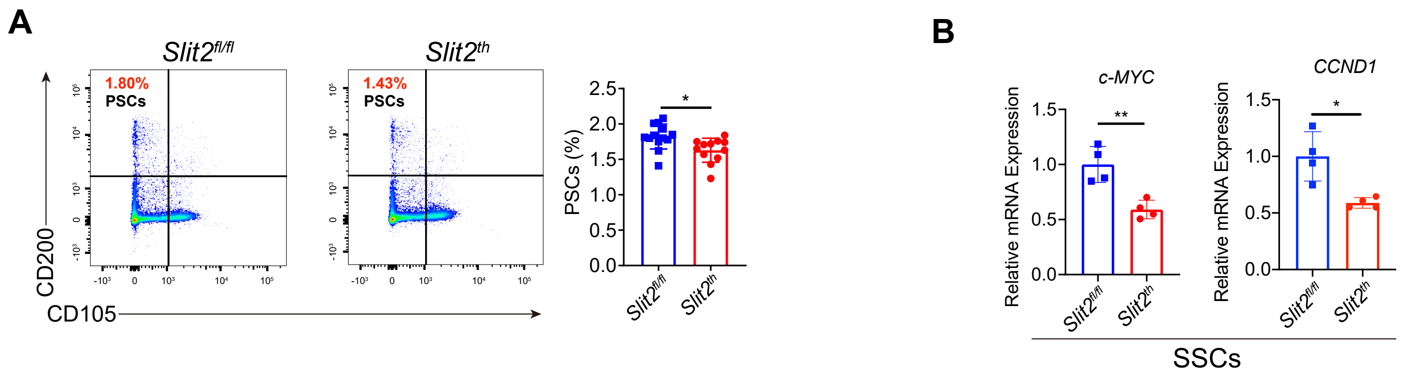
A
Slit2fl/fl
Slit2th
1.80%
PSCs
1.43%
PSCs
CD200
CD105
PSCs (%)
*
Slit2fl/fl
Slit2th
B
c-MYC
CCND1
Relative mRNA Expression
**
*
SSCs

Figure S13

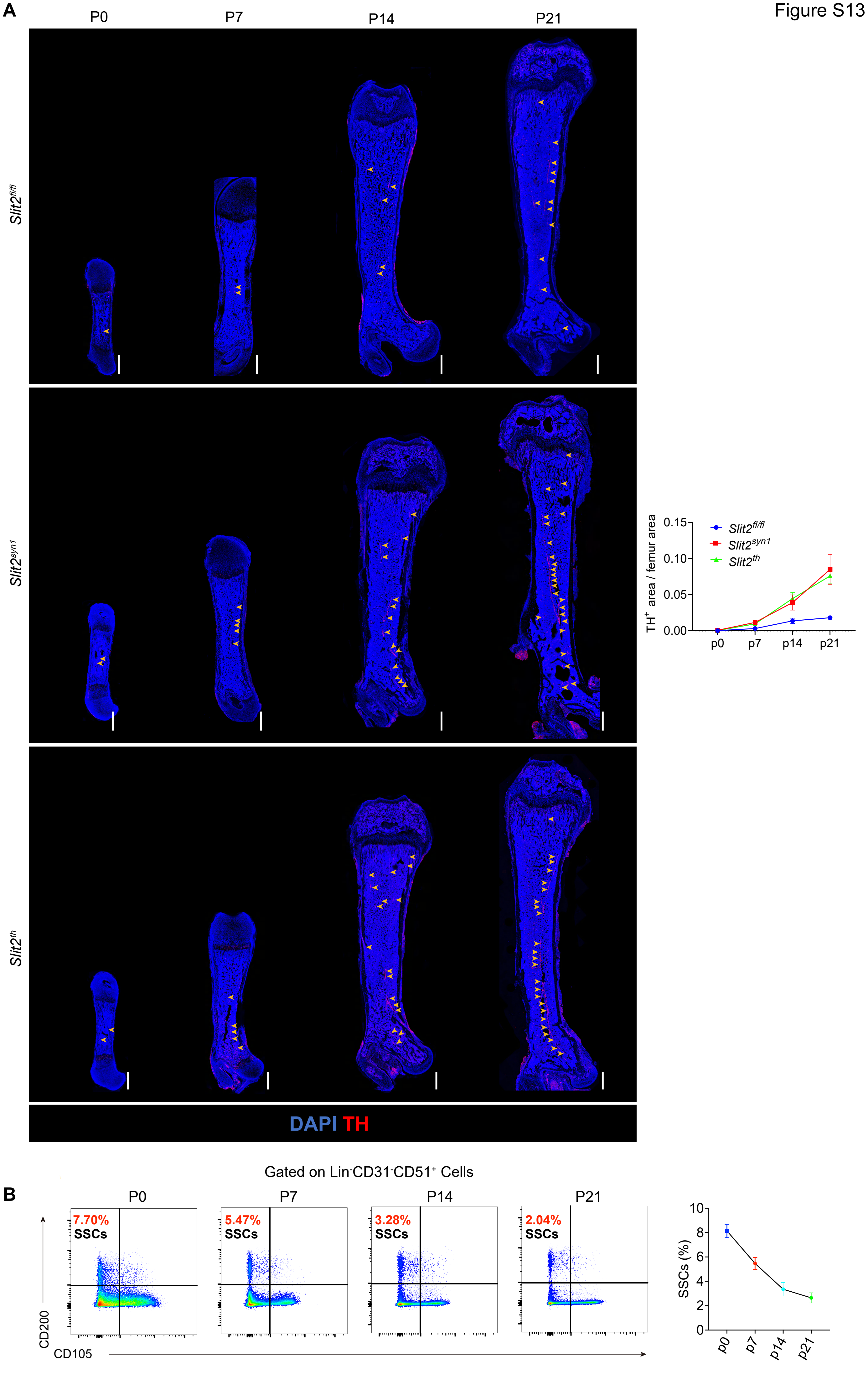

A
P0
P7
P14
P21
Slit2fl/fl
Slit2syn1
Slit2th
DAPI TH
TH+ area / femur area
0.15
0.10
0.05
0.00
p0
p7
p14
p21
Slit2fl/fl
Slit2syn1
Slit2th
Gated on Lin-CD31-CD51+ Cells
B
P0
P7
P14
P21
7.70%
SSCs
5.47%
SSCs
3.28%
SSCs
2.04%
SSCs
CD200
CD105
SSCs (%)
10
8
6
4
2
0
p0
p7
p14
p21

Figure S14

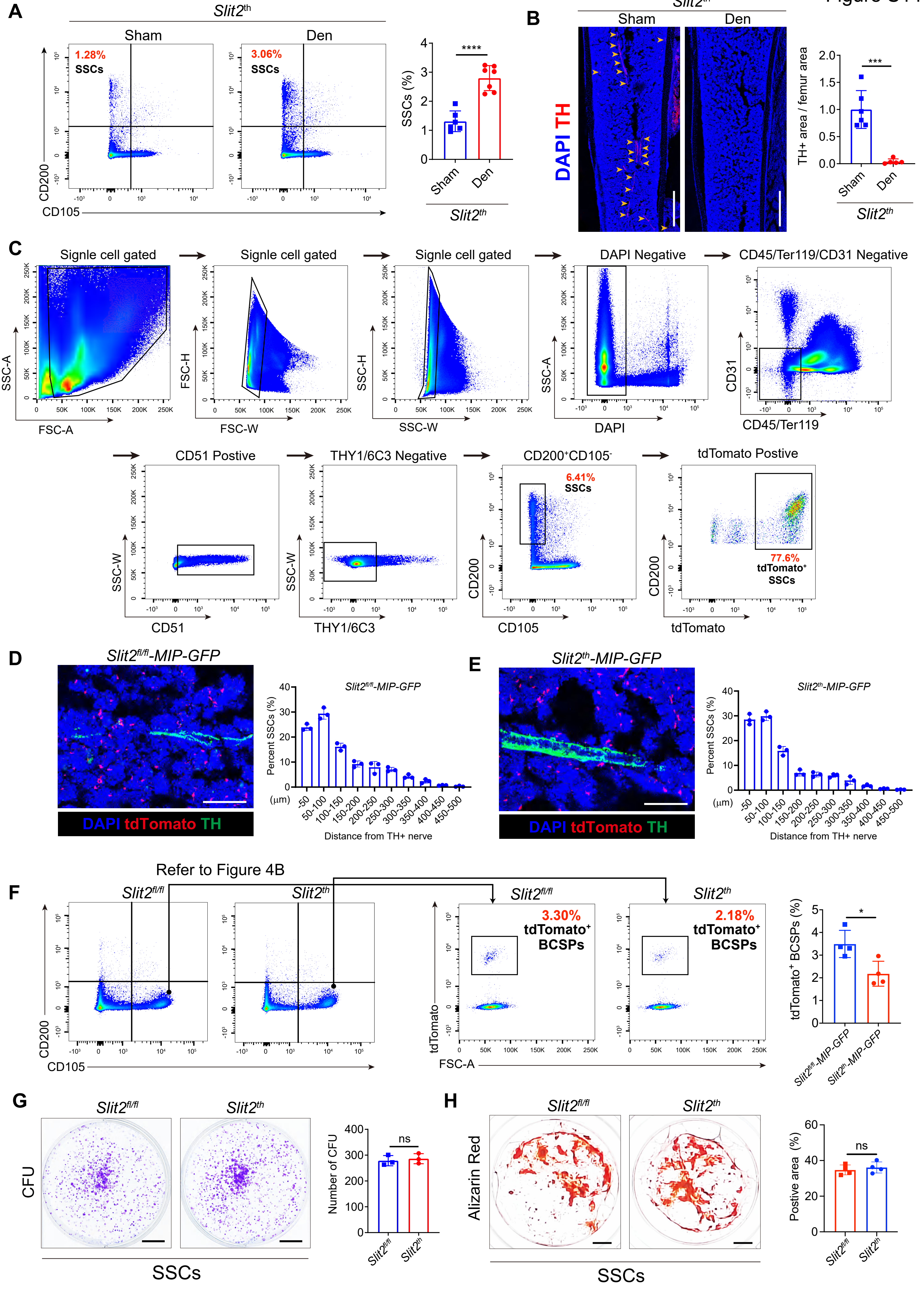
A
Slit2th
Sham
Den
1.28%
SSCs
3.06%
SSCs
CD200
CD105
SSCs (%)
****
Sham
Den
Slit2th
B
Slit2th
Sham
Den
DAPI TH
TH+ area / femur area
***
Sham
Den
Slit2th
C
Signle cell gated
SSC-A
FSC-A
Signle cell gated
FSC-H
FSC-W
Signle cell gated
SSC-H
SSC-W
DAPI Negative
SSC-A
DAPI
CD45/Ter119/CD31 Negative
CD31
CD45/Ter119
CD51 Postive
SSC-W
CD51
THY1/6C3 Negative
SSC-W
THY1/6C3
CD200+CD105-
6.41%
SSCs
CD200
CD105
tdTomato Postive
77.6%
tdTomato+
SSCs
CD200
tdTomato
D
Slit2fl/fl-MIP-GFP
DAPI tdTomato TH
Slit2fl/fl-MIP-GFP
Percent SSCs (%)
(μm)
-50
50-100
100-150
150-200
200-250
250-300
300-350
350-400
400-450
450-500
Distance from TH+ nerve
E
Slit2th-MIP-GFP
DAPI tdTomato TH
Slit2th-MIP-GFP
Percent SSCs (%)
(μm)
Distance from TH+ nerve
Refer to Figure 4B
F
Slit2fl/fl
Slit2th
CD200
CD105
Slit2fl/fl
3.30%
tdTomato+
BCSPs
Slit2th
2.18%
tdTomato+
BCSPs
tdTomato
FSC-A
tdTomato+ BCSPs (%)
*
Slit2fl/fl-MIP-GFP
Slit2th-MIP-GFP
G
Slit2fl/fl
Slit2th
CFU
SSCs
Number of CFU
ns
Slit2fl/fl
Slit2th
H
Slit2fl/fl
Slit2th
Alizarin Red
SSCs
Postive area (%)
ns
Slit2fl/fl
Slit2th

Figure S15

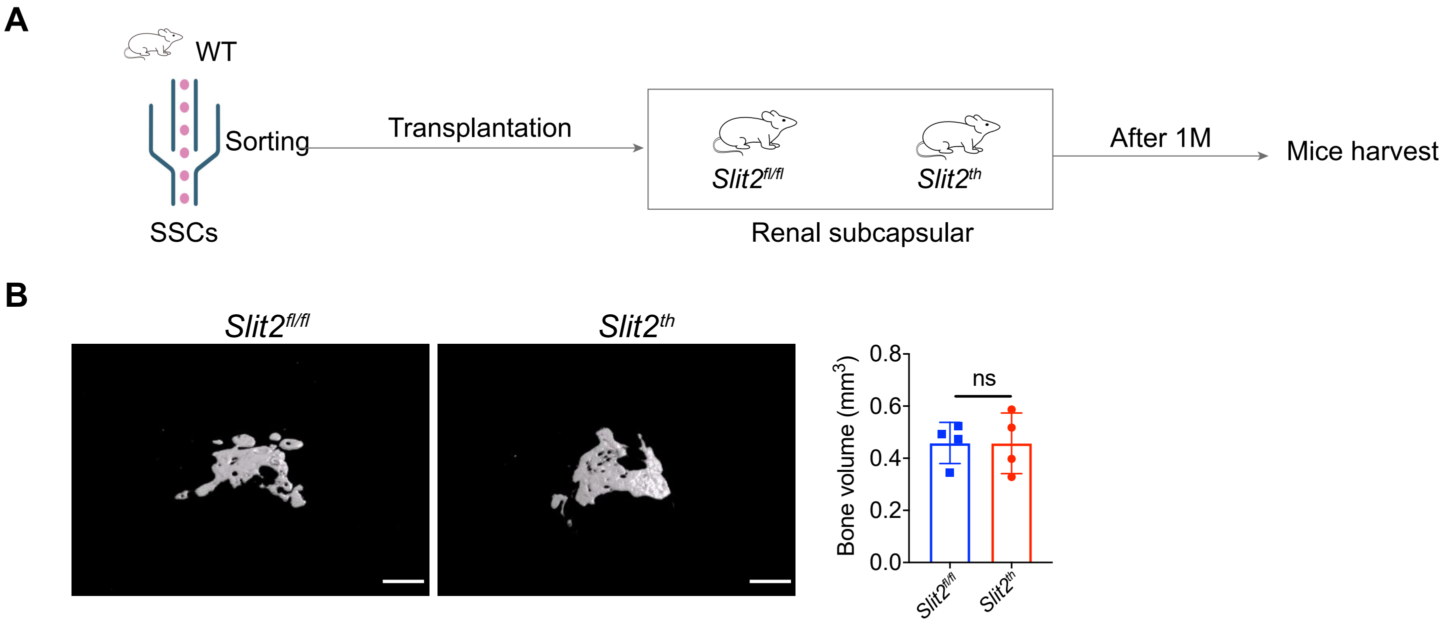
A
WT
Sorting
Transplantation
$Slit2^{fl/fl}$
$Slit2^{th}$
After 1M
Mice harvest
SSCs
Renal subcapsular
B
$Slit2^{fl/fl}$
$Slit2^{th}$
ns
Bone volume (mm^3)
0.8
0.6
0.4
0.2
0.0
$Slit2^{fl/fl}$
$Slit2^{th}$

Figure S16

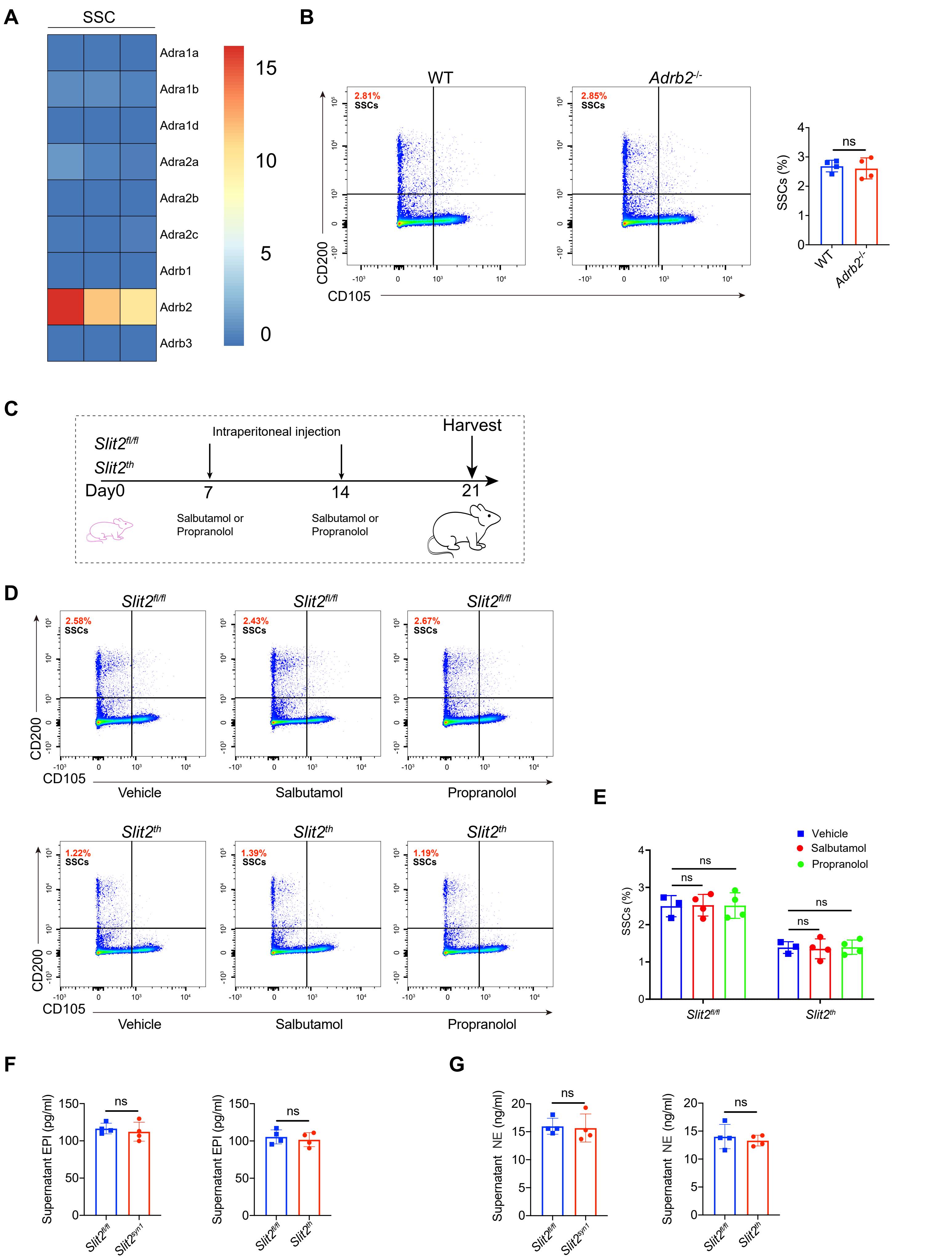
A
SSC
Adra1a
Adra1b
Adra1d
Adra2a
Adra2b
Adra2c
Adrb1
Adrb2
Adrb3
15
10
5
0
B
WT
Adrb2-/-
2.81% SSCs
2.85% SSCs
CD200
CD105
ns
SSCs (%)
C
Slit2fl/fl
Slit2th
Intraperitoneal injection
Harvest
Day0
7
14
21
Salbutamol or Propranolol
Salbutamol or Propranolol
D
2.58% SSCs
2.43% SSCs
2.67% SSCs
1.22% SSCs
1.39% SSCs
1.19% SSCs
Vehicle
Salbutamol
Propranolol
E
Vehicle
Salbutamol
Propranolol
ns
F
Supernatant EPI (pg/ml)
Slit2fl/fl
Slit2syn1
Slit2th
G
Supernatant NE (ng/ml)

Figure S17

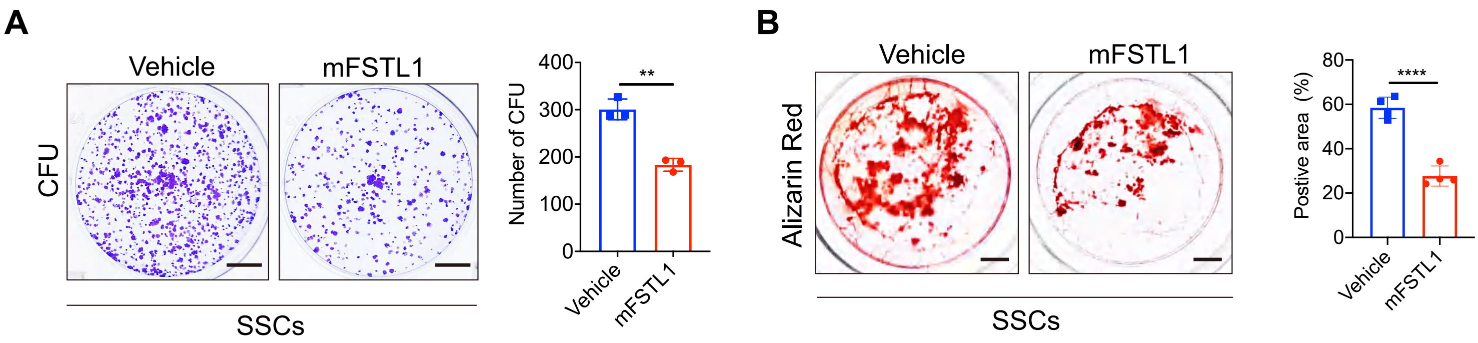
A
Vehicle
mFSTL1
CFU
SSCs
Number of CFU
400
300
200
100
0
**
Vehicle
mFSTL1
B
Vehicle
mFSTL1
Alizarin Red
SSCs
Postive area (%)
80
60
40
20
0
****
Vehicle
mFSTL1

Figure S18

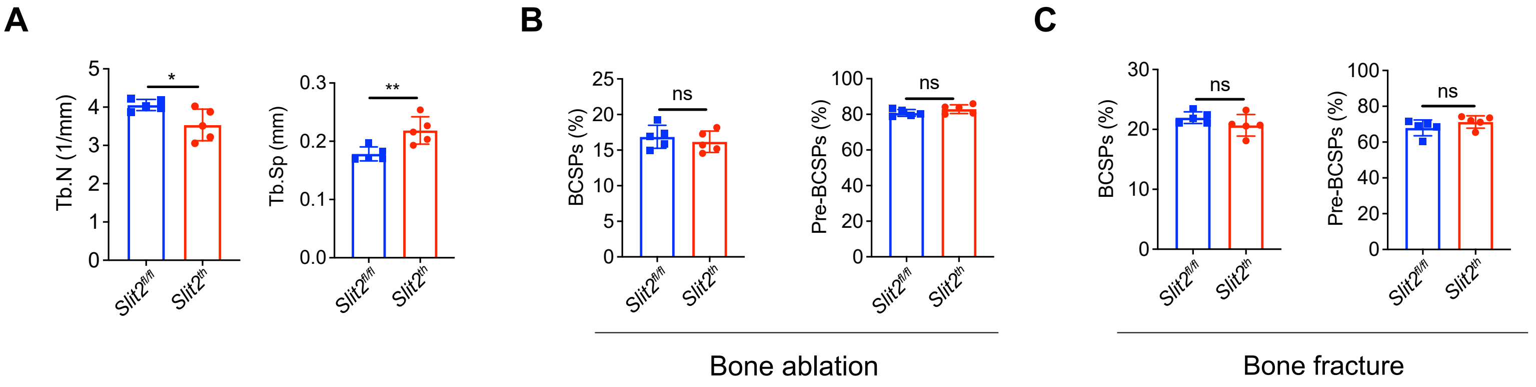
A
B
C
Tb.N (1/mm)
Tb.Sp (mm)
BCSPs (%)
Pre-BCSPs (%)
*
**
ns
Slit2fl/fl
Slit2th
Bone ablation
Bone fracture

Supplementary

Supplementary Figure 1. Skeleton-derived SLIT2 does not affect bone mass in vivo.

(**A**) mRNA levels of *Slit2* in osteoblast and osteoclast differentiation using brain as a positive control. Real-time PCR was utilized. n = 3 per group.

(**B**) The protein expression level of SLIT2 in brain, osteoblast and osteoclast was analyzed by immunoblotting. n = 3 per group.

(**C**) mRNA levels of *Slit2* in osteoblasts and sympathetic neurons was analyzed by real-time PCR in *Slit2*$^{fl/fl}$ and *Slit2*^{osx} mice. n = 4 per group.

(**D**) The protein expression level of SLIT2 in osteoblasts and sympathetic neurons were analyzed by immunoblotting in *Slit2*$^{fl/fl}$ and *Slit2*^{osx} mice.

(**E**-**F**) Representative μ-computed tomography (μCT) images of the trabecular bone in the distal femur (**E**) and relative quantitative analysis of bone volume/total volume (BV/TV) and relative quantitative analysis of bone parameters (**F**) in control mice (Osx-cre and *Slit2*$^{fl/fl}$) and *Slit2*^{osx} male mice at 8 weeks of age. Osx-cre, n = 7; *Slit2*$^{fl/fl}$, n = 7; *Slit2*^{osx}, n = 7; Scale bars, 500 μm.

(**G**) Representative confocal images of femur sections from 3-week-old *Slit2*$^{fl/fl}$ and *Slit2*^{osx} male mice with CD31 (green) and EMCN (red). The growth plate is marked with a dashed line. Scale bars, 300 μm.

(**H**) Representative flow cytometry plots and relative frequency of CD31^{hi}EMCN^{hi} endothelial cells from the femurs of 3-week-old *Slit2*$^{fl/fl}$ and *Slit2*^{osx} male mice. n = 4 per group.

(**I**) Representative ALP staining and alkaline phosphatase staining (ALP) activity in *Slit2*$^{fl/fl}$ and *Slit2*^{osx} primary osteoblasts at day 7 of osteogenic induction. n = 3 per group; Scale bars, 1 mm.

(**J**) Representative Alizarin red staining and quantitative analysis of mineralization in *Slit2*$^{fl/fl}$ and *Slit2*^{osx} primary osteoblasts at day 21 of osteogenic induction. n = 3 per group; Scale bars, 1 mm.

All error bars indicate mean ± SEM.

Supplementary Figure 2. Skeleton-derived SLIT2 does not affect bone mass in vivo.

(**A**) mRNA levels of *Slit2* in bone marrow stromal cells (BMSCs) and sympathetic neurons were analyzed by real-time PCR in *Slit2*$^{fl/fl}$ and *Slit2*^{prx1} mice. n = 4 per group.

(**B**) The protein expression level of SLIT2 in bone marrow stromal cells (BMSCs) and sympathetic neurons from *Slit2*$^{fl/fl}$ and *Slit2*^{prx1} mice was analyzed by immunoblotting.

(C-D) Representative μCT images of the trabecular bone in the distal femur (**C**) and BV/TV with relative quantitative analysis of bone parameters (**D**) in *Slit2*$^{fl/fl}$ and *Slit2*^{prx1} male mice at 8 weeks of age. n = 6 per group; Scale bars, 500 μm.

All error bars indicate mean ± SEM. ***p < 0.001.

Supplementary Figure 3. *Slit2*^{syn1} mice display a specific deletion of *Slit2* in neurons.

(**A**) Representative immunofluorescence images of nerve fibers in the bones of

3-week-old Syn1-cre; Ai9 mice to trace Syn1-cre targeted cells. Scale bars, 500 μm.

(**B**) mRNA levels of *Slit2* in sympathetic neurons and osteoblasts were analyzed by real-time PCR in *Slit2*$^{fl/fl}$ and *Slit2*^{syn1} male mice at 3 weeks of age. n = 4 per group.

(**C**) The Protein expression level of SLIT2 in *Slit2*$^{fl/fl}$ and *Slit2*^{syn1} mice in sympathetic neurons and osteoblasts was analyzed by immunoblotting.

(**D**) Serum CTX levels in 8-week-old male *Slit2*$^{fl/fl}$ and *Slit2*^{syn1} mice were measured and quantified by ELISA. n = 4 per group.

All error bars indicate mean ± SEM. ****p < 0.0001.

Supplementary Figure 4. *Slit1*$^{-/-}$ mice do not display a significant change in bone mass.

(**A**-**B**) Representative μCT images of the trabecular bone in the distal femur (**A**) and relative quantitative analysis of bone parameters (**B**) in *Slit1*$^{+/+}$ and *Slit1*$^{-/-}$ male mice at 8 weeks of age. n = 8 per group; Scale bars, 500 μm.

All error bars indicate mean ± SEM.

Supplementary Figure 5. *Slit2*^{syn1} mice do not display a significant change in sensory innervation in the femur.

(**A**) Representative confocal images of immunofluorescence staining with CGRP (green) and DAPI (blue) and relative quantitative analysis of CGRP$^+$ sensory nerves in the femur sections from 3-week-old *Slit2*$^{fl/fl}$ and *Slit2*^{syn1} male mice, the arrowheads point to CGRP$^+$ sensory nerve fibers. n = 10 per group;

Scale bars, 500 μm.

All error bars indicate mean ± SEM.

Supplementary Figure 6. *Slit2*th and *Slit2*^{adv} mice display specific deletion of *Slit2* in neuron subtypes.

(**A**) Representative immunofluorescence images showing the colocalization of TH^{+} sympathetic nerve fibers with SLIT2 in the bones of 3-week-old WT mice. Scale bars, 250 μm.

(**B**) Representative immunofluorescence images showing colocalization of CGRP^{+} sensory nerve fibers with SLIT2 in the bones of 3-week-old WT mice. Scale bars, 250 μm.

(**C**) Representative immunofluorescence images of sympathetic nerve fibers in the bones of 3-week-old Th-cre; Ai9 mice using lineage tracing. Scale bars, 500 μm.

(**D**) Representative immunofluorescence images of sensory nerve fibers in the bones of 3-week-old Advillin-cre; Ai9 mice using lineage tracing. Scale bars, 500 μm.

(**E**) mRNA levels of *Slit2* in sympathetic neurons and osteoblasts were analyzed by real-time PCR in *Slit2*$^{fl/fl}$ and *Slit2*th mice. n = 4 per group.

(**F**) The protein expression level of SLIT2 in sympathetic neurons and osteoblasts from *Slit2*$^{fl/fl}$ and *Slit2*th mice was analyzed by immunoblotting.

(**G**) mRNA levels of *Slit2* in sensory neurons and osteoblasts from *Slit2*$^{fl/fl}$ and *Slit2*^{adv} mice were analyzed by real-time PCR. n = 4 per group.

(**H**) The protein expression level of SLIT2 in sensory neurons and osteoblasts from *Slit2*$^{fl/fl}$ and *Slit2*^{adv} mice was analyzed by immunoblotting.

All error bars indicate mean ± SEM. ***p < 0.001.

Supplementary Figure 7. *Slit2*th mice exhibit increased periosteal sympathetic nerve innervation.

(**A**-**B**) Representative µCT images of the trabecular bone in the distal femur (**A**) and BV/TV with relative quantitative analysis of bone parameters (**B**) in *Slit2*$^{fl/f}$ and *Slit2*th female mice at 8 weeks of age. *Slit2*$^{fl/fl}$, n= 6; *Slit2*th, n=7; Scale bars, 500 µm.

(**C**) Two-way analysis of variance was performed to assess the effects of sex (male/female), genotype (*Slit2*$^{fl/fl}$ vs. *Slit2*th), and their interaction on bone volume fraction (BV/TV). DF: degrees of freedom; Sum Sq: sum of squares; Mean Sq: mean square; F value: F-statistic; P value: statistical significance. Both sex and genotype had significant effects on BV/TV (P < 0.001), while no significant interaction was observed between sex and genotype (P = 0.268).

(**D**) Representative confocal images of immunofluorescence staining with TH (red) and DAPI (blue) and quantitative analysis of relative TH^+ sympathetic nerves in the femur sections from 3-week-old *Slit2*$^{fl/fl}$ and *Slit2*th female mice. *Slit2*$^{fl/fl}$, n= 11; *Slit2*th, n=13; Scale bars, 500 µm.

(**E**) Representative confocal images of immunofluorescence staining with TH (red) and DAPI (blue) and quantitative analysis of relative TH^+ sympathetic nerves in the femur sections from 3-week-old *Slit2*$^{fl/fl}$ and *Slit2*^{adv} male mice. n

= 8 per group; Scale bars, 500 μm.

(**F**) Immunofluorescence staining of TH (red) sympathetic nerves in femoral cross-sections of 3-week-old *Slit2*$^{fl/fl}$ and *Slit2*th male mice, along with corresponding quantitative analysis. *Slit2*$^{fl/fl}$ sections, n =12; *Slit2*th sections, n =11, Scale bars, 200 μm.

All error bars indicate mean ± SEM.*p < 0.05, **p < 0.01, ***p < 0.001.

Supplementary Figure 8. *Slit2*th mice show no significant changes in *Slit1* and *Slit3* expression in sympathetic neurons.

(**A**) The protein expression level of SLIT1 in sympathetic neurons from *Slit2*$^{fl/fl}$ and *Slit2*th mice was analyzed by immunoblotting. Mouse placental tissue was used as positive control.

(**B**) The protein expression level of SLIT3 in sympathetic neurons from *Slit2*$^{fl/fl}$ and *Slit2*th mice was analyzed by immunoblotting.

(**C**) mRNA levels of *Slit3* in sympathetic neurons from PCR *Slit2*$^{fl/fl}$ and *Slit2*th mice was analyzed by real-time PCR. n = 4 per group.

(**D**) Representative images of Nissl staining of brain tissue from 3-week-old *Slit2*$^{fl/fl}$ and *Slit2*th mice. Scale bars, 1 mm.

All error bars indicate mean ± SEM.

Supplementary Figure 9. *Slit2*th mice do not display a significant change in sensory innervation in the femur.

(**A**) Representative confocal images of immunofluorescence staining with CGRP (green) and DAPI (blue) and quantitative analysis of relative CGRP^{+}

sensory nerves in the femur sections from 3-week-old *Slit2*$^{fl/fl}$ and *Slit2*th male mice. n = 8 per group; Scale bars, 1 mm.

(**B**) Serum CTX levels in 8-week-old male *Slit2*$^{fl/fl}$ and *Slit2*th mice were measured and quantified by ELISA. n = 4 per group.

All error bars indicate mean ± SEM.

Supplementary Figure 10. The distance between TH^{+} sympathetic nerve fibers and regions containing SSCs in the femur.

(**A**-**D**) Representative images and quantification of the distance between TH^{+} sympathetic nerve fibers and regions containing SSCs in the femur through whole-tissue TH^{+} sympathetic nerve immunofluorescence analysis after optical clearance of bone tissue in 3-week-old *Slit2*$^{fl/fl}$ and *Slit2*th male mice. Refer to figure 2F; Scale bars = 500 μm; Grow plate resting zone, n=30; Metaphysis zone, n=30; Endosteum zone, n=9; Periosteum zone, n=9.

All error bars indicate mean ± SEM.

Supplementary Figure 11. Gating strategy for flow cytometry analysis of SSCs.

(**A**) Schematic representation of the strategy used for flow cytometry for analysis of SSCs.

(**B**) Representative flow cytometry plots and relative frequency of SSCs from the femurs of 3-week-old *Slit2*$^{fl/fl}$ and *Slit2*^{syn1} male mice. n= 5 per group.

(**C**) Representative flow cytometry plots and relative frequency of SSCs from the femurs of 3-week-old *Slit2*$^{fl/fl}$ and *Slit2*th female mice. n= 5 per group.

(**D**) Relative frequency of pre-BCSP (pre-**Bone Cartilage Stromal Progenitor, CD200^{-}CD105^{-}**) and BCSP (**Bone Cartilage Stromal Progenitor, CD200^{-}CD105^{+}**) from the femurs of 3-week-old *Slit2*$^{fl/fl}$ and *Slit2*^{syn1} male mice by flow cytometry. n = 5 per group.

(**E**) Relative frequency of pre-BCSP and BCSP from the femurs of 3-week-old *Slit2*$^{fl/fl}$ and *Slit2*th male mice by flow cytometry. n = 5 per group.

All error bars indicate mean ± SEM, **p < 0.01.

Supplementary Figure 12. Sympathetic innervation negatively controls PSC abundance.

(**A**) Representative flow cytometry plots and relative frequency of PSCs (Periosteal stem cells) from the femurs of 3-week-old *Slit2*$^{fl/fl}$ and *Slit2*th male mice. *Slit2*$^{fl/fl}$, n=13; *Slit2*th n=12.

(**B**) mRNA levels of *c-MYC* and Cyclin D1 (*CCND1*) in SSCs were analyzed by real-time PCR in *Slit2*$^{fl/fl}$ and *Slit2*th mice. n = 4 per group.

All error bars indicate mean ± SEM. *p < 0.05, **p < 0.01.

Supplementary Figure 13. The abundance of SSCs decreases while the innervation of TH^{+} sympathetic nerves increases during early postnatal life.

(**A**) Representative confocal images of immunofluorescence staining with TH (red) and DAPI (blue) sections in the femurs of wild-type, *Slit2*^{syn1} and *Slit2*th male mice at postnatal day 0 (P0), P7, P14 and P21. Quantitative analysis of TH^{+} sympathetic nerves is provided. *Slit2*$^{fl/fl}$ (P0), n = 4; *Slit2*$^{fl/fl}$ (P7), n = 5; *Slit2*$^{fl/fl}$

(P14), n =6; *Slit2*$^{fl/fl}$ (P21), n = 6; *Slit2*^{syn1} (P0), n = 4; *Slit2*^{syn1} (P7), n = 4; *Slit2*^{syn1} (P14), n =5; *Slit2*^{syn1} (P21), n = 5; *Slit2*th, n = 5 per group; Scale bars, 500 μm.

(**B**) Representative flow cytometry plots and relative frequency of SSC from the femurs of postnatal day 0 (P0), P7, P14 and P21 wild-type male mice. P0, n=3; P7, n=3; P14, n=4; P21, n=4.

All error bars indicate mean ± SEM.

Supplementary Figure 14. Additional analysis of orthotopic SSC niche engraftment assays.

(**A**) Representative flow cytometry showing the relative frequency of femur SSCs in 3-week-old *Slit2*th male mice after sympathectomy surgery. Sham, n= 6; Den, n=7; Sham: Sham surgery group; Den: Denervation group.

(**B**) Representative confocal images of immunofluorescence staining with TH (red) with DAPI (blue) and quantitative analysis of TH^{+} sympathetic nerves in femurs sections from 3-week-old *Slit2*th male mice after sympathectomy surgery. Sham, n= 6; Den, n=5; Scale bars, 500 μm.

(**C**) Schematic of the flow cytometry sorting strategy for tdTomato^{+} SSCs sorted from *mT/mG* mice.

(**D**-**E**) Representative immunofluorescence images of TH^{+} sympathetic nerves in the femurs of *Slit2*$^{fl/fl}$-MIP-GFP and *Slit2*th-MIP-GFP male mice two days after bone marrow cavity transplantation of tdTomato^{+} SSCs. Quantitative analysis of the distance between tdTomato^{+} cells and the nearest TH^{+} nerve fibers. n=3 per group; Scale bars, 200 μm.

(**F**) Representative flow cytometry plots and quantitative analysis of the relative frequency of tdTomato^{+} BCSPs from the femurs of *Slit2*$^{fl/fl}$-MIP-GFP and *Slit2*th-MIP-GFP male mice 4 weeks after SSC transplantation. n= 4 per group.

(**G**) Representative Crystal violet staining and quantitative analysis of CFU formation in SSCs from the femurs of 3-week-old *Slit2*$^{fl/fl}$ and *Slit2*th male mice. n = 3 per group; Scale bars, 5 mm.

(**H**) Representative Alizarin red staining and quantitative analysis of mineralization in SSCs from the femurs of 3-week-old *Slit2*$^{fl/fl}$ and *Slit2*th male mice at day 21 of osteogenic induction. n = 4 per group; Scale bars, 1 mm.

All error bars indicate mean ± SEM. *p < 0.05, ***p < 0.001, ****p < 0.0001.

Supplementary Figure 15. There was no significant difference in the osteogenic ability of SSCs in the renal subcapsule of *Slit2*$^{fl/fl}$ and *Slit2*th mice.

(**A**) Schematic of the experimental model of renal subcapsular transplantation of SSCs. Approximately 1×10^4 SSCs were transplanted beneath the renal capsule on one side of each recipient mouse.

(**B**) Representative images of μCT and quantitative analysis of bone parameters 4 weeks after SSC subcapsular transplantation in the mouse kidney. n= 4 per group; Scale bars, 800 μm.

All error bars indicate mean ± SEM.

Supplementary Figure 16. Additional analysis of epinephrine and norepinephrine signaling in *Slit2*$^{fl/fl}$ and *Slit2*th mice.

(**A**) Transcriptomic analysis of adrenergic receptors in primary skeletal stem cells. n = 3.

(**B**) Representative flow cytometry and quantitative analysis of the relative frequency of SSCs isolated from the femurs of 3-week-old WT and *Adrb2*$^{-/-}$ male mice. n = 4.

(**C**) Timeline for Salbutamol or Propranolol treatment in *Slit2*$^{fl/fl}$ and *Slit2*th mice.

(**D**-**E**) Representative flow cytometry images and quantitative analysis of the relative frequency of SSCs in the femurs of 3-week-old *Slit2*$^{fl/fl}$ and *Slit2*th control male mice, or *Slit2*$^{fl/fl}$ and *Slit2*th male mice treated with Salbutamol or Propranolol. Vehicle, n = 3; Salbutamol, n = 4; Propranolol, n = 4.

(**F**-**G**) The concentrations of epinephrine (EPI) and noradrenaline (NE) in the serum of 8-week-old *Slit2*$^{fl/fl}$, *Slit2*^{syn1} and *Slit2*th male mice were measured using ELISA. n = 4.

All error bars indicate mean ± SEM.

Supplementary Figure 17. FSTL1 secreted from sympathetic nerves inhibits SSC self-renewal and osteogenesis.

(**A**) Crystal violet staining and quantitative analysis of colonies formed by cultured SSCs treated with recombinant murine FSTL1 (mFSTL1) or vehicle. mFSTL1 was used at 200 ng/ml; n = 3 per group; Scale bars, 5 mm.

(**B**) Alizarin red staining and quantitative analysis of the mineralization activity of SSCs stimulated with mFSTL1 or vehicle. mFSTL1 was used at 200 ng/ml; n = 4 per group; Scale bars, 1 mm.

All error bars indicate mean ± SEM. **p < 0.01, ****p < 0.0001.

Supplementary Figure 18. Sympathetic hyperinnervation disrupts bone regeneration and bone fracture healing by impairing SSC expansion.

(**A**) Quantitative analysis of bone parameters in the regeneration area of femurs from 6-week-old *Slit2*$^{fl/fl}$ and *Slit2*th male mice 7 days after bone marrow ablation. n=5 per group.

(**B**) Relative frequencies of pre-BCSP and BCSP were determined by flow cytometry 7 days after femoral bone marrow ablation in 6-week-old *Slit2*$^{fl/fl}$ and *Slit2*th male mice. n=5 per group.

(**C**) Relative frequencies of pre-BCSPs and BCSPs were determined by flow cytometry 14 days after femur fracture in 6-week-old *Slit2*$^{fl/fl}$ and *Slit2*th male mice. n=5 per group.

All error bars indicate mean ± SEM.

Supplementary Table1: Key resource information

REAGENT or RESOURCE	SOURCE	IDENTIFIER
**Antibodies**		
CD45 BUV395	BD Biosciences	Cat#564279; RRID: AB_2651134
TER119 BUV395	BD Biosciences	Cat#563827; RRID: AB_2738438
Ly-51(6C3) PerCP-cy5.5	Biolegend	Cat#108316; RRID: AB_2632658
CD90.2 Percp-cy5.5	Biolegend	Cat#105338; RRID: AB_2571945
CD31 FITC	Biolegend	Cat#102406; RRID: AB_312901
CD51 PE	Invitrogen	Cat#12-0512-83; RRID: AB_465705
CD200 BV421	BD Biosciences	Cat#565547; RRID: AB_2739289
CD105 PE/cy7	Invitrogen	Cat#25-1051-82; RRID: AB_2573380
Endomucin-eFlour660(APC)	Invitrogen	Cat#50-5851-80; RRID: AB_11220069
Anti-Calcitonin Gene Related Peptide antibody produced in rabbit	Sigma-Aldrich	Cat#C8198; RRID: AB_259091
Anti-Tyrosine Hydroxylase Antibody	Millipore	Cat#AB152; RRID: AB_390204
Mouse/Rat CD31/PECAM-1 Antibody	R&D Systems	Cat#AF3628; RRID: AB_2161028
Endomucin Polyclonal Antibody	Invitrogen	Cat#PA5-47648; RRID: AB_2576794
CD16/CD32 Monoclonal Antibody	Thermo Fisher Scientific	Cat#14-0161-86; RRID: AB_467135
Donkey anti-Rabbit IgG (H+L) Highly Cross-Adsorbed Secondary Antibody, Alexa Fluor™ 488	Invitrogen	Cat#A21206; RRID: AB_2535792
beta-3 Tubulin Monoclonal Antibody (2G10)	Invitrogen	Cat#MA1-118; RRID: AB_2536829

SLIT2-Specific Rabbit Polyclonal Antibody	Proteintech	Cat#20217-1-AP; RRID: AB_10805766
Slit2(F-7)	Santa Cruz	Cat#sc-514499; RRID: AB_3662869
CD200 / OX2 antibody [MRC OX90]	Abcam	Cat# ab33734; RRID: AB_726239
FSTL1 Polyclonal antibody	Invitrogen	Cat#PA5-31113; RRID: AB_2548587
FABP4 Antibody	Proteintech	Cat#15872-1-AP; RRID: AB_2102440
COL2A1(M2139)	Santa Cruz	Cat#sc-52658; RRID: AB_2082344
Donkey Anti-Rabbit IgG (H+L) Highly Cross-Adsorbed Secondary Antibody, Alexa Fluor™ 647	Invitrogen	Cat# A-31573; RRID: AB_2536183
Donkey anti-Rat IgG (H+L) Highly Cross-Adsorbed Secondary Antibody, Alexa Fluor™ 594	Invitrogen	Cat# A-21209; RRID: AB_2535795
Goat anti-Rabbit IgG (H+L) Cross-Adsorbed Secondary Antibody, Alexa Fluor™ 594	Invitrogen	Cat# A-11012; RRID: AB_2534079
Embigin Antibody, anti-mouse, APC, REAfinity™	Miltenyi Biotec	Cat#130-117-527; RRID: AB_2751395
Slit1(G-4)	Santa Cruz	Cat#sc-376756; RRID: AB_3662870
Human/ Mouse/ Rat Slit3 Antibody	R&D Systems	Cat#AF3629; RRID: AB_663857
Beta Actin Antibody	Proteintech	Cat# 66009-1-Ig; RRID: AB_2687938
Alexa Fluor® 647 mouse anti-Ki-67 (Clone B56)	BD Biosciences	Cat#561126; RRID: AB_10611
Goat Anti-Mouse Osteopontin Polyclonal antibody	R&D Systems	Cat#AF808; RRID: AB_2194992
ITM2A Antibody	Proteintech	Cat#18306-1-AP; RRID: AB_2249412
**Chemicals, peptides, and recombinant proteins**		

α-MEM	Gibco	Cat#C12571500BT
L-Ascorbic acid	Yeasen	Cat#60374ES60
β-Glycerophosphate disodium salt	Sigma	Cat#G5422-25G
RANKL	R&D	Cat#462-TEC
M-CSF	R&D	Cat#416-ML/CF
Tris	BioFroxx	Cat#1115GR500
Fast blue BB salt hemi (zinc chlocide)	Sigma	Cat#F388-1G
AS - BI	Sigma- Aldrich	Cat#N2125-5G
Fast Red- violet- LB salt	Sigma- Aldrich	Cat#F3381-1G
Alizarin Red S	Sigma- Aldrich	Cat#A5533-25G
Fetal Bovine Serum	Gibco	Cat#1009-148
6-hydroxydopamine (6-OHDA)	Sigma	Cat#H4381-100MG
Hank's Buffer	Procell	Cat#PB180321
DME/F12	HyClone	Cat#SH30023.01
0.25%tryptase	Beyotime	Cat#C0201-100ml
Polylysine	Sigma	Cat#P4707
Colagenase I	Sigma	Cat#SCR103
B27	Gibco	Cat#A1486701
DMEM	Cytiva	Cat#SH30243.01
Neurobasal medium	Gibco	Cat#21103049
Trlzol	Invitrogen	Cat#15596026
SYBR Green Power PCR Master Mix	Invitrogen	Cat#A25777
Calcein	Sigma- Aldrich	Cat#C0875-10G
Alcian blue 8GX+	Sigma- Aldrich	Cat#A3157-10G
Hematoxylin Staining Solution	Yuanye	Cat#517-28-2
Paraformaldehyde (PFA)	Macklin	Cat#P804536-2.5kg
NON-Fat powered milk	BBI	Cat#A600669-6250
HEPES	Corning	Cat#25-060-CI
toluidine blue	Macklin	Cat#T818873-5g
colagenase A	Roche	Cat#11088793001
dispase II	Roche	Cat#04942078001
EDTA	Rhawh	Cat#RH505086
OCT compound	Sakura	Cat#4583
Triton X-100	Diamond	Cat#A110694-0100
DAPI	BioFroxx	Cat#1155MG010
recombinant protein Fstl1	Biotechne	Cat#1738-FN-050

Heparin sodium	BBI	Cat#A603251-0001
Sucrose	Sangon Biotech	Cat#A502792-0005
Methanol	Hushi	Cat#100141190
Hydrogen peroxide 30% aqueous solution	Hushi	Cat#10011218
Dimethyl sulfoxide	Sigma-Aldrich	Cat#34869-500ML
Deoxycholic acid, sodium salt	BBI	Cat#A600150-0050
Sodium azide	Sigma-Aldrich	Cat#S8032
Agarose	BioFroxx	Cat#1110GR100
Dichloromethane	Hushi	Cat#80047318
Dibenzyl Ether	TCI	Cat#B0418
Potassium hydroxide	Hushi	Cat#10017018
Glacial acetic acid	Hushi	Cat#10000218
Glycerol	Solarbio	Cat#G8190
ACK lysis buffer	Gibco	Cat#A1049201
Matrigel	Corning	Cat#354230
Sodium Acetate Anhydrous	Hushi	Cat#10018818
L-(+)Tartaric Acid	Hushi	Cat#30169818
Pararosaniline Chloride	Yuanye	Cat#S19241-25g
PMSF	Solarbio	Cat#P0100
PVDF Membrane	Millipore	Cat#IPVH00010
Silver nitrate	Sigma-Aldrich	Cat#209139-25G
crystal violet	MCE	Cat#HY-B0324A
Nissle	Beyotime	Cat#C0117
Recombinant Mouse Follistatin-like 1 Protein, CF	R&D	Cat#1738-FN-050
**Critical commercial assays**		
Fstl1-ELISA	Cloud-Clone Corp	Cat#SEJ085Mu
NA/NE(Noradrenaline/Norepinephrine) ELISA Kit	Elabscience	Cat#E-EL-0047c
EPI(Epinephrine/Adrenaline) ELISA Kit	Elabscience	Cat#E-EL-0045
**Experimental models: Organisms/strains**		
C57BL/6-Slit2$^{flox/flox}$	From the group of Prof. Alain Chédotal, University of Marie Curie, France	N/A
C57BL/6-Tg (Dbh-cre/ERT2)198.1Hroh/J	From the group of Prof. Jing Yang, School of Life Sciences, Peking	N/A

	University, China	
STOCK Adrb1^{tm1Bkk} Adrb2^{tm1Bkk}/J	From the group of Prof. Kairui Mao, School of Life Sciences, Xiamen University, China	N/A
C57BL/6JGpt-Fstl1^{em1Cflox}/Gpt	GemPharmatech	T018356
B6. Cg-7630403G23RikTg (Th-cre)1Tmd/J	The Jackson Laboratory	JAX:008601
B6.129P2-Avil$^{tm2(cre)Fawa}$/J	The Jackson Laboratory	JAX:032536
B6. Cg-Tg(Syn1-cre)671Jxm/J	The Jackson Laboratory	JAX:003966
C57BL/6	From Xiamen University Laboratory Animial Center	N/A
**Oligonucleotides**		
PCR primers and qPCR primers, see Supplementary Table	This study	N/A
**Software and algorithms**		
NRecon Version 1.6	Bruker	https://www.bruker.com/service/support
CTan Version 1.15	Bruker	https://www.bruker.com/service/support
FlowJo V10	FlowJo (v10) Tree Star Inc	N/A
image J	Fiji.sc	N/A
Imaris software	Bitplane	https://imaris.oxinst.com/
Osteomeasure System	Osteometrics	N/A
GraphPad Prism (v6.0a)	GraphPad Software Inc	N/A
pheatmap (v1.0.12)	Raivo Kolde	https://mirrors.ustc.edu.cn/CRAN/web/packages/pheatmap/index.html
ggplot2 (v3.5.1)	Hadley Wickham et al.	https://cran.rstudio.com/web/packages/ggplot2/inde

		x.html
clusterProfiler (v 4.12.6)	Xu S et al.	https://www.bioconductor.org/packages/release/bioc/html/clusterProfiler.html
HISAT2 (v2.2.1)	Christopher Bennett et al.	https://daehwankimlab.github.io/hisat2/
edgeR (v 4.2.2)	Chen Y et al.	https://bioconductor.org/packages/release/bioc/html/edgeR.html
GSEA software (v4.3.2)	Tamayo et al.	https://www.gsea-msigdb.org/gsea/index.jsp
MSigDB Database (v2024.1)	Tamayo et al.	https://www.gsea-msigdb.org/gsea/msigdb/index.jsp

Supplementary Table2: List of primers used for PCR and qRT-PCR of mouse genes:

Primer	Application	Sequence
Slit2 flox_0230	PCR	5'-TGGTTCACAAACACGAGTCAATTCC-3'
Slit2 flox_0231	PCR	5'-ATGCCTCTTTATGTCCACAGCTCTG-3'
TH-cre-FW	PCR	5'-CGATGCAACGAGTGATGAGG-3'
TH-cre-RV	PCR	5'-CGCATAACCAGTGAAACAGC-3'
Advilli-cre-42709	PCR	5'-AATGGCTCCCTGTTCACTGT-3'
Advilli-cre-42710	PCR	5'-TGACTAGGTAGAGGTGCAAATGTC-3'
Advilli-cre-oIMR9074	PCR	5'-AGGCAAATTTTGGTGTACGG-3'
Syn1-FW	PCR	5'-CTCAGCGCTGCCTCAGTCT-3'
Syn1-RV	PCR	5'-GCATCGACCGGTAATGCA-3'
Osx-FW	PCR	5'-AGGCAGGTGCCTGGACAT-3'
Osx-RV	PCR	5'-CTCTTCATGAGGAGGACCCT-3'
Prx1-FW	PCR	5'-TCTCTGGCTCTGATGTTGGCA-3'
Prx1-RV	PCR	5'-CGCATAACCAGTGAAACAGC-3'
Dbh^{CreERT2} -73768	PCR	5'-CGAACCTCATCACTCGTTG-3'
Dbh^{CreERT2} -75651	PCR	5'-ATGTGTCATTAGTGCCAATTAGG-3'
Dbh^{CreERT2} -75653	PCR	5'-ACAGCATCATCCCATCCCTT-3'
Dbh^{CreERT2} -75654	PCR	5'-CCGTTCACCTCTGGCTTC-3'
Fstl1-FW	PCR	5'-GATGTCTGGATGTGTCTCCAGTGTT-3'
Fstl1-RV	PCR	5'-TTGTTCCCCTGTGAAGAGTGACTTTC-3'
MIP-GFPtg(Weir)-F	PCR	5'-TGGAAACTGCAGCTTCAG-3'
MIP-GFPtg(Weir)-R	PCR	5'-GTCCAGCTCGACCAGGATGG-3'
MIP-GFPtg(Bell)-F	PCR	5'-GAAGACAATAGCAGGCATGCTG-3'
MIP-GFPtg(Bell)-R	PCR	5'-ACTGGGCTTACATGGCGATACTC-3'
Ai9-oIMR9020	PCR	5'-AAGGGAGCTGCAGTGGAGTA-3'
Ai9-oIMR9021	PCR	5'-CCGAAAATCTGTGGGAAGTC-3'
Ai9-oIMR9103	PCR	5'-GGCATTAAAGCAGCGTATCC-3'
Ai9-oIMR9105	PCR	5'-CTGTTCCTGTACGGCATGG-3'
mT/mG-Common	PCR	5'-CTTTAAGCCTGCCCAGAAGA-3'
mT/mG-Mut Forward	PCR	5'-TAGAGCTTGCGGAACCCTTC-3'
mT/mG-WT Forward	PCR	5'-AGGGAGCTGCAGTGGAGTAG-3'
Adrb2-oIMR2060	PCR	5'-CACGAGACTAGTGAGACGTG-3'
Adrb2-oIMR7429	PCR	5'-ACCAAGAATAAGGCCCGAGT-3'
Adrb2-oIMR7430	PCR	5'-CCGGGAATAGACAAAGACCA-3'
Hprt-FW	qRT-PCR	5'-CTGGTGAAAAGGACCTCTCGAAG-3
Hprt-RV	qRT-PCR	5'-CCAGTTTCACTAATGACACAAACG-3'

Slit2-FW	qRT-PCR	5’-ACCGTCTGAGATGTATCCCTCC-3’
Slit2-RV	qRT-PCR	5’-GCTGACAAGTCATTGAAGGCACC-3’
Slit3-FW	qRT-PCR	5’-GCATGGCAATGATGTCTCCACC-3’
Slit3-RV	qRT-PCR	5’-TCCTTGTAGCCAGTCTTCACCC-3’
Fstl1-FW	qRT-PCR	5’-CTGTCTGATGAGAACGCTGACTG-3’
Fstl1-RV	qRT-PCR	5’-AGACACAGCGATTGCAGTCCAC-3’
Ccnd1-FW	qRT-PCR	5’-GCAGAAGGAGATTGTGCCATCC-3’
Ccnd1-RV	qRT-PCR	5’-AGGAAGCGGTCCAGGTAGTTCA-3’
Myc-FW	qRT-PCR	5’-TCGCTGCTGTCCTCCGAGTCC-3’
Myc-RV	qRT-PCR	5’-GGTTTGCCTCTTCTCCACAGAC-3’
